# On the Trail of *Tetu1*: Genome-Wide Discovery of CACTA Transposable Elements in Sunflower Genome

**DOI:** 10.3390/ijms21062021

**Published:** 2020-03-16

**Authors:** Maria Ventimiglia, Claudio Pugliesi, Alberto Vangelisti, Gabriele Usai, Tommaso Giordani, Lucia Natali, Andrea Cavallini, Flavia Mascagni

**Affiliations:** Department of Agriculture, Food and Environment (DAFE), University of Pisa, Via del Borghetto, 80-56124 Pisa, Italy; maria.ventimiglia@phd.unipi.it (M.V.); claudio.pugliesi@unipi.it (C.P.); alberto.vangelisti@agr.unipi.it (A.V.); gabriele.usai@agr.unipi.it (G.U.); tommaso.giordani@unipi.it (T.G.); lucia.natali@unipi.it (L.N.); andrea.cavallini@unipi.it (A.C.)

**Keywords:** CACTA, class II transposons, CACTA classification, sunflower

## Abstract

Much has been said about sunflower (*Helianthus annuus* L.) retrotransposons, representing the majority of the sunflower’s repetitive component. By contrast, class II transposons remained poorly described within this species, as they present low sequence conservation and are mostly lacking coding domains, making the identification and characterization of these transposable elements difficult. The transposable element *Tetu1*, is a non-autonomous CACTA-like element that has been detected in the coding region of a *CYCLOIDEA* (*CYC*) gene of a sunflower mutant, tubular ray flower (*turf)*. Based on our knowledge of *Tetu1*, the publicly available genome of sunflower was fully scanned. A combination of bioinformatics analyses led to the discovery of 707 putative CACTA sequences: 84 elements with complete ends and 623 truncated elements. A detailed characterization of the identified elements allowed further classification into three subgroups of 347 elements on the base of their terminal repeat sequences. Only 39 encode a protein similar to known transposases (TPase), with 10 TPase sequences showing signals of activation. Finally, an analysis of the proximity of CACTA transposons to sunflower genes showed that the majority of CACTA elements are close to the nearest gene, whereas a relevant fraction resides within gene-encoding sequences, likely interfering with sunflower genome functionality and organization.

## 1. Introduction

Transposable elements (TEs) are dynamic genomic sequences capable of moving within the host genome by specific transposition mechanisms. TEs are very heterogeneous sequences and, with just a few exceptions, have been found in all eukaryotic genomes [1].

In plants, these elements often constitute the majority of genomic DNA, ranging from a minimum value of 15% of the smallest genomes as *Arabidopsis* spp., to more than 85% of the larger genomes, such as *Zea mays* ssp. *mays* and *Hordeum vulgare* [2,3]. Transposons have been conventionally classified: Class I elements, or retrotransposons (REs), by far the most numerous in animal and plant genomes, and Class II elements, or DNA transposons. RE elements transpose using a copy-and-paste mechanism, resulting in an identical copy of the starting element, whereas DNA transposons exploit a cut-and-paste mechanism, resulting in the excision of the sequence from the original locus [4]. In both cases, the TEs may integrate into new genomic loci, possibly affecting gene expression and function with consequent effects on phenotype, representing a crucial source of genetic variation [5,6,7,8]. These integrations may also result in peculiar phenomena where fragments of structural genes can be caught, rearranged, and subsequently transcribed by TEs during their transposition mechanisms, contributing to the evolution of novel chimeric genes [8,9].

Among TEs, CACTA represents one of the most widespread superfamilies of Class II transposons. CACTA is found in most genomes, spanning from algae [10] to vascular plants [11,12,13,14] and animals [15]. CACTA elements can reorganize host genomes, altering the structure and regulation of individual genes through several processes, such as transposition, insertion, excision, chromosome rupture, and ectopic recombination [16]. In maize, the classic *enhancer/suppressor mutator* (*En*/*Spm*) element was the first CACTA element identified independently by Peterson [17] and McClintock [18] and molecularly characterized by Pereira et al. [11].

The terminal regions of all identified CACTA TEs have a similar sequence organization. In particular, CACTA elements have terminal inverted repeats (TIRs) ranging from 10 to 28 bp, terminating with characteristic CACTA and TAGTG sequences flanked by target-site duplication (TSD) motifs, and several copies of sub-terminal repeats (TRs), ranging from 10 to 20 bp, which are repeated in a direct and inverted orientation. The low sequence conservation of TIRs and TRs makes the identification of CACTA elements difficult unless a transposase (TPase)-like domain is present in the body region. Based on the presence or absence of the TPase domain, CACTA elements can be considered autonomous or non-autonomous, respectively [19]. CACTA transposition is based on the formation of a hairpin structure with the two complementary TIRs and the binding of TPase to TRs [16]. If TIRs or TRs are deleted, transposition ceases. In Triticeae genomes, based on both structural similarity of TRs and phylogenetic analysis of multiple TR sequence alignments, CACTA TEs have been classified in distinct clades (e.g., *Caspar*, *TAT-1*, *Mandrake*, *Isaac*, *Balduin*, *Jorge,* and *Enac*) [19]. In the past few decades, many other CACTA elements were found in plants, such as *Tam1* from snapdragon, *Tgm1* from soybean, *Tdc1* from carrot, *Cs1* from sorghum, *Tpn1* from Japanese morning glory, *Ps1* from *Petunia hybrida*, *Pis1* from pea, *Tnr3* and *Tnr12* from rice, and *Cac1* from *Arabidopsis thaliana* [1,12,13,16,19,20].

**Figure 1:**
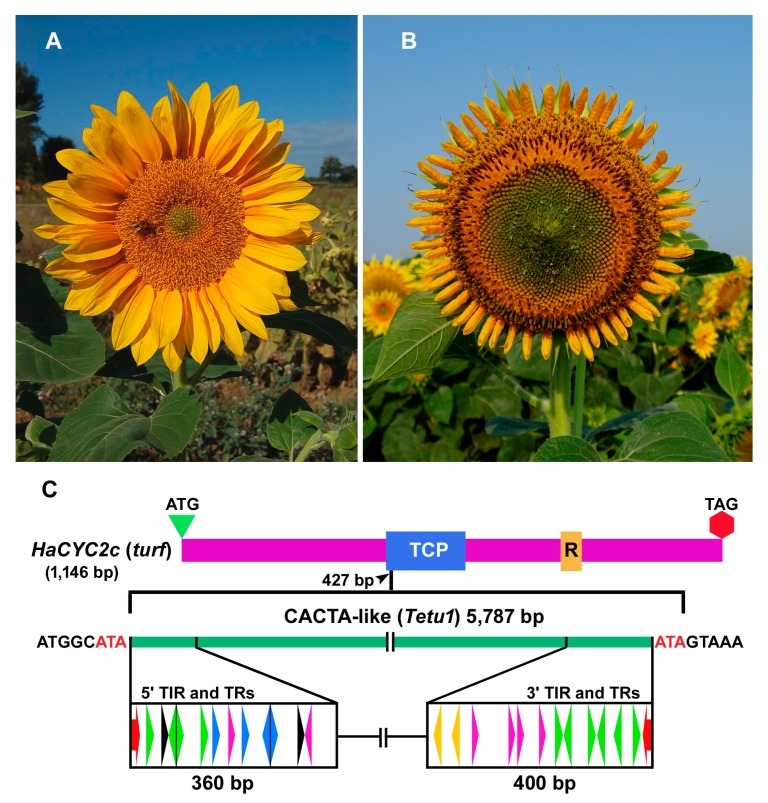
The phenotypic effect of the insertion of the CACTA-like transposable element (TE) in the *TEOSINTE BRANCHED1/CYCLOIDEA/PROLIFERATING NUCLEAR ANTIGEN CELL FACTOR (TCP) 1 and 2* motif of a *CYCLOIDEA* gene (*HaCYC2c*) controlling flower symmetry in sunflower [20]. (**A**) Inflorescence of normal sunflower with zygomorphic ray flowers. (**B**) Inflorescence of the *tubular ray flower* (*turf*) mutant. (**C**) Schematic representation of the coding region (CDS) of the *HaCYC2c* gene in the *turf* mutant. The *HaCYC2c* conserved domains, TCP and R, are boxed in blue and brown, respectively. The CACTA-like *transposable element of turf1* (*Tetu1*) is inserted into the basic motif of the TCP domain (427 bp after the start codon). The insertion originates a perfect three bp (ATA, in red) target site duplication (TSD). The structure of both 5′ (360 bp) and 3′ (400 bp) regions, recognized by terminal inverted repeats (TIRs) and sub-terminal repeats (TRs) of *Tetu1*, are depicted. The red arrows indicate the TIRs; the colored triangles indicate the TR regions of 9–11 bp units that are repeated in direct and inverted orientations. Each triangle color distinguishes an identical sequence in direct or inverted orientations. The TR sequences were identified by dot-plot analyses [19,20].

A *CYCLOIDEA* (*CYC*) gene of sunflower *(Helianthus annuus* L.), named *HaCYC2c*, plays a key role in establishing the zygomorphic symmetry of ray flowers (Figure 1A). The sunflower mutant *tubular ray flower* (*turf*) is characterized by a shift of sterile ray flowers from zygomorphic to near-actinomorphic and hermaphrodite disk-like flowers (Figure 1B). Previous studies showed that a spontaneous insertion of a non-autonomous CACTA-like element (Figure 1C), known as the *transposable element of turf1* (*Tetu1*), in the coding region of the *HaCYC2c* gene generates the *turf* mutant [20]. In particular, the insertion of *Tetu1* in the *TEOSINTE BRANCHED1/CYCLOIDEA/PROLIFERATING NUCLEAR ANTIGEN CELL FACTOR (TCP) 1 and 2* motif of the *turf-HaCYC2c* gene changes the reading frame for the encoded protein, inserting a premature stop codon in the sequence [20,21,22]. In sunflower, a similar phenotype, called *tubular-rayed*, caused by the insertion of a Class I TE in the *HaCYC2c* gene, was also described [23].

The genome of sunflower was estimated to be about 3.6 Gbp [24] and is composed of more than 81% TEs, of which the overwhelming majority are REs, mostly long terminal repeats (LTR) REs [25]. The DNA TEs were estimated to represent less than 3% of the nuclear DNA, whereas the CACTA superfamily represents about 0.13% [26]. Notably, only a few active CACTA elements were identified in sunflower [20,21,23].

Starting from our knowledge of the *Tetu1* element, we performed a genome-wide analysis of CACTA elements into the sunflower reference genome, i.e., the HanXRQ inbred line [24], to estimate their abundance, gene proximity, and related expression patterns.

## 2. Results

### 2.1. Identification of Putative CACTA Transposon Sequences in the Sunflower Genome

A genome-wide discovery of CACTA TEs was performed on the available sunflower reference genome sequence, exploiting the complete CACTA elements characterized by Badouin et al. [24] and *Tetu1* [20] as queries.

We identified 707 new putative CACTA sequences: 84 elements with complete ends (i.e., elements in which both TIRs present an intact CACTA motif) and 623 truncated elements (an annotation reporting the coordinates and features of the isolated elements can be found in Supplementary Material 1). The isolated transposons covered 13,649,233 out of 3,027,963,057 nt, corresponding to 0.45% of the reference genome of sunflower. Two CACTA sequences, retrieved on linkage groups 7 and 15 of the reference genome, showed 99.83% and 98.74% identity compared with *Tetu1*, respectively (Supplementary Material 2). These sequences likely represent two copies of *Tetu1* in the sunflower’s reference genome.

### 2.2. Classification of CACTA Transposons Based on their TR Sequences and Abundance Estimation

**Figure 2:**
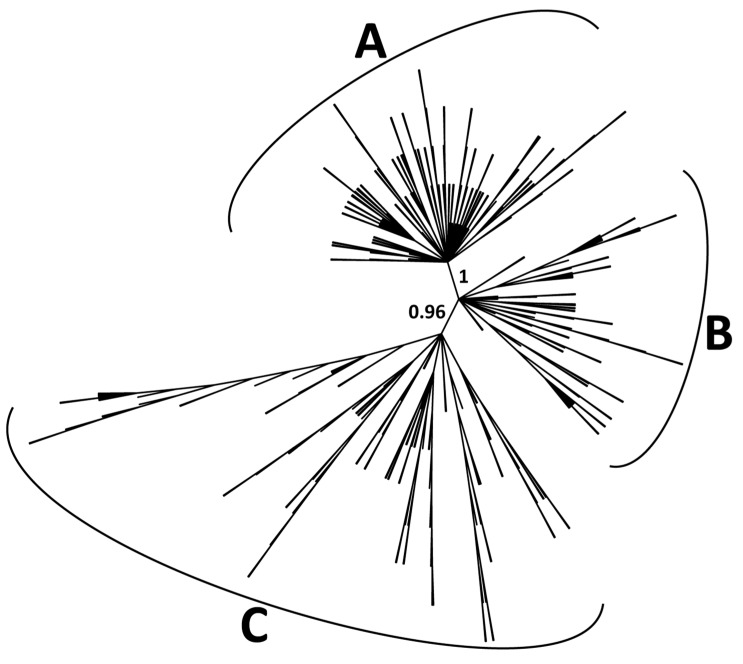
Phylogenetic tree of CACTA elements based on the TR sequences (347 sequences clustered). Bootstrap values higher than 0.9 are shown for basal nodes.

As the majority of the identified CACTA transposons have no coding domains and vary considerably in size, the classification of the elements was based on the TR sequences according to Wicker et al. [19]. A multi-sequence alignment was performed with the terminal 300 bp of all the collected elements and, after improving the alignment area by removing sequences too divergent, a maximum likelihood phylogenetic analysis allowed for the classification of 347 TR sequences into three main distinct clades (Figure 2). The high variability of the sequences analyzed could allow the identification of several small clades; for clarity, we limited the subsequent analyses to the three clades resulting from basal significant separation. The NEWICK file, related to maximum likelihood analysis, can be found in Supplementary Material 3. An additional phylogenetic tree was constructed using iqTree (http://www.iqtree.org/) in order to double-check the classification of CACTAs; the result (Supplementary Material 4) is consistent with our classification.

**Figure 3:**
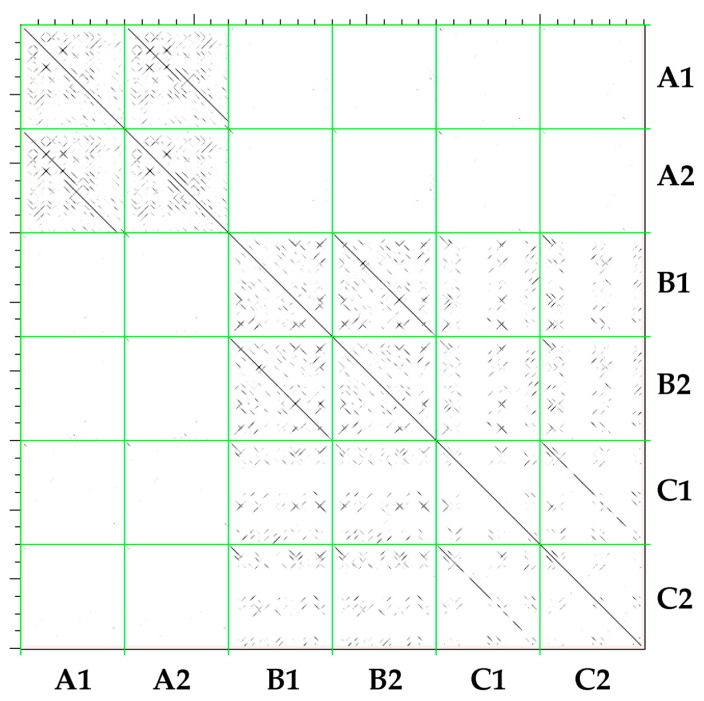
Matrix reporting the dot-plots for each pairwise combination of the 6 TR sequences (2 sequences from A, B, and C family, respectively).

This classification was tested by a second approach based on the similarity of TR sequences among the three different families, and displayed by an all vs. all dot-plot analysis. In Figure 3, the comparison of TRs from model members of the same family (e.g., A1 vs. A2) display a characteristic pattern called transposon signature, whereas TRs of elements belonging to different families show no signature. This is especially the case for members of the A family, which show a distinctive TR pattern.

In Table 1, the number of CACTA transposons, grouped by family, is reported. The major family, designated as A, contained 141 CACTA sequences, including the two identified copies of *Tetu1* (the only two complete elements of the A family), none of which had apparent coding capacity. Two additional families, families B and C, resulted in 84 and 122 elements, respectively. We also report, for each family, a sequence conservation value, defined as the minimum percentage of identity resulting from the corresponding multi-sequence alignment.

To gain insight into the repetitiveness of CACTA TEs within the sunflower genome, we evaluated the abundance of each family by mapping Illumina reads with two different strategies and counting the per base average coverage and copy numbers of each element.

The first mapping method was performed using stringent mapping parameters, and then, the mapping was repeated with relaxed parameters to avoid target read loss. In Table S1, a comparison between the two mapping methods is reported. For the second approach, 95,526 reads out of 68,949,014 were mapped onto the 707 putative CACTA elements. Overall, the three families showed a comparable level of per base average coverage (Table 2). Due to their low conservation level, the majority of CACTAs identified remained unclassified and constituted the group showing the highest per base average coverage and the average number of copies.

### 2.3. Proximity of CACTA Transposons to Genes and Functional Analysis

**Figure 4:**
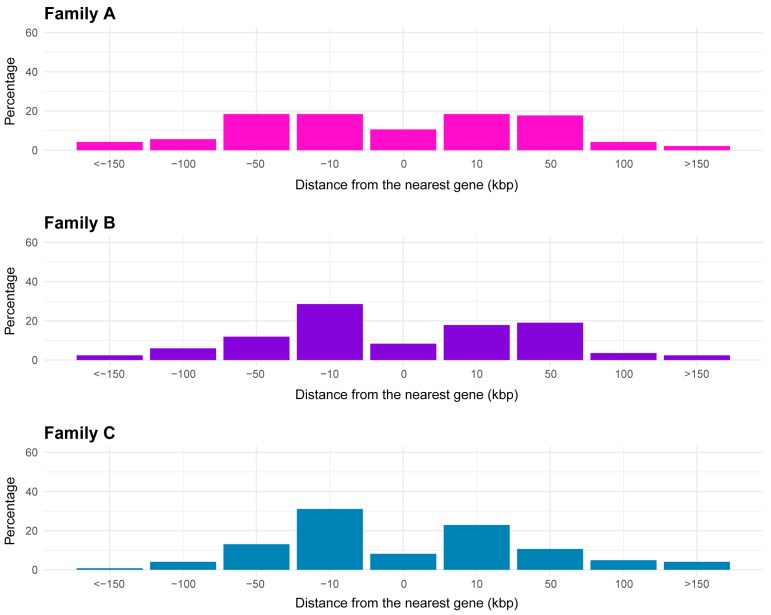
Bar plots reporting the proximity of CACTA TEs to the closest sunflower gene. Results are reported for families A, B, and C. Proximity to the closest gene is expressed in kilo base pairs (kbp).

To evaluate the potential impact of CACTA insertions on gene function, we analyzed the association between sequences belonging to the three families identified and the protein-encoding genes in the sunflower genome. On average, the majority of analyzed CACTAs lay close to genes, located between 1 and 50,000 bp upstream or downstream from the nearest gene (Figure 4). About 10% of the CACTAs of each identified family lay within a gene sequence.

**Figure 5:**
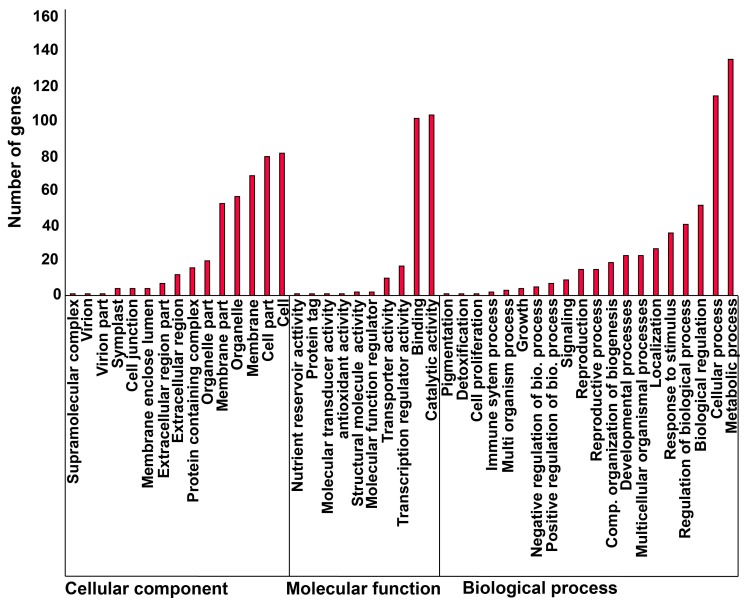
Gene ontology (GO) distribution for genes in close proximity to elements of different CACTA families. GO terms were subdivided into three major ontology classes: cellular component, molecular function, and biological process.

To gain biological insight into the phenotypic traits of sunflower that could be affected by the presence of CACTA elements in the proximity of genes, we performed a functional analysis of genes close to CACTAs. Gene ontology (GO) distribution per gene showed that the most frequent terms are metabolic process (GO:0008152) and cellular process (GO:0009987) in the biological process class, whereas catalytic activity (GO:0003824) and binding (GO:0005488) were retrieved for molecular function, and for cellular component, cell (GO:0005623) and cell part (GO:0044464) are the most distributed terms (Figure 5).

**Figure 6:**
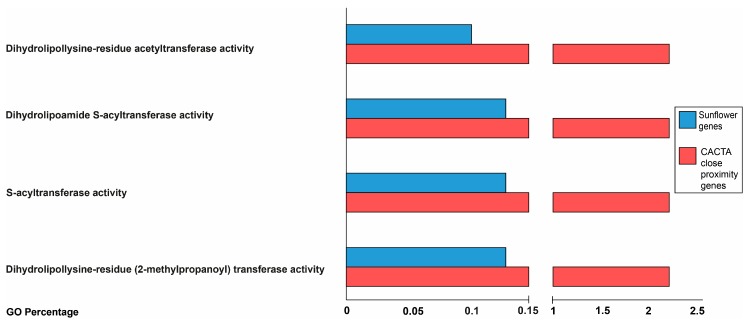
Enriched gene ontology (GO) terms distribution. GO terms of genes in close proximity to elements of CACTA family A (red bars) compared to GO terms of the whole gene set of sunflower (blue bars).

Finally, concerning enrichment analysis, Fisher’s exact test was performed on the GO terms of genes in close proximity to each CACTA family compared with the GO distribution of the other known genes of sunflower. Only CACTA family A showed four enriched terms: dihydrolipollysine-residue acetyltransferase activity (GO:0004742), dihydrolipoamide S-acyltransferase activity (GO:0030523), S-acyltransferase activity (GO:0016417), dihydrolipollysine-residue (2-methylpropanoyl) transferase activity (GO:0043754). The GO terms percentage of enrichment analysis are shown in Figure 6. Similarly, GO enrichment analysis was performed for genes upstream/downstream and for genes with CACTA regions residing within. Overall, we detected six and four GO terms enriched in genes upstream and with CACTAs residing within, respectively. Concerning enrichment analysis of genes downstream to CACTA elements, no significant results were found (Table S2).

### 2.4. Expression Analysis of CACTA Transposons and Genes Closest to CACTA Transposons in the Sunflower Genome

The expression of CACTA TEs in the leaves of sunflower was obtained by mapping Illumina cDNA reads onto a collection of 39 TPase-encoding sequences belonging to TEs of CACTA families B and C. Our analyses showed that the expression of CACTA TEs is extremely low, with only 10 TPase sequences showing a slight signal of activation, except for one element (CACTA 224) of family C (Table 3).

**Figure 7:**
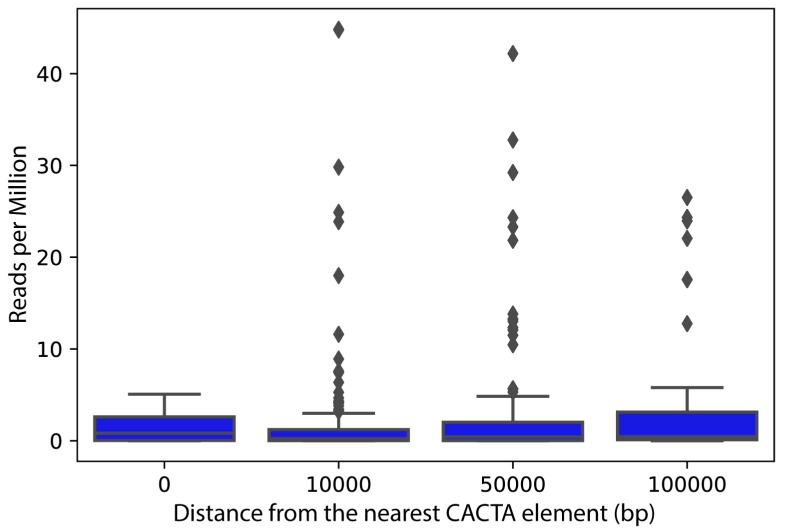
Expression (in mapped reads per million) of sunflower genes according to the distance from CACTA elements.

Finally, the expression of sunflower genes residing in proximity to CACTA elements was analyzed. Linear regression revealed a weak but significant (coefficient: 0.09, *p*-value < 0.01) effect of the distance from these elements on the expression of sunflower genes, i.e., genes close or colocalized with CACTA elements had lower expression than genes farther to CACTA elements (Figure 7).

## 3. Discussion

The previous decades have been marked by an exponential increase in genomic data, which have facilitated, among many other studies, the identification and characterization of repeated sequences. However, CACTA elements have remained poorly characterized for a long time. Despite being the most abundant DNA transposons (accounting for 10% of some grass genomes [19,27,28,29]), the identification and characterization of these TEs is difficult due to their low sequence conservation, which is mostly limited to the TR regions, and also because the majority of CACTA TEs are deletion derivatives not encoding a TPase [19].

Exploiting the genomic resources that are now available for sunflower, we performed a genome-wide analysis of CACTA elements in the sunflower genome to gain insight into the abundance, gene proximity, and related expression patterns of this superfamily, which previous studies have shown impact phenotypic variation. For example, in the Asteraceae, the members of CYCLOIDEA (CYC) 2 clade of TCP transcription factors are essential to control flower symmetry and are also crucial for the inflorescence (capitulum) architecture [22,30]. The ligulate-like inflorescence showed by the *Chrysanthemoides* (*Chry*) mutant of sunflower is the consequence of a small CACTA (1034 bp) inserted 558 bp before the initiation codon of the *HaCYC2c* gene [23,31]. This CACTA alters the transcriptional activity of *HaCYC2c*, in which the expression extends into the inflorescence, suggesting that the insertion of the TE is an essential step to generate the *Chry* phenotype. In contrast, when the basal region of the bHLH TCP motif of the *HaCYC2c* gene is interrupted by the insertion of the incomplete CACTA transposon *Tetu1****,*** the ray flowers are transformed from zygomorphic to actinomorphic, assuming a resemblance to the disk flowers [20,21,32]. In this mutant, (*turf*) the ray flowers also recover their hermaphroditic features, developing both male and female reproductive organs.

Based on our knowledge of the *Tetu1* sequence and exploiting the complete CACTA elements characterized by Badouin et al. [24], we identified 707 new putative CACTA sequences corresponding to 0.45% of the sunflower genome. Usually, CACTA elements are not considered to explain the large genome sizes found in plants. However, CACTA families can be highly abundant; for instance, *Tpo1* in *Lolium perenne* and *Caspar* in Triticeae have contributed to the expansion of the genome size of their host [19,33,34].

Concerning the CACTA elements, we were able to subdivide 347 sequences into three families based on the TR sequence conservation (Figure 2). The majority of the identified TEs were found to be non-autonomous, lacking the coding portion. For instance, family A consists of a group of non-autonomous elements sharing a sufficient level of similarity to be considered a clade. Evidence proved that some non-autonomous elements can be cross activated by autonomous partners belonging to different families [19]. This seems to be the case of *Tetu1*, which, despite being a non-autonomous element and also being similar to two elements of the A family, appears to be mobile due to the action of other TPases [32]. We isolated 39 TPase-encoding sequences from our database of sunflower CACTA elements, 10 of which show a weak signal of activation, and only one was found to be expressed more.

On average, the majority of the analyzed CACTAs found to be in close proximity to sunflower genes are located between 1 and 50,000 bp from the closest gene. TEs may sometimes be associated with regulatory elements of genes, thus, possibly influencing gene expression [23,31,35,36,37]. There is a notable case of a TE inserted 65 kb upstream of the *teosinte branched1* (*tb1*) gene of maize, which acts as an enhancer of gene expression [38]. However, a relevant fraction of elements of the identified CACTA families is found within a gene coding sequence. Functional analysis showed that most distributed GO terms of genes in close proximity to CACTA elements belong to broad biological classes (i.e., metabolic process, cellular processes, and cell part), so the insertion of CACTA may have resulted in the modification of major biological processes during the evolution of sunflower, or subsequently to an activation that might have changed the expression pattern of genes that have important biological functions. As shown by enrichment analysis, a significant part of the genes in proximity to CACTA elements are involved in acyltransferase activity (Figure 6). CACTA elements were shown to modify the expression of acyltransferases encoding transcripts, such as *chalcone-synthase* (*chs*) of *A. majus* [35]. In the unstable *nivea* locus of this species, an autonomous CACTA element *Tam1* was found inserted 17 bp upstream of the *chs* TATA box, a gene encoding for a key enzyme of the flavonoid/isoflavonoid biosynthesis pathway. The characteristic variegated phenotype of the snapdragon flowers originates from the somatic excision of *Tam1*.

Finally, the expression analysis of genes in close proximity to CACTAs revealed that most expressed genes lay in the range of 50,000 bp up or downstream of the sequence, whereas genes with a lower rate of expression hold CACTA within the sequence (Figure 7). Therefore, this analysis might suggest that the insertion of a CACTA within a gene could lead to the inactivation of host gene expression.

## 4. Material and Methods

### 4.1. Sequence Collection

Putative CACTA elements were isolated from the HanXRQr1.0 version of the sunflower genome sequence [24], deposited at the NCBI site (https://www.ncbi.nlm.nih.gov/) (WGS project number PRJNA396063). Putative CACTA sequences were isolated by using full-length elements retrieved by Badouin et al. [24] along with the sequence of *Tetu1* [20,21] as a query for a BLASTN search (-E-value 1e-10). This approach was found to be adequate for this type of particularly heterogeneous sequence. Collected elements were subsequently filtered for the presence of TR sequences distinguishing elements with complete ends, in which both TIRs present an intact CACTA motif, and truncated elements. The collection of putative CACTA elements was then used to mask the reference genome using RepeatMasker v4.0.3 [39] to obtain a more precise localization and coverage estimation. Masking results were analyzed using the Bedtools v2.27.0 [40] merge function and were then manually adjusted.

### 4.2. Abundance Estimation and DNA Mapping Procedure

For each CACTA sequence, the genomic abundance was first assessed by mapping DNA reads of the sunflower genome, downloaded from NCBI (SRR5004633), according to the strategy already used for repetitive sequences in Mascagni et al. [41,42]. Illumina HiSeq 2000 reads were preprocessed to remove Illumina adapters, then quality-trimmed using the default settings, and the lengths of reads were defined at 90 nt. The mapping procedure was tested using two different pipelines: it was first performed using CLC Genomics Workbench 9.5.3 (CLC-BIO, Aarhus, Denmark), with stringent parameters (length fraction = 0.9, similarity fraction = 0.9, mismatch penalty = 1, and gap penalty = 1). Afterward, BWA MEM version 0.7.13-r1126 [43] was used without enforcing a mapping quality cutoff (MAPQ ≥ 0). Then, Bedtools v. 2.27.0 was used to compute the average per-base coverage.

To compare the genomic redundancy of CACTA elements, seven single-copy genes previously published and wet validated [26,44] were used. The seven selected genes encode a dehydrin (FR670619.1), a heat shock protein (LOC110868885), a ζ-carotene desaturase (FR671183.1), a drought-responsive-element-binding protein (LOC110872740), a NAC-domain transcription regulator (FR671350.1), an auxin-binding protein (FR671175.1), and an ABA-responsive C5 protein (FR671167.1). The mapping was performed by using the same Illumina sequence read set as before. The analysis allowed us to estimate the CACTA copy numbers as the ratio between the per-base coverage of the CACTA elements and the coverage of single-copy regions.

### 4.3. Evolutionary Analysis using the Maximum Likelihood Method

A multiple sequence alignment of the terminal repeats (300 bp) of the identified putative CACTA sequences was performed using MAFFT version 7 [45]. To increase the number of gap-free sites, the MaxAlign 1.1 Server [46] was used, removing any sequences with too many gaps in the post-process stage of alignments, improving the alignment area. The evolutionary history of CACTAs was inferred by using the maximum likelihood method based on the Tamura–Nei model [47]. The bootstrap consensus tree was inferred from 500 replicates [48]. Analyses were conducted by using MEGA X [49]. Two TRs were selected for each family and used to perform a dot-plot analysis with DOTTER [50] for each pairwise combination. Sequence conservation was computed performing a multi-sequence alignment for each family with ClustalX [51] and considering the minimum percentage of identity value.

### 4.4. Analysis of Proximity of CACTA Elements to Genes

To estimate the distance and the sequence of the closest gene to each CACTA element, the coordinates of the CACTA elements were compared to those of the known genes of sunflower [24] by using Bedtools. Then, the corresponding gene sequences were retrieved from the HanXRQ genome annotation database (https://www.heliagene.org/HanXRQ-SUNRISE/). Gene ontology (GO) terms for each gene were extracted from the available b2g annotation file provided by the Heliagene website (https://www.heliagene.org/). Subsequently, GOs were grouped into three principal classes (molecular function, cellular component, and biological processes) and analyzed with Fisher’s exact test using Blast2GO [52]. Enrichment analysis was performed between GO terms of genes proximal to CACTAs and the whole set of sunflower genes; GOs were considered significantly enriched for an FDR-corrected *p*-value < 0.05. The effect of CACTA proximity on gene expression was tested with a linear regression.

### 4.5. TPase Domain Identification and CACTA Transposon Expression Analysis

Isolated CACTAs were screened for the occurrence of the TPase domain within their sequence, by using the domain-based annotation of transposable elements tool (DANTE, [4]). DANTE accomplishes domain searching by comparing the LASTAL alignment tool results against a database of Viridiplantae protein domains derived from TEs.

The expression of TPase domains and that of genes in close proximity to CACTAs were analyzed using Illumina cDNA paired-end libraries publicly available at the NCBI SRA (https://www.ncbi.nlm.nih.gov/sra/, accession number SRP092742) [24]. Such libraries were obtained from the leaves of sunflower plants grown under hydroponic conditions, as described by Badouin et al. [24].

High-quality paired-end reads of 45 nucleotide lengths were mapped onto isolated nucleotide sequences of the protein domain by using CLC Genomics Workbench (version 9.5.3, CLC-BIO, Aarhus, Denmark). The parameters used for the TPase domain were: similarity fraction = 0.9, length fraction = 0.9, mismatch penalty = 1, and gap open penalty = 1. The parameters used for gene mapping were the same, except for mismatch and gap penalties (mismatch penalty = 2 and gap open penalty = 3). The raw number of mapped reads for each TPase and gene sequences was normalized by calculating the number of mapped reads per million reads that were used for mapping (MRxM).

## 5. Conclusions

Although, in recent years, the role of repeated elements has been largely reconsidered, CACTA elements have remained poorly studied. Our study represents the first genome-wide analysis of such elements for sunflower, a model species for studying genome evolution, known for its huge repetitive component. A number of elements were fully characterized, identifying three clades of sunflower-specific CACTAs that include complete and incomplete elements among which we found two copies of *Tetu1*.

The majority of CACTA elements were found to be in close proximity to the nearest sunflower gene, whereas another relevant fraction is located within gene-encoding sequences with an impact on the expression of those genes.

Finally, we found that some CACTA elements are sporadically transcribed at a low rate in sunflower leaves, except for one element expressed at a high level. These elements are potential candidates for further studies to ascertain the occurrence of new insertions of CACTA elements in the sunflower genome that could be used for a transposon-tagging system similar to those based on *En/Spm* and *Ac/Ds* elements.

## Figures and Tables

**Table 1:** Description of the three CACTA families identified in the sunflower genome.

FAMILY	NO. OF ELEMENTS	COMPLETE ELEMENTS	TRUNCATED ELEMENTS	TPASE DOMAIN	SEQUENCE CONSERVATION
**A**	141	2	139	-	64
**B**	84	13	71	27	52
**C**	122	19	103	12	45
**TOTAL**	347	34	313	39	

**Table 2:** Mapping results of the sunflower nuclear genomic reads onto the total collection of putative CACTA elements.

FAMILY	FAMILY PER BASE AVERAGE COVERAGE	AVERAGE NUMBER OF COPIES
**A**	2.50	1.29
**B**	2.66	1.38
**C**	2.72	1.41
**UNKNOWN**	7.12	3.68

**Table 3:** Expressed transposase (TPase) domains of CACTA elements. The expression is reported in mapped reads per million, and for each element, the family is reported.

FAMILY	CACTA ELEMENT NAME	MAPPED READS PER MILLION
B	CACTA 641	0.0127
B	CACTA 506	0.0141
B	CACTA 314	0.0150
B	CACTA 203	0.0168
B	CACTA 292	0.0282
B	CACTA 610	0.0430
C	CACTA 492	0.008
C	CACTA 585	0.008
C	CACTA 242	0.023
C	CACTA 224	0.949

## Supplementary Materials

The following are available online at https://www.mdpi.com/1422-0067/21/6/2021/s1.

## Abbreviations

*Chry*	*Chrysanthemoides*
*CYC*	*CYCLOIDEA* gene
GO	Gene ontology
LTR	Long terminal repeats
REs	Retrotransposons
*TCP*	*TEOSINTE BRANCHED1/CYCLOIDEA/PROLIFERATING NUCLEAR ANTIGEN CELL FACTOR*
TEs	Transposable elements
*Tetu1*	*Transposable element of turf1*
TIRs	Terminal inverted repeats
TRs	Sub-terminal repeats
TPase	Transposase domain
TSD	Target-site duplication motif
*turf*	*Tubular ray flower*
