# Supplementary material for: On the Trail of Tetu1: Genome-Wide Discovery of CACTA Transposable Elements in Sunflower Genome
Source: Int J Mol Sci. 2020 Mar 16;21(6):2021. doi: 10.3390/ijms21062021 (PMC7139988; doi:10.3390/ijms21062021)
Supplement: Supplementary file 1 [file ijms-21-02021-s001.zip › ijms-748759-SI/Supplementary_Material_Ventimiglia_et_al/Supplementary_Material_4.pdf]

Supplementary material 4

The classification of the CACTA elements identified was verified using another phylogenetic analysis tool (iqTree http://www.iqtree.org/) using default parameters.

IQ-TREE 1.6.11 built Jun 6 2019

Input file name: 347.fa
Type of analysis: ModelFinder + tree reconstruction + ultrafast bootstrap (1000 replicates)
Random seed number: 627311

REFERENCES

To cite ModelFinder please use:

Subha Kalyaanamoorthy, Bui Quang Minh, Thomas KF Wong, Arndt von Haeseler,
and Lars S Jermiin (2017) ModelFinder: Fast model selection for
accurate phylogenetic estimates. Nature Methods, 14:587–589.
https://doi.org/10.1038/nmeth.4285

To cite IQ-TREE please use:

Lam-Tung Nguyen, Heiko A. Schmidt, Arndt von Haeseler, and Bui Quang Minh
(2015) IQ-TREE: A fast and effective stochastic algorithm for estimating
maximum likelihood phylogenies. Mol Biol Evol, 32:268-274.
https://doi.org/10.1093/molbev/msu300

Since you used ultrafast bootstrap (UFBoot) please also cite:

Diep Thi Hoang, Olga Chernomor, Arndt von Haeseler, Bui Quang Minh,
and Le Sy Vinh (2017) UFBoot2: Improving the ultrafast bootstrap
approximation. Mol Biol Evol, in press.
https://doi.org/10.1093/molbev/msx281

SEQUENCE ALIGNMENT

Input data: 347 sequences with 779 nucleotide sites
Number of constant sites: 217 (= 27.8562% of all sites)
Number of invariant (constant or ambiguous constant) sites: 217 (= 27.8562% of all sites)
Number of parsimony informative sites: 501
Number of distinct site patterns: 641

ModelFinder

Best-fit model according to BIC: TVM+F+I+G4

List of models sorted by BIC scores:

Model	LogL	AIC	w-AIC	AICc	w-AICc	BIC	w-BIC
TVM+F+I+G4	-24007.5878	49411.1756	+ 0.7238	61608.7256	- 0.0000	52662.4673	+ 0.9611
GTR+F+I+G4	-24007.5511	49413.1021	+ 0.2762	61800.4439	- 0.0000	52669.0518	- 0.0357
K3Pu+F+I+G4	-24020.3164	49432.6328	- 0.0000	61264.6328	- 0.0000	52674.6085	- 0.0022
TPM3+F+I+G4	-24021.9289	49435.8577	- 0.0000	61267.8577	- 0.0000	52677.8334	- 0.0004
TPM3u+F+I+G4	-24021.9290	49435.8580	- 0.0000	61267.8580	- 0.0000	52677.8337	- 0.0004
TIM+F+I+G4	-24020.2996	49434.5993	- 0.0000	61447.0931	- 0.0000	52681.2330	- 0.0001
TIM3+F+I+G4	-24021.8559	49437.7119	- 0.0000	61450.2057	- 0.0000	52684.3456	- 0.0000
TVM+F+G4	-24021.8742	49437.7483	- 0.0000	61450.2422	- 0.0000	52684.3820	- 0.0000
GTR+F+G4	-24021.7131	49439.4262	- 0.0000	61636.9762	- 0.0000	52690.7179	- 0.0000
TPM2u+F+I+G4	-24031.2879	49454.5757	- 0.0000	61286.5757	- 0.0000	52696.5514	- 0.0000
TPM2+F+I+G4	-24031.2912	49454.5824	- 0.0000	61286.5824	- 0.0000	52696.5581	- 0.0000
K3Pu+F+G4	-24035.0534	49460.1068	- 0.0000	61116.0105	- 0.0000	52697.4245	- 0.0000
HKY+F+I+G4	-24035.5816	49461.1632	- 0.0000	61117.0668	- 0.0000	52698.4809	- 0.0000
TPM3u+F+G4	-24036.1410	49462.2820	- 0.0000	61118.1857	- 0.0000	52699.5997	- 0.0000
TPM3+F+G4	-24036.1411	49462.2821	- 0.0000	61118.1858	- 0.0000	52699.5998	- 0.0000
TIM2+F+I+G4	-24031.2245	49456.4489	- 0.0000	61468.9428	- 0.0000	52703.0826	- 0.0000
TIM+F+G4	-24034.9560	49461.9119	- 0.0000	61293.9119	- 0.0000	52703.8876	- 0.0000
TN+F+I+G4	-24035.5681	49463.1362	- 0.0000	61295.1362	- 0.0000	52705.1119	- 0.0000
TIM3+F+G4	-24035.9287	49463.8574	- 0.0000	61295.8574	- 0.0000	52705.8331	- 0.0000
TPM2u+F+G4	-24046.2579	49482.5157	- 0.0000	61138.4193	- 0.0000	52719.8334	- 0.0000
TPM2+F+G4	-24046.2589	49482.5177	- 0.0000	61138.4214	- 0.0000	52719.8354	- 0.0000
HKY+F+G4	-24050.4414	49488.8829	- 0.0000	60972.9305	- 0.0000	52721.5425	- 0.0000
TIM2+F+G4	-24046.1967	49484.3934	- 0.0000	61316.3934	- 0.0000	52726.3691	- 0.0000

TN+F+G4	-24050.3544	49490.7087 - 0.0000	61146.6124 - 0.0000	52728.0264 - 0.0000
TVMe+I+G4	-24106.5828	49603.1657 - 0.0000	61259.0693 - 0.0000	52840.4834 - 0.0000
SYM+I+G4	-24105.5405	49603.0810 - 0.0000	61435.0810 - 0.0000	52845.0567 - 0.0000
TVMe+G4	-24119.8366	49627.6733 - 0.0000	61111.7209 - 0.0000	52860.3329 - 0.0000
SYM+G4	-24119.1282	49628.2563 - 0.0000	61284.1599 - 0.0000	52865.5740 - 0.0000
TIM3e+I+G4	-24148.9243	49685.8486 - 0.0000	61169.8962 - 0.0000	52918.5082 - 0.0000
TIM2e+I+G4	-24153.3377	49694.6755 - 0.0000	61178.7231 - 0.0000	52927.3351 - 0.0000
TIM3e+G4	-24162.4044	49710.8087 - 0.0000	61027.0911 - 0.0000	52938.8104 - 0.0000
TIM2e+G4	-24168.0232	49722.0463 - 0.0000	61038.3287 - 0.0000	52950.0480 - 0.0000
K3P+I+G4	-24168.2603	49722.5205 - 0.0000	61038.8029 - 0.0000	52950.5222 - 0.0000
TIMe+I+G4	-24166.9281	49721.8562 - 0.0000	61205.9038 - 0.0000	52954.5159 - 0.0000
K3P+G4	-24182.4352	49748.8705 - 0.0000	60901.3356 - 0.0000	52972.2141 - 0.0000
TIMe+G4	-24181.4779	49748.9559 - 0.0000	61065.2382 - 0.0000	52976.9575 - 0.0000
K2P+I+G4	-24187.6445	49759.2890 - 0.0000	60911.7542 - 0.0000	52982.6327 - 0.0000
TNe+I+G4	-24186.3016	49758.6032 - 0.0000	61074.8856 - 0.0000	52986.6049 - 0.0000
K2P+G4	-24201.8953	49785.7906 - 0.0000	60778.2504 + 1.0000	53004.4762 - 0.0000
TNe+G4	-24200.9210	49785.8420 - 0.0000	60938.3071 - 0.0000	53009.1857 - 0.0000
F81+F+I+G4	-24453.2052	50294.4105 - 0.0000	61778.4581 - 0.0000	53527.0702 - 0.0000
F81+F+G4	-24467.7198	50321.4396 - 0.0000	61637.7220 - 0.0000	53549.4413 - 0.0000
JC+I+G4	-24592.9869	50567.9737 - 0.0000	61560.4335 - 0.0000	53786.6594 - 0.0000
JC+G4	-24607.6550	50595.3100 - 0.0000	61431.4463 - 0.0000	53809.3376 - 0.0000
K3Pu+F+I	-24645.1706	50680.3413 - 0.0000	62336.2449 - 0.0000	53917.6589 - 0.0000
TVM+F+I	-24639.1124	50672.2247 - 0.0000	62684.7186 - 0.0000	53918.8584 - 0.0000
TIM+F+I	-24645.1234	50682.2468 - 0.0000	62514.2468 - 0.0000	53924.2224 - 0.0000
GTR+F+I	-24639.0832	50674.1665 - 0.0000	62871.7165 - 0.0000	53925.4582 - 0.0000
TPM3u+F+I	-24649.2321	50688.4642 - 0.0000	62344.3678 - 0.0000	53925.7818 - 0.0000
TPM3+F+I	-24649.2321	50688.4642 - 0.0000	62344.3678 - 0.0000	53925.7819 - 0.0000
TIM3+F+I	-24649.1853	50690.3706 - 0.0000	62522.3706 - 0.0000	53932.3462 - 0.0000
HKY+F+I	-24658.4488	50704.8976 - 0.0000	62188.9452 - 0.0000	53937.5573 - 0.0000
TPM2u+F+I	-24657.7044	50705.4088 - 0.0000	62361.3124 - 0.0000	53942.7264 - 0.0000
TPM2+F+I	-24657.7054	50705.4107 - 0.0000	62361.3144 - 0.0000	53942.7284 - 0.0000
TN+F+I	-24658.3996	50706.7991 - 0.0000	62362.7028 - 0.0000	53944.1168 - 0.0000
TIM2+F+I	-24657.6563	50707.3126 - 0.0000	62539.3126 - 0.0000	53949.2883 - 0.0000
K3Pu+F	-24738.3373	50864.6746 - 0.0000	62348.7222 - 0.0000	54097.3343 - 0.0000
TVM+F	-24732.1141	50856.2281 - 0.0000	62688.2281 - 0.0000	54098.2038 - 0.0000
TIM+F	-24738.1381	50866.2762 - 0.0000	62522.1798 - 0.0000	54103.5939 - 0.0000
TPM3+F	-24741.6669	50871.3338 - 0.0000	62355.3815 - 0.0000	54103.9935 - 0.0000
TPM3u+F	-24741.6721	50871.3443 - 0.0000	62355.3919 - 0.0000	54104.0039 - 0.0000
GTR+F	-24731.9173	50857.8347 - 0.0000	62870.3285 - 0.0000	54104.4684 - 0.0000
TIM3+F	-24741.4750	50872.9501 - 0.0000	62528.8537 - 0.0000	54110.2677 - 0.0000
HKY+F	-24751.4078	50888.8155 - 0.0000	62205.0979 - 0.0000	54116.8172 - 0.0000
TPM2+F	-24751.2001	50890.4002 - 0.0000	62374.4478 - 0.0000	54123.0598 - 0.0000
TPM2u+F	-24751.2004	50890.4008 - 0.0000	62374.4484 - 0.0000	54123.0605 - 0.0000
TN+F	-24751.2047	50890.4094 - 0.0000	62374.4570 - 0.0000	54123.0690 - 0.0000

TIM2+F	-24750.9948	50891.9896 - 0.0000	62547.8933 - 0.0000	54129.3073 - 0.0000
TVMe+I	-24776.7938	50941.5877 - 0.0000	62425.6353 - 0.0000	54174.2473 - 0.0000
SYM+I	-24775.3854	50940.7709 - 0.0000	62596.6745 - 0.0000	54178.0886 - 0.0000
TIM3e+I	-24801.3170	50988.6339 - 0.0000	62304.9163 - 0.0000	54216.6356 - 0.0000
K3P+I	-24807.7410	50999.4821 - 0.0000	62151.9472 - 0.0000	54222.8257 - 0.0000
TIMe+I	-24806.3703	50998.7407 - 0.0000	62315.0230 - 0.0000	54226.7423 - 0.0000
TIM2e+I	-24809.8684	51005.7369 - 0.0000	62322.0192 - 0.0000	54233.7385 - 0.0000
K2P+I	-24826.3340	51034.6680 - 0.0000	62027.1277 - 0.0000	54253.3536 - 0.0000
TNe+I	-24824.9460	51033.8920 - 0.0000	62186.3572 - 0.0000	54257.2357 - 0.0000
TVMe	-24875.2561	51136.5122 - 0.0000	62452.7945 - 0.0000	54364.5138 - 0.0000
SYM	-24874.1798	51136.3595 - 0.0000	62620.4071 - 0.0000	54369.0192 - 0.0000
TIM3e	-24897.4236	51178.8471 - 0.0000	62331.3123 - 0.0000	54402.1908 - 0.0000
K3P	-24904.2001	51190.4002 - 0.0000	62182.8600 - 0.0000	54409.0858 - 0.0000
TIMe	-24903.1542	51190.3084 - 0.0000	62342.7735 - 0.0000	54413.6521 - 0.0000
TIM2e	-24909.4271	51202.8542 - 0.0000	62355.3194 - 0.0000	54426.1979 - 0.0000
K2P	-24922.8810	51225.7620 - 0.0000	62061.8984 - 0.0000	54439.7896 - 0.0000
TNe	-24921.7500	51225.5000 - 0.0000	62217.9598 - 0.0000	54444.1857 - 0.0000
F81+F+I	-25069.6446	51525.2892 - 0.0000	62841.5716 - 0.0000	54753.2909 - 0.0000
F81+F	-25162.9605	51709.9210 - 0.0000	62862.3861 - 0.0000	54933.2646 - 0.0000
JC+I	-25217.5610	51815.1220 - 0.0000	62651.2583 - 0.0000	55029.1496 - 0.0000
JC	-25312.9951	52003.9903 - 0.0000	62687.3611 - 0.0000	55213.3599 - 0.0000

AIC, w-AIC : Akaike information criterion scores and weights.
AICc, w-AICc : Corrected AIC scores and weights.
BIC, w-BIC : Bayesian information criterion scores and weights.

Plus signs denote the 95% confidence sets.
Minus signs denote significant exclusion.

SUBSTITUTION PROCESS

Model of substitution: TVM+F+I+G4

Rate parameter R:

A-C: 0.8730
A-G: 2.7783
A-T: 1.4067
C-G: 0.9892
C-T: 2.7783
G-T: 1.0000

State frequencies: (empirical counts from alignment)

pi(A) = 0.2826
pi(C) = 0.2297
pi(G) = 0.2022
pi(T) = 0.2856

Rate matrix Q:

A	-0.9466	0.1631	0.4568	0.3267
C	0.2006	-1.009	0.1626	0.6452
G	0.6385	0.1848	-1.056	0.2322
T	0.3233	0.519	0.1644	-1.007

Model of rate heterogeneity: Invar+Gamma with 4 categories
Proportion of invariable sites: 0.01821
Gamma shape alpha: 1.604

Category	Relative_rate	Proportion
0	0	0.01821
1	0.2454	0.2454
2	0.6162	0.2454
3	1.076	0.2454
4	2.137	0.2454

Relative rates are computed as MEAN of the portion of the Gamma distribution falling in the category.

MAXIMUM LIKELIHOOD TREE

Log-likelihood of the tree: -23855.2556 (s.e. 873.4117)
Unconstrained log-likelihood (without tree): -4878.1706
Number of free parameters (#branches + #model parameters): 698
Akaike information criterion (AIC) score: 49106.5113
Corrected Akaike information criterion (AICc) score: 61304.0613
Bayesian information criterion (BIC) score: 52357.8030

Total tree length (sum of branch lengths): 25.3625
Sum of internal branch lengths: 12.4962 (49.2702% of tree length)

WARNING: 58 near-zero internal branches (<0.0013) should be treated with caution
Such branches are denoted by '**' in the figure below

NOTE: Tree is UNROOTED although outgroup taxon 'HanXRQChr11_78963679_78964101' is drawn at root

Numbers in parentheses are SH-aLRT support (%) / ultrafast bootstrap support (%)

```
+--HanXRQChr11_78963679_78964101
|
|       +--HanXRQChr01_34887426_34894282
|       +**| (0/92)
|       | | +**HanXRQChr12_24613340_24614343
|       | +**| (0/95)
|       |       +**HanXRQChr08_22937900_22939039
|       +--| (87.6/98)
|       | +--HanXRQChr13_45702842_45703840
| +**| (0/98)
| | +--HanXRQChr17_205330461_205331450
+--| (76.7/100)
| +--HanXRQChr03_26205754_26213308
|
|       +--HanXRQChr04_67540092_67541320
|       +--| (89.9/100)
|       | |     +--HanXRQChr06_27984911_27988051
|       | | +--| (90.5/100)
|       | | | | +--HanXRQChr04_12175865_12181403
|       | | +**| (0/55)
|       | | |   |       +--HanXRQChr17_211327788_211333859
|       | | |   |       +--| (90.6/100)
|       | | |   |       | | +**HanXRQChr05_59284493_59290538
|       | | |   |       | +--| (98.4/100)
|       | | |   |       |       +**HanXRQChr05_106376295_106382341
|       | | |   |       +**| (0/93)
|       | | |   |       | |     +--HanXRQChr09_48889131_48889670
|       | | |   |       | | +--| (83.6/99)
|       | | |   |       | | | +--HanXRQChr15_55516101_55518531
|       | | |   |       | +--| (98.8/99)
|       | | |   |       |       |               +--HanXRQChr03_57480187_57481416
|       | | |   |       |       |       +**| (0/80)
|       | | |   |       |       |       | | +--HanXRQChr05_166779247_166780763
|       | | |   |       |       |       | +--| (79.5/100)
|       | | |   |       |       |       |       +--HanXRQChr12_94875864_94876484
|       | | |   |       |       |       +--| (80.6/99)
|       | | |   |       |       |       | | +--HanXRQChr04_92418435_92419412
|       | | |   |       |       |       | +--| (100/100)
|       | | |   |       |       |       |       | +**HanXRQChr06_53262309_53263292
|       | | |   |       |       |       |       +--| (75.5/100)
|       | | |   |       |       |       |       +--HanXRQChr05_9034545_9035531
|       | | |   |       |       |       +--| (95.9/99)
```

```
|         | | |    |         |         |         |  +--HanXRQChr11_113608046_113608769
|         | | |    |         |         |         +--| (19.7/50)
|         | | |    |         |         |         | |       +--HanXRQChr16_94477431_94477942
|         | | |    |         |         |         | |       +--| (70.6/96)
|         | | |    |         |         |         | |       | |       +--HanXRQChr17_67579875_67582613
|         | | |    |         |         |         | |       | |       +--| (99.9/100)
|         | | |    |         |         |         | |       | |       |  +--
HanXRQChr11_142203384_142204256
|         | | |    |         |         |         | |       | |       +**| (0/88)
|         | | |    |         |         |         | |       | |       |  +--
HanXRQChr04_176555457_176557628
|         | | |    |         |         |         | |       | |  +--| (73.8/96)
|         | | |    |         |         |         | |       | | |  +--HanXRQChr01_116026086_116027124
|         | | |    |         |         |         | |       |  +--| (99.9/100)
|         | | |    |         |         |         | |       |         +--HanXRQChr13_137859189_137859594
|         | | |    |         |         |         | |       +--| (79/97)
|         | | |    |         |         |         | |       |  +--HanXRQChr10_152624866_152626709
|         | | |    |         |         |         | |  +--| (100/100)
|         | | |    |         |         |         | | | |            +--HanXRQChr07_57474618_57475394
|         | | |    |         |         |         | | | |  +--| (82.2/100)
|         | | |    |         |         |         | | | | |  +--HanXRQChr07_57434299_57438258
|         | | |    |         |         |         | | | |  +--| (93/100)
|         | | |    |         |         |         | | | | | |  +--HanXRQChr05_190757444_190758598
|         | | |    |         |         |         | | | | |  +--| (43/100)
|         | | |    |         |         |         | | | | |         |  +--HanXRQChr13_94278579_94280493
|         | | |    |         |         |         | | | | |         +--| (99.8/100)
|         | | |    |         |         |         | | | | |         +--HanXRQChr04_115355662_115358538
|         | | |    |         |         |         | | |  +--| (94.4/98)
|         | | |    |         |         |         | | |     |         +**HanXRQChr11_18867372_18868580
|         | | |    |         |         |         | | |     |         +**| (0/73)
|         | | |    |         |         |         | | |     |         |  +**HanXRQChr02_20281946_20283260
|         | | |    |         |         |         | | |     |  +--| (98.5/100)
|         | | |    |         |         |         | | |     | |  +--HanXRQChr11_18749900_18751215
|         | | |    |         |         |         | | |     +--| (79.9/98)
|         | | |    |         |         |         | | |     +--HanXRQChr06_28223014_28227854
|         | | |    |         |         |         |  +--| (91.2/94)
|         | | |    |         |         |         |         |         +----HanXRQChr12_88474680_88475105
|         | | |    |         |         |         |         |  +--| (88.6/99)
|         | | |    |         |         |         |         | | |              +--
HanXRQChr01_1207903_1208488
|         | | |    |         |         |         |         | | |              +--| (78/99)
|         | | |    |         |         |         |         | | |              | |  +--
HanXRQChr08_151986814_151987353
|         | | |    |         |         |         |         | | |              |  +--| (97.9/100)
```

```
|     | |   |    |    |    |    | | |       |    +--
HanXRQChr10_94811711_94812246
|    | | |   |    |    |    |    | | |       +--| (87.6/99)
|    | | |   |    |    |    |    | | |       | |  +--
HanXRQChr05_168861330_168861873
|    | | |   |    |    |    |    | | |       |  +--| (98.5/100)
|    | | |   |    |    |    |    | | |       |    |  +--
HanXRQChr05_193946694_193947239
|    | | |   |    |    |    |    | | |       |    +--| (89.2/100)
|    | | |   |    |    |    |    | | |       |    +--
HanXRQChr05_193733456_193734002
|    | | |   |    |    |    |    | | |   +--| (90/99)
|    | | |   |    |    |    |    | | |   |  +--
HanXRQChr05_182283860_182284319
|    | | |   |    |    |    |    | |  +-------| (100/100)
|    | | |   |    |    |    |    | |     |  +--
HanXRQChr17_105500849_105501312
|    | | |   |    |    |    |    | |     +--| (90.4/98)
|    | | |   |    |    |    |    | |           |    +--
HanXRQChr04_16618909_16619279
|    | | |   |    |    |    |    | |           |  +--| (62/60)
|    | | |   |    |    |    |    | |           | | |   +--
HanXRQChr01_146764857_146765256
|    | | |   |    |    |    |    | |           | |  +-------| (100/100)
|    | | |   |    |    |    |    | |           | |     |
+**HanXRQChr03_20364521_20364920
|    | | |   |    |    |    |    | |           | |     +**| (0/76)
|    | | |   |    |    |    |    | |           | |          |
+**HanXRQChr05_14305976_14306375
|    | | |   |    |    |    |    | |           | |          +**| (0/38)
|    | | |   |    |    |    |    | |           | |
     +**HanXRQChr16_185568115_185568603
|    | | |   |    |    |    |    | |           +--| (84.8/62)
|    | | |   |    |    |    |    | |           +--
HanXRQChr03_113315527_113315992
|    | | |   |    |    |    |    +--| (54.5/93)
|    | | |   |    |    |    |    |           +--
HanXRQChr02_175754451_175758707
|    | | |   |    |    |    |    |           +--| (95.9/93)
|    | | |   |    |    |    |    |           |  +--
HanXRQChr13_94161763_94165162
|    | | |   |    |    |    |    |     +--| (77.6/68)
|    | | |   |    |    |    |    |     | |  +--HanXRQChr11_38886892_38889248
|    | | |   |    |    |    |    |     |  +--| (93.2/93)
```

```
|       | | |   |       |       |       |       |       | +--
HanXRQChr05_164509744_164514326
|       | | |   |       |       |       |       |       +--| (40/82)
|       | | |   |       |       |       |       |       +--
HanXRQChr03_34772558_34773468
|       | | |   |       |       |       |       +--| (98.5/91)
|       | | |   |       |       |       |       | |     +--
HanXRQChr08_33398524_33406006
|       | | |   |       |       |       |       | |     +--| (95.1/100)
|       | | |   |       |       |       |       | |     | +--
HanXRQChr12_40586043_40589256
|       | | |   |       |       |       |       | |     +--| (38.4/85)
|       | | |   |       |       |       |       | |     | | +--
HanXRQChr13_77076783_77077636
|       | | |   |       |       |       |       | |     | +--| (98.4/100)
|       | | |   |       |       |       |       | |     |       |       +--
HanXRQChr10_115966867_115967362
|       | | |   |       |       |       |       | |     |       |       +**| (0/14)
|       | | |   |       |       |       |       | |     |       |       | +--
HanXRQChr17_164520103_164521773
|       | | |   |       |       |       |       | |     |       |       +**| (0/19)
|       | | |   |       |       |       |       | |     |       |       | | +--
HanXRQChr01_49628126_49635945
|       | | |   |       |       |       |       | |     |       |       | +**| (0/34)
|       | | |   |       |       |       |       | |     |       |       |       +--
HanXRQChr14_68245992_68247226
|       | | |   |       |       |       |       | |     |       | +--| (78.4/76)
|       | | |   |       |       |       |       | |     |       | | | +--
HanXRQChr09_125961449_125964480
|       | | |   |       |       |       |       | |     |       | | +--| (76.4/70)
|       | | |   |       |       |       |       | |     |       | |     |       +--
HanXRQChr06_28014629_28015962
|       | | |   |       |       |       |       | |     |       | |     | +--| (72.3/100)
|       | | |   |       |       |       |       | |     |       | |     | | +--
HanXRQChr11_23461540_23465141
|       | | |   |       |       |       |       | |     |       | |     +--| (77.7/76)
|       | | |   |       |       |       |       | |     |       | |     +--
HanXRQChr04_166379772_166382442
|       | | |   |       |       |       |       | |     |       +--| (80.2/99)
|       | | |   |       |       |       |       | |     |       |       +--
HanXRQChr04_174582120_174583103
|       | | |   |       |       |       |       | |     |       | +--| (87.6/99)
|       | | |   |       |       |       |       | |     |       | | +--
HanXRQChr13_135224556_135226019
```

```
|       | | |   |       |       |       |       | |     |       +--| (81.1/78)
|       | | |   |       |       |       |       | |     |       |  +--
HanXRQChr07_11945240_11945839
|       | | |   |       |       |       |       | |     |       +--| (86.5/100)
|       | | |   |       |       |       |       | |     |       +--
HanXRQChr05_23977914_23979210
|       | | |   |       |       |       |       | |  +--| (93.4/99)
|       | | |   |       |       |       |       | | |  +--
HanXRQChr03_50693386_50694309
|       | | |   |       |       |       |       |  +--| (84/98)
|       | | |   |       |       |       |       |       +--
HanXRQChr08_40474325_40480066
|       | | |   |       |       |       |       +--| (68.7/85)
|       | | |   |       |       |       |       | |             +--
HanXRQChr02_24653810_24657437
|       | | |   |       |       |       |       | |             +--| (87.4/100)
|       | | |   |       |       |       |       | |             | |  +--
HanXRQChr14_68289895_68297518
|       | | |   |       |       |       |       | |             |  +--| (87/100)
|       | | |   |       |       |       |       | |             |       +--
HanXRQChr14_68273516_68281146
|       | | |   |       |       |       |       | |             +--| (85.8/90)
|       | | |   |       |       |       |       | |             | |
        +**HanXRQChr02_147295547_147297741
|       | | |   |       |       |       |       | |             | |             +--| (88.1/100)
|       | | |   |       |       |       |       | |             | |             |
+**HanXRQChr09_142942803_142944567
|       | | |   |       |       |       |       | |             | |     +--| (74.4/100)
|       | | |   |       |       |       |       | |             | |     |  +--
HanXRQChr13_96620660_96621156
|       | | |   |       |       |       |       | |             | |     +--| (89.6/100)
|       | | |   |       |       |       |       | |             | |     |  +--
HanXRQChr04_132271481_132272204
|       | | |   |       |       |       |       | |             | |     +--| (72.9/100)
|       | | |   |       |       |       |       | |             | |     |  +--
HanXRQChr14_153324342_153328229
|       | | |   |       |       |       |       | |             | |  +--| (74.5/100)
|       | | |   |       |       |       |       | |             | | |  +--
HanXRQChr10_85192350_85197513
|       | | |   |       |       |       |       | |             |  +--| (96.3/91)
|       | | |   |       |       |       |       | |             |       |       +--
HanXRQChr06_97006962_97010930
|       | | |   |       |       |       |       | |             |       |  +--| (84.7/100)
```

| | | | | | | | | | | | | +--
HanXRQChr03_159793635_159795071
| | | | | | | | | | | +--| (100/100)
| | | | | | | | | | | | +--
HanXRQChr07_88885423_88893193
| | | | | | | | | | | | +**| (0/34)
| | | | | | | | | | | | |
+**HanXRQChr08_39439464_39440150
| | | | | | | | | | | | +--| (98.8/100)
| | | | | | | | | | | | | +--
HanXRQChr08_109696851_109697593
| | | | | | | | | | | +--| (84.5/95)
| | | | | | | | | | | | +--
HanXRQChr01_140346486_140347133
| | | | | | | | | | | +--| (75.9/99)
| | | | | | | | | | | +--
HanXRQChr16_117883570_117885696
| | | | | | | | | | +--| (96.4/90)
| | | | | | | | | | | +--
HanXRQChr10_225800164_225801136
| | | | | | | | | | +--| (86.1/82)
| | | | | | | | | | | | +--
HanXRQChr04_79257170_79260978
| | | | | | | | | | | | +--| (97.2/100)
| | | | | | | | | | | | | | +--
HanXRQChr09_141496442_141499885
| | | | | | | | | | | | | +**| (0/85)
| | | | | | | | | | | | | +--
HanXRQChr03_70542853_70547885
| | | | | | | | | | | +--| (75/88)
| | | | | | | | | | | +--
HanXRQChr07_94262118_94263107
| | | | | | | | | | +--| (93.3/99)
| | | | | | | | | | | | +--
HanXRQChr17_170737481_170744938
| | | | | | | | | | | | +--| (28.7/88)
| | | | | | | | | | | | | +--
HanXRQChr04_115339901_115344808
| | | | | | | | | | | | +--| (82.6/100)
| | | | | | | | | | | | | +--
HanXRQChr05_177667685_177675472
| | | | | | | | | | | | +--| (96.1/100)
| | | | | | | | | | | | | |
+**HanXRQChr13_179578065_179585597

| | | | | | | | | | | | | | | +--| (96.1/100)
| | | | | | | | | | | | | | | |
+**HanXRQChr13_179805528_179808054
| | | | | | | | | | | | | | | +**| (0/56)
| | | | | | | | | | | | | | | | | +--
HanXRQChr05_64422906_64424659
| | | | | | | | | | | | | | | | +--| (77.5/99)
| | | | | | | | | | | | | | | | +--
HanXRQChr13_7315803_7323380
| | | | | | | | | | | | | | | +--| (81.3/61)
| | | | | | | | | | | | | | | | +--
HanXRQChr15_23213675_23214340
| | | | | | | | | | | | | | | +**| (0/46)
| | | | | | | | | | | | | | | | | +--
HanXRQChr11_127530325_127537865
| | | | | | | | | | | | | | | | +**| (0/67)
| | | | | | | | | | | | | | | | +--
HanXRQChr12_23644236_23645297
| | | | | | | | | | | | | | | +--| (92.4/92)
| | | | | | | | | | | | | | | | | +--
HanXRQChr14_116526279_116533821
| | | | | | | | | | | | | | | | +--| (22.1/75)
| | | | | | | | | | | | | | | | +--
HanXRQChr17_205323932_205330231
| | | | | | | | | | | | | | | +--| (93.6/90)
| | | | | | | | | | | | | | | | |
+**HanXRQChr17_3057459_3059571
| | | | | | | | | | | | | | | | +**| (0/88)
| | | | | | | | | | | | | | | | | +--
HanXRQChr13_169241697_169244853
| | | | | | | | | | | | | | | | | +**| (0/68)
| | | | | | | | | | | | | | | | | | +--
HanXRQChr01_90452614_90460077
| | | | | | | | | | | | | | | | +--| (79.3/100)
| | | | | | | | | | | | | | | | | +--
HanXRQChr13_45704069_45710378
| | | | | | | | | | | | | | | | +--| (71.6/100)
| | | | | | | | | | | | | | | |
+**HanXRQChr08_22931465_22937801
| | | | | | | | | | | | | | | +--| (89.5/100)
| | | | | | | | | | | | | | | | +--
HanXRQChr13_196274607_196275703
| | | | | | | | | | | | | | +--| (90.5/94)

| | | | | | | | | | | | | | | +--
HanXRQChr13_48436772_48438877
| | | | | | | | | | | | | | | +--| (85.6/89)
| | | | | | | | | | | | | | | | | | +--
HanXRQChr15_129312466_129317224
| | | | | | | | | | | | | | | | | | +**| (0/57)
| | | | | | | | | | | | | | | | | | | +--
HanXRQChr02_147809574_147817424
| | | | | | | | | | | | | | | | | | +--| (82/89)
| | | | | | | | | | | | | | | | | | | | | +--
HanXRQChr14_171031165_171032099
| | | | | | | | | | | | | | | | | | | +--| (99.9/100)
| | | | | | | | | | | | | | | | | | | +--
HanXRQChr10_113962499_113970363
| | | | | | | | | | | | | | | | | +**| (0/81)
| | | | | | | | | | | | | | | | | | +--
HanXRQChr11_23755532_23757425
| | | | | | | | | | | | | | | | | +**| (0/67)
| | | | | | | | | | | | | | | | | +--
HanXRQChr01_4643702_4650088
| | | | | | | | | | | | | | | +--| (85.6/97)
| | | | | | | | | | | | | | | +--
HanXRQChr08_88222000_88228288
| | | | | | | | | | | | | +--| (22.8/91)
| | | | | | | | | | | | | +--
HanXRQChr15_150283897_150291604
| | | | | | | | | | | | +--| (96.6/99)
| | | | | | | | | | | | | | +--
HanXRQChr03_46527653_46534444
| | | | | | | | | | | | | | +--| (66.4/100)
| | | | | | | | | | | | | | | +--
HanXRQChr06_51391635_51395795
| | | | | | | | | | | | | +--| (94.8/100)
| | | | | | | | | | | | | +--
HanXRQChr07_44435735_44436200
| | | | | | | | | | | | +--| (37.5/70)
| | | | | | | | | | | | | +--
HanXRQChr10_167884487_167885281
| | | | | | | | | | | +--| (43.7/75)
| | | | | | | | | | | | +--
HanXRQChr16_149753884_149754258
| | | | | | | | | | | | +--| (21.8/78)
| | | | | | | | | | | | | +--
HanXRQChr01_98528860_98530430

```
|         | | |    |         |         |         |         |         |  +--| (79.7/69)
|         | | |    |         |         |         |         |         | |  +--
HanXRQChr16_128066409_128067498
|         | | |    |         |         |         |         |         +--| (90.5/72)
|         | | |    |         |         |         |         |         |         +--
HanXRQChr02_20242486_20243525
|         | | |    |         |         |         |         |         |  +--| (83.2/100)
|         | | |    |         |         |         |         |         | |  +--
HanXRQChr11_18762949_18764264
|         | | |    |         |         |         |         |         +--| (86.2/100)
|         | | |    |         |         |         |         |         +--
HanXRQChr16_148694596_148701069
|         | | |    |         |         |         |         +--------| (100/100)
|         | | |    |         |         |         |                   |         +--
HanXRQChr15_23293519_23294345
|         | | |    |         |         |         |                   |         +--| (24.5/79)
|         | | |    |         |         |         |                   |         | |       +--
HanXRQChr06_29856663_29857319
|         | | |    |         |         |         |                   |         |  +-----| (99.2/100)
|         | | |    |         |         |         |                   |         |         +--------------
HanXRQChr17_60426850_60428287
|         | | |    |         |         |         |                   |  +--| (65.6/89)
|         | | |    |         |         |         |                   | | |
          +--HanXRQChr07_30137334_30143115
|         | | |    |         |         |         |                   | | |
          +**| (0/87)
|         | | |    |         |         |         |                   | | |
          |  +**HanXRQChr15_6435740_6441546
|         | | |    |         |         |         |                   | | |
          +--| (99/100)
|         | | |    |         |         |         |                   | | |
          |  +--HanXRQChr05_211734084_211734739
|         | | |    |         |         |         |                   | | |
          +**| (0/67)
|         | | |    |         |         |         |                   | | |
          |  +--HanXRQChr04_4816946_4817774
|         | | |    |         |         |         |                   | | |
          +--| (99.8/100)
|         | | |    |         |         |         |                   | | |
          |  +--HanXRQChr09_90613212_90613775
|         | | |    |         |         |         |                   | | |
          +--| (78.4/97)
|         | | |    |         |         |         |                   | | |
          | |       +--HanXRQChr14_164972043_164972420
```

```
|         | | |    |         |         |         |                  | | |
          | |  +--| (86.9/100)
|         | | |    |         |         |         |                  | | |
          | | | |  +--HanXRQChr17_171676962_171677479
|         | | |    |         |         |         |                  | | |
          | | |  +--| (75.8/100)
|         | | |    |         |         |         |                  | | |
          | | |    |  +--HanXRQChr04_11014761_11015279
|         | | |    |         |         |         |                  | | |
          | | |    +--| (75.4/100)
|         | | |    |         |         |         |                  | | |
          | | |    +--HanXRQChr04_11235868_11236386
|         | | |    |         |         |         |                  | | |
          |  +--| (98.6/100)
|         | | |    |         |         |         |                  | | |
          |        +--HanXRQChr10_36325499_36326037
|         | | |    |         |         |         |                  | | |
          +--| (71.1/69)
|         | | |    |         |         |         |                  | | |
          | |  +--HanXRQChr09_16583017_16583476
|         | | |    |         |         |         |                  | | |
          |  +--| (100/100)
|         | | |    |         |         |         |                  | | |
          |        +--HanXRQChr02_53194187_53194755
|         | | |    |         |         |         |                  | | |
          +--| (85.2/52)
|         | | |    |         |         |         |                  | | |
          |  +--HanXRQChr15_58262815_58263268
|         | | |    |         |         |         |                  | | |
          +--| (78.3/37)
|         | | |    |         |         |         |                  | | |
          | |                +--HanXRQChr03_53881858_53882406
|         | | |    |         |         |         |                  | | |
          | |                +--| (73.9/100)
|         | | |    |         |         |         |                  | | |
          | |                |  +**HanXRQChr15_50520164_50520709
|         | | |    |         |         |         |                  | | |
          | |      +--| (100/100)
|         | | |    |         |         |         |                  | | |
          | |      |  +--HanXRQChr08_58106590_58107151
|         | | |    |         |         |         |                  | | |
          | |      +**| (0/68)
|         | | |    |         |         |         |                  | | |
          | |      |  +--HanXRQChr04_117734534_117735067
```

| | | | | | | | | | |
| | +--| (88/58)
| | | | | | | | | | |
| | | +--HanXRQChr07_39909716_39910258
| | | | | | | | | | |
| | +--| (67.1/53)
| | | | | | | | | | |
| | | +--HanXRQChr11_114599910_114600468
| | | | | | | | | | |
| +**| (0/34)
| | | | | | | | | | |
| | +--HanXRQChr08_102456485_102456998
| | | | | | | | | | |
| | +--| (87.2/95)
| | | | | | | | | | |
| | | | +--HanXRQChr03_6367591_6368162
| | | | | | | | | | |
| | | +--| (57.2/95)
| | | | | | | | | | |
| | | | +**HanXRQChr08_32332017_32332576
| | | | | | | | | | |
| | | +--| (99.7/100)
| | | | | | | | | | |
| | | +**HanXRQChr05_142623602_142624161
| | | | | | | | | | |
| +--| (76.8/60)
| | | | | | | | | | |
| | +--HanXRQChr10_160816414_160816781
| | | | | | | | | | |
| | +--| (96.5/99)
| | | | | | | | | | |
| | | +--HanXRQChr09_205724496_205724897
| | | | | | | | | | |
| | +**| (0/57)
| | | | | | | | | | |
| | | +--HanXRQChr05_5609714_5610098
| | | | | | | | | | |
| +--| (100/100)
| | | | | | | | | | |
| +--HanXRQChr16_19443969_19444339
| | | | | | | | | | | +--|
(64/35)
| | | | | | | | | | | | |
+--HanXRQChr01_65087231_65087766

| | | | | | | | | | | | |
+--| (92.1/100)
| | | | | | | | | | | | |
| +--HanXRQChr10_26225253_26225786
| | | | | | | | | | | | |
+--| (96.6/100)
| | | | | | | | | | | | |
| +--HanXRQChr09_6730323_6730857
| | | | | | | | | | | | | +--|
(80.1/98)
| | | | | | | | | | | | | |
+--HanXRQChr04_145024358_145024790
| | | | | | | | | | | | +--|
(91.3/96)
| | | | | | | | | | | |
+--HanXRQChr12_103007227_103007661
| | | | | | | | | | | +--|
(74.7/47)
| | | | | | | | | | | | |
+--HanXRQChr08_56688339_56688696
| | | | | | | | | | | | |
+--| (70.4/96)
| | | | | | | | | | | | |
| | +**HanXRQChr03_150064680_150065184
| | | | | | | | | | | | |
| | +**| (0/61)
| | | | | | | | | | | | |
| | | +**HanXRQChr03_126121322_126121826
| | | | | | | | | | | | |
| +--| (96.2/100)
| | | | | | | | | | | | |
| +--HanXRQChr10_78375584_78376088
| | | | | | | | | | | | |
+--| (95.5/100)
| | | | | | | | | | | | |
| +**HanXRQChr07_12736562_12737068
| | | | | | | | | | | | |
+--| (99.4/100)
| | | | | | | | | | | | |
| +--HanXRQChr05_164461706_164462192
| | | | | | | | | | | | | +--|
(71.7/55)
| | | | | | | | | | | | | | |
+**HanXRQChr13_134302109_134302509

| | | | | | | | | | | | | | |
          +--| (98.9/100)
| | | | | | | | | | | | | | |
          | | +**HanXRQChr10_95181243_95181772
| | | | | | | | | | | | | | |
          | +--| (94.5/100)
| | | | | | | | | | | | | | |
          | +**HanXRQChr17_160614824_160615353
| | | | | | | | | | | | | | |
+**| (0/51)
| | | | | | | | | | | | | | |
| | +--HanXRQChr17_13361200_13361728
| | | | | | | | | | | | | | |
| | +--| (77.2/100)
| | | | | | | | | | | | | | |
| | | | +--HanXRQChr12_75917343_75917873
| | | | | | | | | | | | | | |
| | | | +--| (92.3/100)
| | | | | | | | | | | | | | |
| | | | | +**HanXRQChr14_86341928_86342457
| | | | | | | | | | | | | | |
| | | +--| (74/100)
| | | | | | | | | | | | | | |
| | | +--HanXRQChr03_99988684_99989213
| | | | | | | | | | | | | | |
| | +--| (84.2/100)
| | | | | | | | | | | | | | |
| | | +--HanXRQChr17_99228227_99228757
| | | | | | | | | | | | | | |
| +--| (53.3/92)
| | | | | | | | | | | | | | |
| | +--HanXRQChr16_15638745_15639274
| | | | | | | | | | | | | | |
| +--| (79.6/100)
| | | | | | | | | | | | | | |
| +**HanXRQChr05_144936503_144937032
| | | | | | | | | | | | | |
+--| (99.9/100)
| | | | | | | | | | | | | |
          +--HanXRQChr02_114840517_114841046
| | | | | | | | | | | | +--|
(90.2/56)
| | | | | | | | | | | |
          | +--HanXRQChr11_55711325_55711859

```
|        | | |    |        |        |        |                | | |                                  |
         |        +--| (82.6/73)
|        | | |    |        |        |        |                | | |                                  |
         |        | |  +--HanXRQChr01_29157382_29157877
|        | | |    |        |        |        |                | | |                                  |
         |        |  +--| (78.8/98)
|        | | |    |        |        |        |                | | |                                  |
         |        |        +--HanXRQChr13_104941607_104942137
|        | | |    |        |        |        |                | | |                                  |
         |  +--| (82.4/66)
|        | | |    |        |        |        |                | | |                                  |
         | | |  +--HanXRQChr08_25571094_25571648
|        | | |    |        |        |        |                | | |                                  |
         | |  +--| (100/100)
|        | | |    |        |        |        |                | | |                                  |
         | |      +--HanXRQChr05_44360152_44360708
|        | | |    |        |        |        |                | | |                                  |
         +--| (70.3/53)
|        | | |    |        |        |        |                | | |                                  |
         +--HanXRQChr10_187118315_187118736
|        | | |    |        |        |        |                | | |                                  +**|
(0/42)
|        | | |    |        |        |        |                | | |                                  | |
         +--HanXRQChr09_8264746_8265314
|        | | |    |        |        |        |                | | |                                  | |  +--|
(98.2/100)
|        | | |    |        |        |        |                | | |                                  | | |
+--HanXRQChr16_71647538_71648109
|        | | |    |        |        |        |                | | |                                  |  +--|
(84.3/77)
|        | | |    |        |        |        |                | | |                                  |
         |  +--HanXRQChr13_88688183_88688669
|        | | |    |        |        |        |                | | |                                  |
         +--| (100/100)
|        | | |    |        |        |        |                | | |                                  |
         +--HanXRQChr17_31920748_31921235
|        | | |    |        |        |        |                | | |                                  +--|
(93.2/57)
|        | | |    |        |        |        |                | | |                                  | |
         +--HanXRQChr17_84099735_84100241
|        | | |    |        |        |        |                | | |                                  | |  +--|
(88.5/99)
|        | | |    |        |        |        |                | | |                                  | | |
+--HanXRQChr10_11267406_11267923
```

| | | | | | | | | | | | +--| (80.9/84)
| | | | | | | | | | | |
| +**HanXRQChr02_131243878_131244246
| | | | | | | | | | | |
+--| (99.9/100)
| | | | | | | | | | | |
+--HanXRQChr10_168209872_168210240
| | | | | | | | | | | +--| (95.6/90)
| | | | | | | | | | | | | +--HanXRQChr08_113839738_113840105
| | | | | | | | | | | | | +--| (74.1/96)
| | | | | | | | | | | | | | +--HanXRQChr04_28158629_28159233
| | | | | | | | | | | | +--| (100/100)
| | | | | | | | | | | | +--HanXRQChr11_39830792_39831397
| | | | | | | | | | | +--| (84.9/64)
| | | | | | | | | | | | |
+**HanXRQChr11_133792609_133792984
| | | | | | | | | | | | | +--| (98.3/100)
| | | | | | | | | | | | | |
+**HanXRQChr11_133775764_133776143
| | | | | | | | | | | | | +--| (87.5/100)
| | | | | | | | | | | | | | +--HanXRQChr05_62558786_62559318
| | | | | | | | | | | | +--| (95.1/100)
| | | | | | | | | | | | | +--HanXRQChr09_124910897_124911250
| | | | | | | | | | | | +--| (100/100)
| | | | | | | | | | | | +--HanXRQChr11_30817527_30817892
| | | | | | | | | | | +--| (87.4/80)
| | | | | | | | | | | | | +--HanXRQChr17_68193643_68194142
| | | | | | | | | | | | +--| (100/100)

| | | | | | | | | | | | +--
HanXRQChr11_94539491_94539990
| | | | | | | | | | | +--| (86.6/39)
| | | | | | | | | | | | |
+--HanXRQChr01_40849696_40850225
| | | | | | | | | | | | | +**|
(0/90)
| | | | | | | | | | | | | | +--
HanXRQChr11_135844467_135845030
| | | | | | | | | | | | | +--|
(99.6/100)
| | | | | | | | | | | | | | | +--
HanXRQChr16_45835607_45836135
| | | | | | | | | | | | | | +--|
(85/100)
| | | | | | | | | | | | | |
+--HanXRQChr04_141436125_141436654
| | | | | | | | | | | | | +--|
(77.2/79)
| | | | | | | | | | | | | | +--
HanXRQChr14_73178118_73178607
| | | | | | | | | | | | | +--|
(48.8/41)
| | | | | | | | | | | | | | | +--
HanXRQChr09_74883466_74883949
| | | | | | | | | | | | | | +--|
(99.9/100)
| | | | | | | | | | | | | | +--
HanXRQChr05_179907532_179908035
| | | | | | | | | | | | +**| (0/25)
| | | | | | | | | | | | | +--
HanXRQChr10_170724767_170725228
| | | | | | | | | | | | +--|
(93/95)
| | | | | | | | | | | | | +--
HanXRQChr11_114441513_114442047
| | | | | | | | | | | | +--|
(84.7/98)
| | | | | | | | | | | | +--
HanXRQChr08_22016304_22016849
| | | | | | | | | | | +--| (62.1/48)
| | | | | | | | | | | | |
+--HanXRQChr17_93185904_93186267

```
|         | | |    |         |         |         |                 | | |                     | |
          +**| (0/56)
|         | | |    |         |         |         |                 | | |                     | |
          |  +--HanXRQChr17_93126497_93127118
|         | | |    |         |         |         |                 | | |                     | |
          +--| (98/100)
|         | | |    |         |         |         |                 | | |                     | |
          |  +--HanXRQChr17_93164704_93165330
|         | | |    |         |         |         |                 | | |                     | |
          +**| (0/22)
|         | | |    |         |         |         |                 | | |                     | |
          | |        +--HanXRQChr04_162198911_162199522
|         | | |    |         |         |         |                 | | |                     | |
          | |        +--| (97/100)
|         | | |    |         |         |         |                 | | |                     | |
          | |        |  +--HanXRQChr17_31095021_31095488
|         | | |    |         |         |         |                 | | |                     | |
          | |  +--| (84.9/89)
|         | | |    |         |         |         |                 | | |                     | |
          | | | |  +--HanXRQChr16_139484437_139485058
|         | | |    |         |         |         |                 | | |                     | |
          | | |  +**| (0/60)
|         | | |    |         |         |         |                 | | |                     | |
          | | |    |         +--HanXRQChr06_73271993_73272461
|         | | |    |         |         |         |                 | | |                     | |
          | | |    |  +**| (0/76)
|         | | |    |         |         |         |                 | | |                     | |
          | | |    | | |  +--HanXRQChr17_13434430_13434837
|         | | |    |         |         |         |                 | | |                     | |
          | | |    | |  +--| (96.2/100)
|         | | |    |         |         |         |                 | | |                     | |
          | | |    | |       |  +--HanXRQChr05_171432714_171433335
|         | | |    |         |         |         |                 | | |                     | |
          | | |    | |       +**| (0/87)
|         | | |    |         |         |         |                 | | |                     | |
          | | |    | |       +--HanXRQChr02_40423347_40423968
|         | | |    |         |         |         |                 | | |                     | |
          | | |    +**| (0/93)
|         | | |    |         |         |         |                 | | |                     | |
          | | |    +--HanXRQChr10_144765081_144765648
|         | | |    |         |         |         |                 | | |                     | |
          |  +**| (0/51)
|         | | |    |         |         |         |                 | | |                     | |
          |        |         +--HanXRQChr13_193729788_193730410
```

| | | | | | | | | | | | | |
| | +--| (84.7/100)
| | | | | | | | | | | | | |
| | | +--HanXRQChr02_111025526_111026149
| | | | | | | | | | | | | |
| | +**| (0/93)
| | | | | | | | | | | | | |
| | | +--HanXRQChr01_83910798_83911405
| | | | | | | | | | | | | |
| | +--| (85.2/84)
| | | | | | | | | | | | | |
| | | | +--HanXRQChr14_28912143_28912767
| | | | | | | | | | | | | |
| | | +**| (0/48)
| | | | | | | | | | | | | |
| | | +--HanXRQChr11_61241453_61242074
| | | | | | | | | | | | | |
| | +**| (0/41)
| | | | | | | | | | | | | |
| | | | +**HanXRQChr15_64901712_64902075
| | | | | | | | | | | | | |
| | | +--| (97.2/100)
| | | | | | | | | | | | | |
| | | +**HanXRQChr04_57314850_57315475
| | | | | | | | | | | | | |
| +--| (83.2/82)
| | | | | | | | | | | | | |
| +--HanXRQChr02_40199887_40200279
| | | | | | | | | | | | | |
+**| (0/29)
| | | | | | | | | | | | | |
| +--HanXRQChr17_139322691_139323166
| | | | | | | | | | | | | | +--|
(24.4/32)
| | | | | | | | | | | | | | | |
+--HanXRQChr10_145087013_145087418
| | | | | | | | | | | | | | | |
+--| (88.3/100)
| | | | | | | | | | | | | | | |
| +--HanXRQChr10_175382210_175382618
| | | | | | | | | | | | | | | | +--|
(82.8/100)
| | | | | | | | | | | | | | | | |
+--HanXRQChr06_56355680_56356069

| | | | | | | | | | | | | | +--|
(77.4/93)
| | | | | | | | | | | | | |
| +--HanXRQChr17_95938863_95939232
| | | | | | | | | | | | | |
+--| (98.5/99)
| | | | | | | | | | | | | |
+--HanXRQChr13_144225259_144225863
| | | | | | | | | | | | | +--|
(100/100)
| | | | | | | | | | | | | | +--
HanXRQChr07_94174100_94174724
| | | | | | | | | | | | | +--|
(88.8/92)
| | | | | | | | | | | | | | |
+--HanXRQChr15_52286990_52287513
| | | | | | | | | | | | | | |
+--| (68.5/100)
| | | | | | | | | | | | | | |
| | +**HanXRQChr09_19029933_19030470
| | | | | | | | | | | | | | |
| +--| (85.9/100)
| | | | | | | | | | | | | | |
| +--HanXRQChr09_126494259_126494796
| | | | | | | | | | | | | | |
+--| (99.8/100)
| | | | | | | | | | | | | | |
| +--HanXRQChr06_41524728_41525252
| | | | | | | | | | | | | | | +**|
(0/86)
| | | | | | | | | | | | | | | | |
+--HanXRQChr03_26626677_26627224
| | | | | | | | | | | | | | | |
+--| (96.4/100)
| | | | | | | | | | | | | | | |
+--HanXRQChr12_100245888_100246277
| | | | | | | | | | | | | | +--|
(58.7/96)
| | | | | | | | | | | | | |
+--HanXRQChr14_47500657_47501050
| | | | | | | | | | | | | +--|
(3.4/53)
| | | | | | | | | | | | | | |
+**HanXRQChr14_79508506_79508881

| | | | | | | | | | | | | | | |
+--| (85.5/99)
| | | | | | | | | | | | | | | |
| +--HanXRQChr15_145112060_145112588
| | | | | | | | | | | | | | | |
+--| (75.4/100)
| | | | | | | | | | | | | | | |
| +--HanXRQChr09_159439793_159440170
| | | | | | | | | | | | | | | |
+**| (0/55)
| | | | | | | | | | | | | | | |
| +--HanXRQChr14_45570798_45571311
| | | | | | | | | | | | | | | |
+--| (87.5/100)
| | | | | | | | | | | | | | | |
| | +--HanXRQChr16_170010412_170010812
| | | | | | | | | | | | | | | |
| +--| (76/100)
| | | | | | | | | | | | | | | |
| +--HanXRQChr01_83270623_83271135
| | | | | | | | | | | | | | | |
+--| (75.7/77)
| | | | | | | | | | | | | | | |
| +--HanXRQChr10_173822886_173823254
| | | | | | | | | | | | | | | |
+--| (80.8/75)
| | | | | | | | | | | | | | | |
| +--HanXRQChr04_147140553_147141079
| | | | | | | | | | | | | | | |
+**| (0/46)
| | | | | | | | | | | | | | | |
| | +--HanXRQChr09_59706805_59707181
| | | | | | | | | | | | | | | |
| | +--| (77.3/94)
| | | | | | | | | | | | | | | |
| | | +--HanXRQChr04_65301484_65302016
| | | | | | | | | | | | | | | |
| +--| (78.8/85)
| | | | | | | | | | | | | | | |
| +--HanXRQChr07_35619467_35619941
| | | | | | | | | | | | | | | | +--|
(0/59)
| | | | | | | | | | | | | | | | |
+--HanXRQChr02_46053364_46053773

| | | | | | | | | | | | | | | +--|
(99/100)
| | | | | | | | | | | | | |
+--HanXRQChr10_100532038_100532438
| | | | | | | | | | | | | +--| (89.4/93)
| | | | | | | | | | | | | | | +--
HanXRQChr15_125481128_125481695
| | | | | | | | | | | | | | +--|
(100/100)
| | | | | | | | | | | | | | +--
HanXRQChr10_140012131_140012699
| | | | | | | | | | | | | +--| (56/85)
| | | | | | | | | | | | | | | +--
HanXRQChr15_153093160_153093558
| | | | | | | | | | | | | | +--|
(67.3/94)
| | | | | | | | | | | | | | +--
HanXRQChr11_77517539_77518062
| | | | | | | | | | | | | +--| (79.8/24)
| | | | | | | | | | | | | | |
+**HanXRQChr09_48002641_48003124
| | | | | | | | | | | | | | +--|
(100/100)
| | | | | | | | | | | | | |
+**HanXRQChr09_48019973_48020456
| | | | | | | | | | | | | +**| (0/16)
| | | | | | | | | | | | | | | +--
HanXRQChr17_54907212_54907721
| | | | | | | | | | | | | | +--| (100/100)
| | | | | | | | | | | | | | +--
HanXRQChr09_50143993_50144471
| | | | | | | | | | | | +--| (22.7/17)
| | | | | | | | | | | | +--
HanXRQChr07_67769_68326
| | | | | | | | | | | +--| (42.2/53)
| | | | | | | | | | | | +--
HanXRQChr10_170627841_170628410
| | | | | | | | | | +---------------------------| (100/100)
| | | | | | | | | | +--
HanXRQChr13_127794694_127795300
| | | | | | | | +--| (85.8/89)
| | | | | | | | +--HanXRQChr12_94877770_94878735
| | | | | | | +--| (88.8/95)

```
|         | | |    |         |         | | |                              +--
HanXRQChr16_150636591_150640613
|         | | |    |         |         | | |                              +--| (93.4/100)
|         | | |    |         |         | | |                              | |       +--
HanXRQChr17_47637609_47642782
|         | | |    |         |         | | |                              | |       +**| (0/92)
|         | | |    |         |         | | |                              | |       |  +--
HanXRQChr09_42030904_42038738
|         | | |    |         |         | | |                              | |  +--| (86.6/100)
|         | | |    |         |         | | |                              | | |  +--
HanXRQChr08_88220498_88221890
|         | | |    |         |         | | |                              |  +--| (89.6/100)
|         | | |    |         |         | | |                              |          +--
HanXRQChr15_74394306_74402182
|         | | |    |         |         | | |                    +--| (93.6/98)
|         | | |    |         |         | | |                    |  +--
HanXRQChr05_177102059_177102727
|         | | |    |         |         | | |                    +--| (74/98)
|         | | |    |         |         | | |                    |  +--
HanXRQChr05_199634091_199642069
|         | | |    |         |         | | |                    +--| (95.2/99)
|         | | |    |         |         | | |                    |  +--HanXRQChr09_26818251_26820777
|         | | |    |         |         | | |                    +--| (4.2/70)
|         | | |    |         |         | | |                    |  +--HanXRQChr03_34702985_34703845
|         | | |    |         |         | | |          +--| (85.3/97)
|         | | |    |         |         | | |          | |       +--HanXRQChr17_164543226_164545213
|         | | |    |         |         | | |          | |  +**| (0/93)
|         | | |    |         |         | | |          | | | |  +**HanXRQChr11_5059413_5062302
|         | | |    |         |         | | |          | | |  +--| (96.6/100)
|         | | |    |         |         | | |          | | |     +**HanXRQChr16_159464039_159466946
|         | | |    |         |         | | |          |  +--| (96.9/100)
|         | | |    |         |         | | |          |          |         +--
HanXRQChr04_4585394_4586317
|         | | |    |         |         | | |          |          |  +--| (72/100)
|         | | |    |         |         | | |          |          | |  +**HanXRQChr12_8060455_8064354
|         | | |    |         |         | | |          |          +--| (100/100)
|         | | |    |         |         | | |          |          +--HanXRQChr04_4414273_4418183
|         | | |    |         |         | | |          +--| (85.8/100)
|         | | |    |         |         | | |          | |       +--HanXRQChr16_154231921_154234094
|         | | |    |         |         | | |          | |       +--| (87.4/100)
|         | | |    |         |         | | |          | |       | |  +--
HanXRQChr10_201208107_201211833
|         | | |    |         |         | | |          | |       |  +--| (57.7/100)
```

```
|        | | |    |       |       | | |           | |     |       +--
HanXRQChr06_1335657_1337253
|        | | |    |       |       | | |           | | +--| (82.4/95)
|        | | |    |       |       | | |           | | | |  +--HanXRQChr09_1135754_1136217
|        | | |    |       |       | | |           | | | |  +--| (99.7/100)
|        | | |    |       |       | | |           | | | | |  +--HanXRQChr09_1132214_1133064
|        | | |    |       |       | | |           | | |  +--| (97.3/99)
|        | | |    |       |       | | |           | | |    |  +--
HanXRQChr14_171048254_171055488
|        | | |    |       |       | | |           | | |    +--| (98.7/100)
|        | | |    |       |       | | |           | | |    +--HanXRQChr02_147305501_147307299
|        | | |    |       |       | | |           |  +--| (81.3/96)
|        | | |    |       |       | | |           |        |  +--HanXRQChr06_47763415_47764638
|        | | |    |       |       | | |           |        +--| (97.9/100)
|        | | |    |       |       | | |           |        +--HanXRQChr12_40589262_40592252
|        | | |    |       |       | | |           +--| (41.5/96)
|        | | |    |       |       | | |           |  +--HanXRQChr05_52352508_52353847
|        | | |    |       |       | | |           +--| (86.9/100)
|        | | |    |       |       | | |           |  +--HanXRQChr12_66452661_66454107
|        | | |    |       |       | | |    +--| (99.8/100)
|        | | |    |       |       | | |    |  +--HanXRQChr03_153476779_153477741
|        | | |    |       |       | | |    +--| (80/99)
|        | | |    |       |       | | |    |  +--HanXRQChr09_192317181_192318492
|        | | |    |       |       | | |  +--| (19.5/86)
|        | | |    |       |       | | | |  +--HanXRQChr09_173686390_173688466
|        | | |    |       |       | |  +--| (81.3/79)
|        | | |    |       |       | |    |        +--HanXRQChr07_27412393_27412825
|        | | |    |       |       | |    +-------| (100/100)
|        | | |    |       |       | |            +**HanXRQChr07_27480885_27481317
|        | | |    |       |       +--| (95.1/100)
|        | | |    |       |       |        +--HanXRQChr10_167996501_167997306
|        | | |    |       |       |  +--| (99.5/100)
|        | | |    |       |       | | |  +--HanXRQChr07_94263537_94264736
|        | | |    |       |       | |  +--| (98.8/100)
|        | | |    |       |       | |    |        +--HanXRQChr10_200122501_200126511
|        | | |    |       |       | |    |        +--| (87.8/100)
|        | | |    |       |       | |    |        |  +--HanXRQChr03_159773788_159776353
|        | | |    |       |       | |    |        +--| (95.2/100)
|        | | |    |       |       | |    |        |  +--HanXRQChr16_117885695_117887257
|        | | |    |       |       | |    |  +**| (0/85)
|        | | |    |       |       | |    | |  +--HanXRQChr16_139387141_139394695
|        | | |    |       |       | |    +--| (97/100)
|        | | |    |       |       | |    +--HanXRQChr13_70292420_70293133
|        | | |    |       |       +--| (99.6/100)
```

```
|       | | |   |       |       |               +--HanXRQChr12_74630615_74638291
|       | | |   |       |       |       +--| (97.9/100)
|       | | |   |       |       |       | | +--HanXRQChr05_32799874_32806012
|       | | |   |       |       |       | +--| (78.8/100)
|       | | |   |       |       |       |       +--HanXRQChr05_66536036_66536958
|       | | |   |       |       |       +--| (71.6/99)
|       | | |   |       |       |       | | +--HanXRQChr09_42108185_42108601
|       | | |   |       |       |       | +--| (74/100)
|       | | |   |       |       |       |       |       +--HanXRQChr14_70675600_70678361
|       | | |   |       |       |       |       | +**| (0/99)
|       | | |   |       |       |       |       | | | +--HanXRQChr14_79208902_79211665
|       | | |   |       |       |       |       | | +**| (0/91)
|       | | |   |       |       |       |       | |     +--HanXRQChr14_77501682_77504443
|       | | |   |       |       |       |       +--| (73.9/100)
|       | | |   |       |       |       |       +--HanXRQChr02_175774663_175777915
|       | | |   |       |       |       +--| (99.1/100)
|       | | |   |       |       |       | +--HanXRQChr08_40489307_40491198
|       | | |   |       |       |       +--| (69.5/81)
|       | | |   |       |       |       | | +--HanXRQChr09_184856661_184857251
|       | | |   |       |       |       | +--| (99.8/100)
|       | | |   |       |       |       |       +--HanXRQChr13_144823475_144824100
|       | | |   |       |       | +--| (75.4/57)
|       | | |   |       |       | | | +--HanXRQChr15_104285615_104286058
|       | | |   |       |       | | +--| (94.7/100)
|       | | |   |       |       | |     +--HanXRQChr05_209135841_209137273
|       | | |   |       |       +--| (27.1/63)
|       | | |   |       |       +--HanXRQChr16_128055830_128059471
|       | | |   | +--| (40.9/98)
|       | | |   | | +--HanXRQChr11_34832223_34833041
|       | | |   +--| (82.6/100)
|       | | |   +--HanXRQChr03_69429483_69433013
|       | +--| (97.2/100)
|       |       +--HanXRQChr04_162448231_162451209
| +--| (95.9/100)
| | | +--HanXRQChr13_159695079_159696066
| | +--| (89.8/100)
| |     +--HanXRQChr06_34593592_34601130
+--| (79.2/98)
  |             +**HanXRQChr07_27634064_27638140
  |             +**| (0/99)
  |             | +--HanXRQChr05_64416693_64422326
  |     +--| (18.2/85)
  |     | | +--HanXRQChr15_23210956_23213675
  |     | +--| (90.5/99)
```

```
|       |          +--HanXRQChr11_69846093_69853687
|       +--| (91.6/98)
|       |  |  +**HanXRQChr06_20451348_20451704
|       |  +**| (0/97)
|       |          +--HanXRQChr13_179591469_179595652
|       +**| (0/80)
|       |  |       +**HanXRQChr12_45905678_45909951
|       |  |       +--| (89.6/100)
|       |  |       |  +**HanXRQChr15_74224040_74228313
|       |  |  +**| (0/67)
|       |  |  |  +--HanXRQChr13_2986278_2987762
|       |  +**| (0/67)
|       |          +**HanXRQChr02_4724981_4726023
|  +--| (89.8/100)
|  |  +--HanXRQChr13_135223336_135224153
+--| (96.4/100)
        +--HanXRQChr13_196286900_196290049
```

Tree in newick format:

(HanXRQChr11_78963679_78964101:0.0407776336,((((HanXRQChr01_34887426_34894282:
0.0043297442,(HanXRQChr12_24613340_24614343:0.0000022540,HanXRQChr08_22937900
_22939039:0.0000022540)0/95:0.0000022540)0/92:0.0000021621,HanXRQChr13_45702842_
45703840:0.0086767326)87.6/98:0.0043269821,HanXRQChr17_205330461_205331450:0.004
7454988)0/98:0.0000029605,HanXRQChr03_26205754_26213308:0.0130899865)76.7/100:0.0
048715417,(((HanXRQChr04_67540092_67541320:0.0216295207,((HanXRQChr06_27984911
_27988051:0.0130756142,(HanXRQChr04_12175865_12181403:0.0223904963,((((HanXRQC
hr17_211327788_211333859:0.0131105633,(HanXRQChr05_59284493_59290538:0.0000022
540,HanXRQChr05_106376295_106382341:0.0000022540)98.4/100:0.0270642086)90.6/100:0
.0149190887,((HanXRQChr09_48889131_48889670:0.0526367015,HanXRQChr15_55516101
_55518531:0.0431452481)83.6/99:0.0253881334,((((((HanXRQChr03_57480187_57481416:0.
0473632306,(HanXRQChr05_166779247_166780763:0.0869229259,HanXRQChr12_9487586
4_94876484:0.1080360370)79.5/100:0.0113857758)0/80:0.0000029488,(HanXRQChr04_9241
8435_92419412:0.0044649344,(HanXRQChr06_53262309_53263292:0.0000028097,HanXRQ
Chr05_9034545_9035531:0.0087871563)75.5/100:0.0042873327)100/100:0.1911173534)80.6/
99:0.0119082793,HanXRQChr11_113608046_113608769:0.0919898626)95.9/99:0.043746738
0,((((HanXRQChr16_94477431_94477942:0.1508149849,((((HanXRQChr17_67579875_67582
613:0.0706645722,HanXRQChr11_142203384_142204256:0.1226652255)99.9/100:0.0972337
935,HanXRQChr04_176555457_176557628:0.0487691419)0/88:0.0000029528,HanXRQChr01
_116026086_116027124:0.0273901164)73.8/96:0.0044776186,HanXRQChr13_137859189_13
7859594:0.0260378128)99.9/100:0.1111983753)70.6/96:0.0095799038,HanXRQChr10_15262
4866_152626709:0.0607217246)79/97:0.0222102701,(((HanXRQChr07_57474618_57475394:
0.0178564920,HanXRQChr07_57434299_57438258:0.0265375732)82.2/100:0.0085698662,(H
anXRQChr05_190757444_190758598:0.0133619993,(HanXRQChr13_94278579_94280493:0.

0207500899,HanXRQChr04_115355662_115358538:0.0307573865)99.8/100:0.0688222028)4
3/100:0.0045823146)93/100:0.0236667681,(((HanXRQChr11_18867372_18868580:0.0000022
540,HanXRQChr02_20281946_20283260:0.0000022540)0/73:0.0000029696,HanXRQChr11_1
8749900_18751215:0.0043236120)98.5/100:0.0368296905,HanXRQChr06_28223014_282278
54:0.0255174809)79.9/98:0.0136583694)94.4/98:0.0440523126)100/100:0.1941979426,((HanX
RQChr12_88474680_88475105:0.2936417582,((((HanXRQChr01_1207903_1208488:0.02605
19686,(HanXRQChr08_151986814_151987353:0.0276449800,HanXRQChr10_94811711_948
12246:0.0585106205)97.9/100:0.0420011379)78/99:0.0182334635,(HanXRQChr05_16886133
0_168861873:0.0564628918,(HanXRQChr05_193946694_193947239:0.0212606032,HanXRQ
Chr05_193733456_193734002:0.0142023858)89.2/100:0.0272554672)98.5/100:0.0662155976
)87.6/99:0.0460086577,HanXRQChr05_182283860_182284319:0.0974523078)90/99:0.076573
6202,(HanXRQChr17_105500849_105501312:0.0618007745,((HanXRQChr04_16618909_166
19279:0.0384123611,(HanXRQChr01_146764857_146765256:0.0086020586,(HanXRQChr03_
20364521_20364920:0.0000022540,(HanXRQChr05_14305976_14306375:0.0000022540,Han
XRQChr16_185568115_185568603:0.0000025739)0/38:0.0000025738)0/76:0.0000020848)10
0/100:0.4708722576)62/60:0.0300378244,HanXRQChr03_113315527_113315992:0.09814465
48)84.8/62:0.0257007063)90.4/98:0.0826607494)100/100:0.4714316589)88.6/99:0.159624595
4,(((((HanXRQChr02_175754451_175758707:0.0523647971,HanXRQChr13_94161763_94165
162:0.0310485253)95.9/93:0.0477776294,(HanXRQChr11_38886892_38889248:0.057261343
1,(HanXRQChr05_164509744_164514326:0.0781800243,HanXRQChr03_34772558_3477346
8:0.1553594795)40/82:0.0309149599)93.2/93:0.0473447941)77.6/68:0.0296197486,((((HanXR
QChr08_33398524_33406006:0.0271464847,HanXRQChr12_40586043_40589256:0.0261652
586)95.1/100:0.0258350797,(HanXRQChr13_77076783_77077636:0.0533975075,((((HanXRQ
Chr10_115966867_115967362:0.0275735570,HanXRQChr17_164520103_164521773:0.03284
96378)0/14:0.0000022540,(HanXRQChr01_49628126_49635945:0.0091247445,HanXRQChr1
4_68245992_68247226:0.0371039293)0/34:0.0000027375)0/19:0.0000029466,(HanXRQChr09
_125961449_125964480:0.0326371860,((HanXRQChr06_28014629_28015962:0.0327449809,
HanXRQChr11_23461540_23465141:0.0136010567)72.3/100:0.0046539154,HanXRQChr04_1
66379772_166382442:0.0564309889)77.7/76:0.0042338752)76.4/70:0.0046063042)78.4/76:0.
0057556384,((HanXRQChr04_174582120_174583103:0.0492630832,HanXRQChr13_1352245
56_135226019:0.1029639510)87.6/99:0.0229581538,(HanXRQChr07_11945240_11945839:0.
0474059547,HanXRQChr05_23977914_23979210:0.0671674222)86.5/100:0.0177450769)81.1
/78:0.0105119247)80.2/99:0.0119280150)98.4/100:0.0410582421)38.4/85:0.0045427296,HanX
RQChr03_50693386_50694309:0.0149071054)93.4/99:0.0341485821,HanXRQChr08_404743
25_40480066:0.0770203114)84/98:0.0254422125)98.5/91:0.0865369392,((((((((HanXRQChr02
_24653810_24657437:0.0045017656,(HanXRQChr14_68289895_68297518:0.0090718466,Ha
nXRQChr14_68273516_68281146:0.0137250959)87/100:0.0091655354)87.4/100:0.009312117
6,((((((HanXRQChr02_147295547_147297741:0.0000022540,HanXRQChr09_142942803_142
944567:0.0000022540)88.1/100:0.0091947846,HanXRQChr13_96620660_96621156:0.013653
6302)74.4/100:0.0044642353,HanXRQChr04_132271481_132272204:0.0183363079)89.6/100:
0.0132539545,HanXRQChr14_153324342_153328229:0.0223570017)72.9/100:0.0061538858,
HanXRQChr10_85192350_85197513:0.0178563787)74.5/100:0.0088050290,((HanXRQChr06_
97006962_97010930:0.0135931833,HanXRQChr03_159793635_159795071:0.0091836909)84
.7/100:0.0092125465,(((HanXRQChr07_88885423_88893193:0.0090759984,HanXRQChr08_3

9439464_39440150:0.0000022540)0/34:0.0000027732,HanXRQChr08_109696851_10969759
3:0.0045221872)98.8/100:0.0276009398,(HanXRQChr01_140346486_140347133:0.02347490
75,HanXRQChr16_117883570_117885696:0.0087711249)75.9/99:0.0046483713)84.5/95:0.00
91211601)100/100:0.0716579927)96.3/91:0.0234651610)85.8/90:0.0092517031,HanXRQChr1
0_225800164_225801136:0.0133397565)96.4/90:0.0302428199,((HanXRQChr04_79257170_7
9260978:0.0320161806,(HanXRQChr09_141496442_141499885:0.0140330328,HanXRQChr0
3_70542853_70547885:0.0232302253)0/85:0.0000026830)97.2/100:0.0312372069,HanXRQC
hr07_94262118_94263107:0.0519417234)75/88:0.0065991367)86.1/82:0.0143966349,((((Han
XRQChr17_170737481_170744938:0.0113430342,HanXRQChr04_115339901_115344808:0.0
148024803)28.7/88:0.0054042316,HanXRQChr05_177667685_177675472:0.0505881327)82.6
/100:0.0103000685,((((((((HanXRQChr13_179578065_179585597:0.0000022540,HanXRQChr1
3_179805528_179808054:0.0000022540)96.1/100:0.0137476474,(HanXRQChr05_64422906_
64424659:0.0045595707,HanXRQChr13_7315803_7323380:0.0135828196)77.5/99:0.0044297
585)0/56:0.0000022565,HanXRQChr15_23213675_23214340:0.0228608442)81.3/61:0.004418
1289,(HanXRQChr11_127530325_127537865:0.0090490660,HanXRQChr12_23644236_2364
5297:0.0137063518)0/67:0.0000027602)0/46:0.0000021827,(HanXRQChr14_116526279_1165
33821:0.0045679750,HanXRQChr17_205323932_205330231:0.0138613284)22.1/75:0.004479
8250)92.4/92:0.0225853693,(HanXRQChr17_3057459_3059571:0.0000021337,((HanXRQChr
13_169241697_169244853:0.0090258968,HanXRQChr01_90452614_90460077:0.013672630
9)0/68:0.0000020035,(HanXRQChr13_45704069_45710378:0.0043090049,HanXRQChr08_22
931465_22937801:0.0006403172)71.6/100:0.0040171480)79.3/100:0.0044479235)0/88:0.0000
021489)93.6/90:0.0271147419,HanXRQChr13_196274607_196275703:0.0528707548)89.5/10
0:0.0174325304,((HanXRQChr13_48436772_48438877:0.0090266680,(((HanXRQChr15_1293
12466_129317224:0.0044881732,HanXRQChr02_147809574_147817424:0.0044754188)0/57:
0.0000028697,(HanXRQChr14_171031165_171032099:0.0041730131,HanXRQChr10_113962
499_113970363:0.0149683844)99.9/100:0.0772497137)82/89:0.0044783531,(HanXRQChr11_
23755532_23757425:0.0090277958,HanXRQChr01_4643702_4650088:0.0044793681)0/67:0.
0000026492)0/81:0.0000021179)85.6/89:0.0089358579,HanXRQChr08_88222000_88228288:
0.0227821493)85.6/97:0.0100345678)90.5/94:0.0193777807)96.1/100:0.0373605244,HanXRQ
Chr15_150283897_150291604:0.0378225084)22.8/91:0.0192862668)93.3/99:0.0328039340,((
HanXRQChr03_46527653_46534444:0.0383866676,HanXRQChr06_51391635_51395795:0.0
240643240)66.4/100:0.0028043650,HanXRQChr07_44435735_44436200:0.0286740369)94.8/
100:0.0316833580)96.6/99:0.0446324539,HanXRQChr10_167884487_167885281:0.06633253
82)37.5/70:0.0240914028,(((HanXRQChr16_149753884_149754258:0.0817025581,HanXRQC
hr01_98528860_98530430:0.0545971110)21.8/78:0.0131965369,HanXRQChr16_128066409_
128067498:0.0582553305)79.7/69:0.0161667829,((HanXRQChr02_20242486_20243525:0.030
3194821,HanXRQChr11_18762949_18764264:0.0158245202)83.2/100:0.0180498032,HanXR
QChr16_148694596_148701069:0.0718640380)86.2/100:0.0215411710)90.5/72:0.0341805061
)43.7/75:0.0498962569)68.7/85:0.0228914840,(((HanXRQChr15_23293519_23294345:0.1233
060736,(HanXRQChr06_29856663_29857319:0.1934857608,HanXRQChr17_60426850_6042
8287:0.8448826242)99.2/100:0.3508927069)24.5/79:0.0341572532,(((((((((((((((((((HanXRQChr
07_30137334_30143115:0.0089336804,HanXRQChr15_6435740_6441546:0.0000027405)0/8
7:0.0000028417,HanXRQChr05_211734084_211734739:0.0044645276)99/100:0.0360458257,
HanXRQChr04_4816946_4817774:0.0509362853)0/67:0.0000021855,HanXRQChr09_906132

12_90613775:0.0473501345)99.8/100:0.0709927876,((HanXRQChr14_164972043_164972420
:0.0176081468,(HanXRQChr17_171676962_171677479:0.0090450629,(HanXRQChr04_11014
761_11015279:0.0135846119,HanXRQChr04_11235868_11236386:0.0135869997)75.4/100:0.
0044199875)75.8/100:0.0049145362)86.9/100:0.0134131108,HanXRQChr10_36325499_3632
6037:0.0142825010)98.6/100:0.0492780091)78.4/97:0.0112058180,(HanXRQChr09_16583017
_16583476:0.0103701224,HanXRQChr02_53194187_53194755:0.0174863589)100/100:0.100
2315154)71.1/69:0.0051124004,HanXRQChr15_58262815_58263268:0.1301898811)85.2/52:0
.0185846920,((((((HanXRQChr03_53881858_53882406:0.0089745142,HanXRQChr15_505201
64_50520709:0.0000022540)73.9/100:0.0042153214,HanXRQChr08_58106590_58107151:0.0
138608747)100/100:0.0674867821,HanXRQChr04_117734534_117735067:0.0811966467)0/6
8:0.0000028289,HanXRQChr07_39909716_39910258:0.1080716237)88/58:0.0142740168,Han
XRQChr11_114599910_114600468:0.0994928958)67.1/53:0.0029856485,((HanXRQChr08_10
2456485_102456998:0.0814209307,(HanXRQChr03_6367591_6368162:0.1361766466,(HanX
RQChr08_32332017_32332576:0.0000022540,HanXRQChr05_142623602_142624161:0.0000
021945)99.7/100:0.0890270409)57.2/95:0.0309329017)87.2/95:0.0229367787,(((HanXRQChr1
0_160816414_160816781:0.0132283323,HanXRQChr09_205724496_205724897:0.02815782
08)96.5/99:0.0400857522,HanXRQChr05_5609714_5610098:0.2036870927)0/57:0.000002905
3,HanXRQChr16_19443969_19444339:0.1008989341)100/100:0.1959244221)76.8/60:0.00720
72125)0/34:0.0000024330)78.3/37:0.0066563544,((((HanXRQChr01_65087231_65087766:0.0
135556208,HanXRQChr10_26225253_26225786:0.0089797325)92.1/100:0.0281842623,HanX
RQChr09_6730323_6730857:0.0343125126)96.6/100:0.0486344545,HanXRQChr04_1450243
58_145024790:0.0773043241)80.1/98:0.0251962880,HanXRQChr12_103007227_103007661:
0.1261615623)91.3/96:0.0325230912)64/35:0.0031014688,(((((HanXRQChr08_56688339_566
88696:0.0182035568,((HanXRQChr03_150064680_150065184:0.0000022540,HanXRQChr03_
126121322_126121826:0.0000022540)0/61:0.0000022540,HanXRQChr10_78375584_783760
88:0.0044945125)96.2/100:0.0189917507)70.4/96:0.0046338344,HanXRQChr07_12736562_1
2737068:0.0000020665)95.5/100:0.0395982852,HanXRQChr05_164461706_164462192:0.053
4316573)99.4/100:0.0701332590,(((HanXRQChr13_134302109_134302509:0.0000020743,(Ha
nXRQChr10_95181243_95181772:0.0000022540,HanXRQChr17_160614824_160615353:0.00
00022540)94.5/100:0.0133594256)98.9/100:0.0264768722,(((HanXRQChr17_13361200_1336
1728:0.0179155134,((HanXRQChr12_75917343_75917873:0.0044251049,HanXRQChr14_863
41928_86342457:0.0000022540)92.3/100:0.0136426192,HanXRQChr03_99988684_99989213
:0.0320501782)74/100:0.0042732247)77.2/100:0.0044927472,HanXRQChr17_99228227_9922
8757:0.0179781112)84.2/100:0.0080946430,(HanXRQChr16_15638745_15639274:0.0088837
181,HanXRQChr05_144936503_144937032:0.0000021245)79.6/100:0.0052420398)53.3/92:0.
0044334374)0/51:0.0000024659,HanXRQChr02_114840517_114841046:0.0280183585)99.9/1
00:0.0920744856)71.7/55:0.0097374279,(((HanXRQChr11_55711325_55711859:0.103616383
0,(HanXRQChr01_29157382_29157877:0.1270085974,HanXRQChr13_104941607_10494213
7:0.1327716266)78.8/98:0.0273764349)82.6/73:0.0197088495,(HanXRQChr08_25571094_255
71648:0.0165815174,HanXRQChr05_44360152_44360708:0.0413347122)100/100:0.1095562
818)82.4/66:0.0132827505,HanXRQChr10_187118315_187118736:0.1578863758)70.3/53:0.0
068290451)90.2/56:0.0230633935)74.7/47:0.0049984920,((HanXRQChr09_8264746_8265314:
0.0267794239,HanXRQChr16_71647538_71648109:0.0155600373)98.2/100:0.0446129554,(H
anXRQChr13_88688183_88688669:0.0918438905,HanXRQChr17_31920748_31921235:0.021

4122568)100/100:0.1324592474)84.3/77:0.0136990889)0/42:0.0000027053,((HanXRQChr17_84099735_84100241:0.0654948828,HanXRQChr10_11267406_11267923:0.1010578275)88.5/99:0.0210928228,(HanXRQChr02_131243878_131244246:0.0000022540,HanXRQChr10_168209872_168210240:0.0044479397)99.9/100:0.0793585929)80.9/84:0.0191125572)93.2/57:0.0533691298,((HanXRQChr08_113839738_113840105:0.0182427317,HanXRQChr04_28158629_28159233:0.0044903456)74.1/96:0.0043775034,HanXRQChr11_39830792_39831397:0.0046755489)100/100:0.1574923007)95.6/90:0.0593800758,(((HanXRQChr11_133792609_133792984:0.0000022540,HanXRQChr11_133775764_133776143:0.0000022540)98.3/100:0.0356648359,HanXRQChr05_62558786_62559318:0.0255374042)87.5/100:0.0222610765,(HanXRQChr09_124910897_124911250:0.0049736748,HanXRQChr11_30817527_30817892:0.0174895587)100/100:0.1459963250)95.1/100:0.0404609093)84.9/64:0.0230554019,(HanXRQChr17_68193643_68194142:0.0273517059,HanXRQChr11_94539491_94539990:0.0136734102)100/100:0.1192951574)87.4/80:0.0246235482,(((((HanXRQChr01_40849696_40850225:0.0328901078,HanXRQChr11_135844467_135845030:0.0130556169)0/90:0.0000028799,(HanXRQChr16_45835607_45836135:0.0173346340,HanXRQChr04_141436125_141436654:0.0188610248)85/100:0.0176880864)99.6/100:0.0873137175,HanXRQChr14_73178118_73178607:0.1881823720)77.2/79:0.0184236781,(HanXRQChr09_74883466_74883949:0.0209344902,HanXRQChr05_179907532_179908035:0.0343140605)99.9/100:0.1144674901)48.8/41:0.0115417829,(HanXRQChr10_170724767_170725228:0.0753475970,(HanXRQChr11_114441513_114442047:0.0459185046,HanXRQChr08_22016304_22016849:0.0675736977)84.7/98:0.0195319250)93/95:0.0212514096)0/25:0.0000025501)86.6/39:0.0116832699,(((((((((((((HanXRQChr17_93185904_93186267:0.0044955139,HanXRQChr17_93126497_93127118:0.0090201873)0/56:0.0000023699,HanXRQChr17_93164704_93165330:0.0183070709)98/100:0.0225810461,(((HanXRQChr04_162198911_162199522:0.0044845072,HanXRQChr17_31095021_31095488:0.0092138861)97/100:0.0182335286,(HanXRQChr16_139484437_139485058:0.0135560757,((HanXRQChr06_73271993_73272461:0.0136972952,(HanXRQChr17_13434430_13434837:0.0183658005,(HanXRQChr05_171432714_171433335:0.0089761683,HanXRQChr02_40423347_40423968:0.0136707982)0/87:0.0000022540)96.2/100:0.0137083062)0/76:0.0000024478,HanXRQChr10_144765081_144765648:0.0912559488)0/93:0.0000024494)0/60:0.0000026627)84.9/89:0.0048498605,(((((HanXRQChr13_193729788_193730410:0.0045179667,HanXRQChr02_111025526_111026149:0.0044795217)84.7/100:0.0089848261,HanXRQChr01_83910798_83911405:0.0321694581)0/93:0.0000024451,(HanXRQChr14_28912143_28912767:0.0376907500,HanXRQChr11_61241453_61242074:0.0468962180)0/48:0.0000021342)85.2/84:0.0045431354,(HanXRQChr15_64901712_64902075:0.0000022540,HanXRQChr04_57314850_57315475:0.0000022540)97.2/100:0.0181150499)0/41:0.0000028272,HanXRQChr02_40199887_40200279:0.0229420249)83.2/82:0.0045433763)0/51:0.0000020205)0/22:0.0000020688,HanXRQChr17_139322691_139323166:0.0417252983)0/29:0.0000028171,(((HanXRQChr10_145087013_145087418:0.0184761020,HanXRQChr10_175382210_175382618:0.0137710671)88.3/100:0.0138609441,HanXRQChr06_56355680_56356069:0.0474505201)82.8/100:0.0113213652,(HanXRQChr17_95938863_95939232:0.0260756578,HanXRQChr13_144225259_144225863:0.0158742304)98.5/99:0.0467341455)77.4/93:0.0068584296)24.4/32:0.0054899213,HanXRQChr07_94174100_94174724:0.0171908304)100/100:0.1522564401,((((HanXRQChr15_52286990_52287513:0.1073539684,(HanXRQChr09_19029933_19030470:0.0000026729,HanXRQChr09_126494259_126494796:0.0045140210)85.9/100:0.0096459080)68.5/100:0.0033236860,HanXRQChr06_41524728_

41525252:0.0263354869)99.8/100:0.0754913776,(HanXRQChr03_26626677_26627224:0.034
9395635,HanXRQChr12_100245888_100246277:0.0166097388)96.4/100:0.0357612517)0/86:
0.0000023219,HanXRQChr14_47500657_47501050:0.2182189309)58.7/96:0.0169663155)88.
8/92:0.0311858915,((((((((HanXRQChr14_79508506_79508881:0.0000024012,HanXRQChr15
_145112060_145112588:0.0090655036)85.5/99:0.0090235320,HanXRQChr09_159439793_15
9440170:0.0277548988)75.4/100:0.0045484057,HanXRQChr14_45570798_45571311:0.02367
51792)0/55:0.0000020528,(HanXRQChr16_170010412_170010812:0.0276880614,HanXRQCh
r01_83270623_83271135:0.0183163890)76/100:0.0044326046)87.5/100:0.0096161891,HanX
RQChr10_173822886_173823254:0.0691609220)75.7/77:0.0041009727,HanXRQChr04_1471
40553_147141079:0.0091228307)80.8/75:0.0046503566,((HanXRQChr09_59706805_5970718
1:0.0262674379,HanXRQChr04_65301484_65302016:0.0426551309)77.3/94:0.0057129348,H
anXRQChr07_35619467_35619941:0.0704006968)78.8/85:0.0049505482)0/46:0.0000027258,
HanXRQChr02_46053364_46053773:0.0136005381)0/59:0.0015437312,HanXRQChr10_1005
32038_100532438:0.0355808226)99/100:0.0747574124)3.4/53:0.0051333114,(HanXRQChr15
_125481128_125481695:0.0219796201,HanXRQChr10_140012131_140012699:0.005704625
7)100/100:0.1587220046)89.4/93:0.0314614405,(HanXRQChr15_153093160_153093558:0.12
93605385,HanXRQChr11_77517539_77518062:0.0923525484)67.3/94:0.0334935717)56/85:0.
0104067335,(HanXRQChr09_48002641_48003124:0.0000021258,HanXRQChr09_48019973_
48020456:0.0000021295)100/100:0.1654147743)79.8/24:0.0118296754,(HanXRQChr17_5490
7212_54907721:0.0285151925,HanXRQChr09_50143993_50144471:0.0364514983)100/100:0
.0993172491)0/16:0.0000023416,HanXRQChr07_67769_68326:0.0950108438)22.7/17:0.0085
363584)62.1/48:0.0088919563,HanXRQChr10_170627841_170628410:0.1055830416)42.2/53:
0.0578054828,HanXRQChr13_127794694_127795300:0.1396943199)100/100:1.5722832740)
65.6/89:0.0373179738,HanXRQChr12_94877770_94878735:0.1681877680)85.8/89:0.0446339
743)100/100:0.5132205684)54.5/93:0.0861661072)91.2/94:0.0720470808)19.7/50:0.01139462
64,((((((((((((HanXRQChr16_150636591_150640613:0.0224030781,(((HanXRQChr17_476376
09_47642782:0.0129046546,HanXRQChr09_42030904_42038738:0.0232429814)0/92:0.0000
022540,HanXRQChr08_88220498_88221890:0.0042367155)86.6/100:0.0083781888,HanXRQ
Chr15_74394306_74402182:0.0176679577)89.6/100:0.0133431160)93.4/100:0.0234694656,H
anXRQChr05_177102059_177102727:0.0081955880)93.6/98:0.0156506186,HanXRQChr05_1
99634091_199642069:0.0042841688)74/98:0.0040548450,HanXRQChr09_26818251_268207
77:0.0304890772)95.2/99:0.0204690079,HanXRQChr03_34702985_34703845:0.0400788524)
4.2/70:0.0037074249,((HanXRQChr17_164543226_164545213:0.0040702735,(HanXRQChr11
_5059413_5062302:0.0000022540,HanXRQChr16_159464039_159466946:0.0000022540)96.
6/100:0.0122258768)0/93:0.0000024653,((HanXRQChr04_4585394_4586317:0.0131102746,H
anXRQChr12_8060455_8064354:0.0000022540)72/100:0.0043231239,HanXRQChr04_441427
3_4418183:0.0086156592)100/100:0.0557133123)96.9/100:0.0205743286)85.3/97:0.01547181
02,(((HanXRQChr16_154231921_154234094:0.0439071168,(HanXRQChr10_201208107_201
211833:0.0167495056,HanXRQChr06_1335657_1337253:0.0350861332)57.7/100:0.00573331
71)87.4/100:0.0267520050,((HanXRQChr09_1135754_1136217:0.0045446099,HanXRQChr09
_1132214_1133064:0.0084856339)99.7/100:0.1178564020,(HanXRQChr14_171048254_1710
55488:0.0682357619,HanXRQChr02_147305501_147307299:0.0617559034)98.7/100:0.10994
54134)97.3/99:0.0762847599)82.4/95:0.0167650395,(HanXRQChr06_47763415_47764638:0.0
362908481,HanXRQChr12_40589262_40592252:0.0415747948)97.9/100:0.0426325808)81.3/

96:0.0114053829)85.8/100:0.0191539633,HanXRQChr05_52352508_52353847:0.0508567800
)41.5/96:0.0222784984,HanXRQChr12_66452661_66454107:0.0644229184)86.9/100:0.01955
58591,HanXRQChr03_153476779_153477741:0.0639975744)99.8/100:0.0674289736,HanXR
QChr09_192317181_192318492:0.0561199991)80/99:0.0132850951,HanXRQChr09_1736863
90_173688466:0.1039845614)19.5/86:0.0072713624,(HanXRQChr07_27412393_27412825:0.
0085861973,HanXRQChr07_27480885_27481317:0.0000021543)100/100:0.4699980741)81.3/
79:0.0239801038)88.8/95:0.0337404761,((HanXRQChr10_167996501_167997306:0.03755533
39,(HanXRQChr07_94263537_94264736:0.0149427312,((((HanXRQChr10_200122501_20012
6511:0.0083095534,HanXRQChr03_159773788_159776353:0.0084343562)87.8/100:0.008964
4963,HanXRQChr16_117885695_117887257:0.0102784898)95.2/100:0.0201931407,HanXRQ
Chr16_139387141_139394695:0.0079291516)0/85:0.0000020544,HanXRQChr13_70292420_
70293133:0.0229518795)97/100:0.0375826281)98.8/100:0.0684944733)99.5/100:0.096754148
1,((((((HanXRQChr12_74630615_74638291:0.0302273622,(HanXRQChr05_32799874_328060
12:0.0176544566,HanXRQChr05_66536036_66536958:0.0223113350)78.8/100:0.0061780324
)97.9/100:0.0472058129,(HanXRQChr09_42108185_42108601:0.0219886635,((HanXRQChr1
4_70675600_70678361:0.0043355838,(HanXRQChr14_79208902_79211665:0.0130582144,H
anXRQChr14_77501682_77504443:0.0086855543)0/91:0.0000022540)0/99:0.0000028393,Ha
nXRQChr02_175774663_175777915:0.0174311311)73.9/100:0.0043604256)74/100:0.0057683
475)71.6/99:0.0454337796,HanXRQChr08_40489307_40491198:0.0723470310)99.1/100:0.07
57246341,(HanXRQChr09_184856661_184857251:0.0289399131,HanXRQChr13_144823475
_144824100:0.0244574424)99.8/100:0.0925866405)69.5/81:0.0254972110,(HanXRQChr15_10
4285615_104286058:0.0516518404,HanXRQChr05_209135841_209137273:0.0760565209)94
.7/100:0.0449303399)75.4/57:0.0124000662,HanXRQChr16_128055830_128059471:0.200996
9378)27.1/63:0.0283375352)99.6/100:0.0960555791)95.1/100:0.0612855441)98.8/99:0.051750
1134)0/93:0.0000020038,HanXRQChr11_34832223_34833041:0.0338048647)40.9/98:0.00204
77769,HanXRQChr03_69429483_69433013:0.0156264501)82.6/100:0.0116980942)0/55:0.000
0024175)90.5/100:0.0139448863,HanXRQChr04_162448231_162451209:0.0313534038)97.2/
100:0.0322695723)89.9/100:0.0231658279,(HanXRQChr13_159695079_159696066:0.017694
6890,HanXRQChr06_34593592_34601130:0.0086111503)89.8/100:0.0193474461)95.9/100:0.
0351968199,((((((HanXRQChr07_27634064_27638140:0.0000022540,HanXRQChr05_644166
93_64422326:0.0086796800)0/99:0.0000021252,(HanXRQChr15_23210956_23213675:0.0043
601059,HanXRQChr11_69846093_69853687:0.0043633653)90.5/99:0.0087291819)18.2/85:0.
0042935285,(HanXRQChr06_20451348_20451704:0.0000022540,HanXRQChr13_179591469
_179595652:0.0087323290)0/97:0.0000021158)91.6/98:0.0086662397,(((HanXRQChr12_4590
5678_45909951:0.0000022540,HanXRQChr15_74224040_74228313:0.0000022540)89.6/100:
0.0086815278,HanXRQChr13_2986278_2987762:0.0043292234)0/67:0.0000027322,HanXRQ
Chr02_4724981_4726023:0.0000022540)0/67:0.0000026213)0/80:0.0000020558,HanXRQChr
13_135223336_135224153:0.0358976352)89.8/100:0.0152587729,HanXRQChr13_196286900
_196290049:0.0117779301)96.4/100:0.0305700521)79.2/98:0.0158641778);

CONSENSUS TREE

Consensus tree is constructed from 1000bootstrap trees

Log-likelihood of consensus tree: -23855.318826
Robinson-Foulds distance between ML tree and consensus tree: 22

Branches with support >0.000000% are kept (extended consensus)
Branch lengths are optimized by maximum likelihood on original alignment
Numbers in parentheses are bootstrap supports (%)

```
+--HanXRQChr11_78963679_78964101
|
|       +--HanXRQChr01_34887426_34894282
|       +--| (92)
|       |  |  +--HanXRQChr12_24613340_24614343
|       |  +--| (95)
|       |     +--HanXRQChr08_22937900_22939039
|       +--| (98)
|       |  +--HanXRQChr13_45702842_45703840
|    +--| (98)
|    |  +--HanXRQChr17_205330461_205331450
+--| (100)
|  +--HanXRQChr03_26205754_26213308
|
|       +--HanXRQChr04_67540092_67541320
|       +--| (100)
|       |  |     +--HanXRQChr06_27984911_27988051
|       |  |  +--| (100)
|       |  |  |  |  +--HanXRQChr04_12175865_12181403
|       |  |  |  +--| (55)
|       |  |  |     |       +--HanXRQChr17_211327788_211333859
|       |  |  |     |       +--| (100)
|       |  |  |     |       |  |  +--HanXRQChr05_59284493_59290538
|       |  |  |     |       |  +--| (100)
|       |  |  |     |       |     +--HanXRQChr05_106376295_106382341
|       |  |  |     |       +--| (93)
|       |  |  |     |       |  |     +--HanXRQChr09_48889131_48889670
|       |  |  |     |       |  |  +--| (99)
|       |  |  |     |       |  |  |  +--HanXRQChr15_55516101_55518531
|       |  |  |     |       |  +--| (99)
|       |  |  |     |       |     |              +--HanXRQChr03_57480187_57481416
|       |  |  |     |       |     |       +--| (80)
|       |  |  |     |       |     |       |  |  +--HanXRQChr05_166779247_166780763
|       |  |  |     |       |     |       |  +--| (100)
|       |  |  |     |       |     |       |     +--HanXRQChr12_94875864_94876484
|       |  |  |     |       |     |       +--| (99)
|       |  |  |     |       |     |       |  |  +--HanXRQChr04_92418435_92419412
```

```
|       | | |   |       |       |       | +--| (100)
|       | | |   |       |       |       |       | +--HanXRQChr06_53262309_53263292
|       | | |   |       |       |       |       +--| (100)
|       | | |   |       |       |       |       +--HanXRQChr05_9034545_9035531
|       | | |   |       |       |       +--| (99)
|       | | |   |       |       |       | +--HanXRQChr11_113608046_113608769
|       | | |   |       |       |       +--| (50)
|       | | |   |       |       |       | |       +--HanXRQChr16_94477431_94477942
|       | | |   |       |       |       | |       +--| (96)
|       | | |   |       |       |       | |       | |       +--HanXRQChr17_67579875_67582613
|       | | |   |       |       |       | |       | |       +--| (100)
|       | | |   |       |       |       | |       | |       | +--
HanXRQChr11_142203384_142204256
|       | | |   |       |       |       | |       | |       +--| (88)
|       | | |   |       |       |       | |       | |       | +--
HanXRQChr04_176555457_176557628
|       | | |   |       |       |       | |       | | +--| (96)
|       | | |   |       |       |       | |       | | | +--HanXRQChr01_116026086_116027124
|       | | |   |       |       |       | |       | +--| (100)
|       | | |   |       |       |       | |       |       +--HanXRQChr13_137859189_137859594
|       | | |   |       |       |       | |       +--| (97)
|       | | |   |       |       |       | |       | +--HanXRQChr10_152624866_152626709
|       | | |   |       |       |       | | +--| (100)
|       | | |   |       |       |       | | | |           +--HanXRQChr07_57474618_57475394
|       | | |   |       |       |       | | | | +--| (100)
|       | | |   |       |       |       | | | | | +--HanXRQChr07_57434299_57438258
|       | | |   |       |       |       | | | | +--| (100)
|       | | |   |       |       |       | | | | | | +--HanXRQChr05_190757444_190758598
|       | | |   |       |       |       | | | | | +--| (100)
|       | | |   |       |       |       | | | | |       | +--HanXRQChr13_94278579_94280493
|       | | |   |       |       |       | | | | |       +--| (100)
|       | | |   |       |       |       | | | | |       +--HanXRQChr04_115355662_115358538
|       | | |   |       |       |       | | | +--| (98)
|       | | |   |       |       |       | | |   |       +--HanXRQChr11_18867372_18868580
|       | | |   |       |       |       | | |   |       +--| (73)
|       | | |   |       |       |       | | |   |       | +--HanXRQChr02_20281946_20283260
|       | | |   |       |       |       | | |   | +--| (100)
|       | | |   |       |       |       | | |   | | +--HanXRQChr11_18749900_18751215
|       | | |   |       |       |       | | |   +--| (98)
|       | | |   |       |       |       | | |   +--HanXRQChr06_28223014_28227854
|       | | |   |       |       |       | +--| (94)
|       | | |   |       |       |       |       |       +----HanXRQChr12_88474680_88475105
|       | | |   |       |       |       |       | +--| (99)
```

```
|       | | |   |       |       |       | | |          +--
HanXRQChr01_1207903_1208488
|       | | |   |       |       |       | | |          +--| (99)
|       | | |   |       |       |       | | |          | | +--
HanXRQChr08_151986814_151987353
|       | | |   |       |       |       | | |          | +--| (100)
|       | | |   |       |       |       | | |          |      +--
HanXRQChr10_94811711_94812246
|       | | |   |       |       |       | | |          +--| (99)
|       | | |   |       |       |       | | |          | | +--
HanXRQChr05_168861330_168861873
|       | | |   |       |       |       | | |          | +--| (100)
|       | | |   |       |       |       | | |          |      | +--
HanXRQChr05_193946694_193947239
|       | | |   |       |       |       | | |          |      +--| (100)
|       | | |   |       |       |       | | |          |      +--
HanXRQChr05_193733456_193734002
|       | | |   |       |       |       | | |   +--| (99)
|       | | |   |       |       |       | | |   | +--
HanXRQChr05_182283860_182284319
|       | | |   |       |       |       | | +-------| (100)
|       | | |   |       |       |       | |     | +--
HanXRQChr17_105500849_105501312
|       | | |   |       |       |       | |     +--| (98)
|       | | |   |       |       |       | |            |      +--
HanXRQChr04_16618909_16619279
|       | | |   |       |       |       | |            | +--| (60)
|       | | |   |       |       |       | |            | | |   +--
HanXRQChr01_146764857_146765256
|       | | |   |       |       |       | |            | | +-------| (100)
|       | | |   |       |       |       | |            | |    |      +--
HanXRQChr03_20364521_20364920
|       | | |   |       |       |       | |            | |    | +--| (43)
|       | | |   |       |       |       | |            | |    | | +--
HanXRQChr16_185568115_185568603
|       | | |   |       |       |       | |            | |    +--| (76)
|       | | |   |       |       |       | |            | |           +--
HanXRQChr05_14305976_14306375
|       | | |   |       |       |       | |            +--| (62)
|       | | |   |       |       |       | |            +--
HanXRQChr03_113315527_113315992
|       | | |   |       |       |       +--| (93)
|       | | |   |       |       |       |            +--
HanXRQChr02_175754451_175758707
```

| | | | | | | | +--| (93)
| | | | | | | | | +--
HanXRQChr13_94161763_94165162
| | | | | | | | +--| (68)
| | | | | | | | | | +--HanXRQChr11_38886892_38889248
| | | | | | | | | +--| (93)
| | | | | | | | | | +--
HanXRQChr05_164509744_164514326
| | | | | | | | | +--| (82)
| | | | | | | | | +--
HanXRQChr03_34772558_34773468
| | | | | | | | +--| (91)
| | | | | | | | | | +--
HanXRQChr08_33398524_33406006
| | | | | | | | | | +--| (100)
| | | | | | | | | | | +--
HanXRQChr12_40586043_40589256
| | | | | | | | | | +--| (85)
| | | | | | | | | | | | +--
HanXRQChr13_77076783_77077636
| | | | | | | | | | | +--| (100)
| | | | | | | | | | | | +--
HanXRQChr10_115966867_115967362
| | | | | | | | | | | | +--| (34)
| | | | | | | | | | | | | +--
HanXRQChr14_68245992_68247226
| | | | | | | | | | | | +--| (47)
| | | | | | | | | | | | | +--
HanXRQChr01_49628126_49635945
| | | | | | | | | | | | +--| (45)
| | | | | | | | | | | | | | +--
HanXRQChr09_125961449_125964480
| | | | | | | | | | | | | +--| (70)
| | | | | | | | | | | | | | +--
HanXRQChr06_28014629_28015962
| | | | | | | | | | | | | | +--| (100)
| | | | | | | | | | | | | | | +--
HanXRQChr11_23461540_23465141
| | | | | | | | | | | | | +--| (76)
| | | | | | | | | | | | | +--
HanXRQChr04_166379772_166382442
| | | | | | | | | | | | +--| (76)
| | | | | | | | | | | | | +--
HanXRQChr17_164520103_164521773

```
|         | | |    |         |         |         |         | |       |         +--| (99)
|         | | |    |         |         |         |         | |       |         |         +--
HanXRQChr04_174582120_174583103
|         | | |    |         |         |         |         | |       |         |  +--| (99)
|         | | |    |         |         |         |         | |       |         | |  +--
HanXRQChr13_135224556_135226019
|         | | |    |         |         |         |         | |       |         +--| (78)
|         | | |    |         |         |         |         | |       |         |  +--
HanXRQChr07_11945240_11945839
|         | | |    |         |         |         |         | |       |         +--| (100)
|         | | |    |         |         |         |         | |       |         +--
HanXRQChr05_23977914_23979210
|         | | |    |         |         |         |         | |  +--| (99)
|         | | |    |         |         |         |         | | |  +--
HanXRQChr03_50693386_50694309
|         | | |    |         |         |         |         |  +--| (98)
|         | | |    |         |         |         |         |         +--
HanXRQChr08_40474325_40480066
|         | | |    |         |         |         |         +--| (85)
|         | | |    |         |         |         |         | |               +--
HanXRQChr02_24653810_24657437
|         | | |    |         |         |         |         | |               +--| (100)
|         | | |    |         |         |         |         | |               | |  +--
HanXRQChr14_68289895_68297518
|         | | |    |         |         |         |         | |               |  +--| (100)
|         | | |    |         |         |         |         | |               |         +--
HanXRQChr14_68273516_68281146
|         | | |    |         |         |         |         | |               +--| (90)
|         | | |    |         |         |         |         | |               | |                 +--
HanXRQChr02_147295547_147297741
|         | | |    |         |         |         |         | |               | |                 +--| (100)
|         | | |    |         |         |         |         | |               | |                 |  +--
HanXRQChr09_142942803_142944567
|         | | |    |         |         |         |         | |               | |       +--| (100)
|         | | |    |         |         |         |         | |               | |       |  +--
HanXRQChr13_96620660_96621156
|         | | |    |         |         |         |         | |               | |       +--| (100)
|         | | |    |         |         |         |         | |               | |       |  +--
HanXRQChr04_132271481_132272204
|         | | |    |         |         |         |         | |               | |       +--| (100)
|         | | |    |         |         |         |         | |               | |       |  +--
HanXRQChr14_153324342_153328229
|         | | |    |         |         |         |         | |               | |  +--| (100)
```

```
|         | | |    |         |         |         |         | |               | | |  +--
HanXRQChr10_85192350_85197513
|         | | |    |         |         |         |         | |               |  +--| (91)
|         | | |    |         |         |         |         | |               |         |         +--
HanXRQChr06_97006962_97010930
|         | | |    |         |         |         |         | |               |         |  +--| (100)
|         | | |    |         |         |         |         | |               |         | |  +--
HanXRQChr03_159793635_159795071
|         | | |    |         |         |         |         | |               |         +--| (100)
|         | | |    |         |         |         |         | |               |         |         +--
HanXRQChr07_88885423_88893193
|         | | |    |         |         |         |         | |               |         |         +--| (47)
|         | | |    |         |         |         |         | |               |         |         |  +--
HanXRQChr08_109696851_109697593
|         | | |    |         |         |         |         | |               |         |  +--| (100)
|         | | |    |         |         |         |         | |               |         | |  +--
HanXRQChr08_39439464_39440150
|         | | |    |         |         |         |         | |               |         +--| (95)
|         | | |    |         |         |         |         | |               |         |  +--
HanXRQChr01_140346486_140347133
|         | | |    |         |         |         |         | |               |         +--| (99)
|         | | |    |         |         |         |         | |               |         +--
HanXRQChr16_117883570_117885696
|         | | |    |         |         |         |         | |               +--| (90)
|         | | |    |         |         |         |         | |               |  +--
HanXRQChr10_225800164_225801136
|         | | |    |         |         |         |         | |      +--| (82)
|         | | |    |         |         |         |         | |      | |       +--
HanXRQChr04_79257170_79260978
|         | | |    |         |         |         |         | |      | |  +--| (100)
|         | | |    |         |         |         |         | |      | | | |  +--
HanXRQChr09_141496442_141499885
|         | | |    |         |         |         |         | |      | | |  +--| (85)
|         | | |    |         |         |         |         | |      | | |     +--
HanXRQChr03_70542853_70547885
|         | | |    |         |         |         |         | |      |  +--| (88)
|         | | |    |         |         |         |         | |      |         +--
HanXRQChr07_94262118_94263107
|         | | |    |         |         |         |         | |      +--| (99)
|         | | |    |         |         |         |         | |      | |       +--
HanXRQChr17_170737481_170744938
|         | | |    |         |         |         |         | |      | |       +--| (88)
|         | | |    |         |         |         |         | |      | |       |  +--
HanXRQChr04_115339901_115344808
```

```
|         | | |    |         |         |         |         | |       | |       +--| (100)
|         | | |    |         |         |         |         | |       | |       |  +--
HanXRQChr05_177667685_177675472
|         | | |    |         |         |         |         | |       | |  +--| (100)
|         | | |    |         |         |         |         | |       | | | |                 +--
HanXRQChr13_179578065_179585597
|         | | |    |         |         |         |         | |       | | | |                 +--| (100)
|         | | |    |         |         |         |         | |       | | | |                 |  +--
HanXRQChr13_179805528_179808054
|         | | |    |         |         |         |         | |       | | | |         +--| (56)
|         | | |    |         |         |         |         | |       | | | |         | |  +--
HanXRQChr05_64422906_64424659
|         | | |    |         |         |         |         | |       | | | |         |  +--| (99)
|         | | |    |         |         |         |         | |       | | | |         |         +--
HanXRQChr13_7315803_7323380
|         | | |    |         |         |         |         | |       | | | |         +--| (61)
|         | | |    |         |         |         |         | |       | | | |         |  +--
HanXRQChr15_23213675_23214340
|         | | |    |         |         |         |         | |       | | | |         +--| (46)
|         | | |    |         |         |         |         | |       | | | |         | |  +--
HanXRQChr11_127530325_127537865
|         | | |    |         |         |         |         | |       | | | |         |  +--| (67)
|         | | |    |         |         |         |         | |       | | | |         |         +--
HanXRQChr12_23644236_23645297
|         | | |    |         |         |         |         | |       | | | |         +--| (92)
|         | | |    |         |         |         |         | |       | | | |         | |  +--
HanXRQChr14_116526279_116533821
|         | | |    |         |         |         |         | |       | | | |         |  +--| (75)
|         | | |    |         |         |         |         | |       | | | |         |         +--
HanXRQChr17_205323932_205330231
|         | | |    |         |         |         |         | |       | | | |  +--| (90)
|         | | |    |         |         |         |         | |       | | | | | |  +--
HanXRQChr17_3057459_3059571
|         | | |    |         |         |         |         | |       | | | | |  +--| (88)
|         | | |    |         |         |         |         | |       | | | | |         |         +--
HanXRQChr13_169241697_169244853
|         | | |    |         |         |         |         | |       | | | | |         |  +--| (68)
|         | | |    |         |         |         |         | |       | | | | |         | |  +--
HanXRQChr01_90452614_90460077
|         | | |    |         |         |         |         | |       | | | | |         +--| (100)
|         | | |    |         |         |         |         | |       | | | | |         |  +--
HanXRQChr13_45704069_45710378
|         | | |    |         |         |         |         | |       | | | | |         +--| (100)
```

| | | | | | | | | | | | | | | | | +--
HanXRQChr08_22931465_22937801
| | | | | | | | | | | | | | | +--| (100)
| | | | | | | | | | | | | | | | +--
HanXRQChr13_196274607_196275703
| | | | | | | | | | | | | | +--| (94)
| | | | | | | | | | | | | | | +--
HanXRQChr13_48436772_48438877
| | | | | | | | | | | | | | | +--| (89)
| | | | | | | | | | | | | | | | | +--
HanXRQChr15_129312466_129317224
| | | | | | | | | | | | | | | | | +--| (57)
| | | | | | | | | | | | | | | | | | +--
HanXRQChr02_147809574_147817424
| | | | | | | | | | | | | | | | | +--| (89)
| | | | | | | | | | | | | | | | | | | +--
HanXRQChr14_171031165_171032099
| | | | | | | | | | | | | | | | | | +--| (100)
| | | | | | | | | | | | | | | | | | +--
HanXRQChr10_113962499_113970363
| | | | | | | | | | | | | | | | +--| (81)
| | | | | | | | | | | | | | | | | +--
HanXRQChr11_23755532_23757425
| | | | | | | | | | | | | | | | +--| (67)
| | | | | | | | | | | | | | | | +--
HanXRQChr01_4643702_4650088
| | | | | | | | | | | | | | +--| (97)
| | | | | | | | | | | | | | +--
HanXRQChr08_88222000_88228288
| | | | | | | | | | | | +--| (91)
| | | | | | | | | | | | +--
HanXRQChr15_150283897_150291604
| | | | | | | | | | | +--| (99)
| | | | | | | | | | | | | +--
HanXRQChr03_46527653_46534444
| | | | | | | | | | | | | +--| (100)
| | | | | | | | | | | | | | +--
HanXRQChr06_51391635_51395795
| | | | | | | | | | | | +--| (100)
| | | | | | | | | | | | +--
HanXRQChr07_44435735_44436200
| | | | | | | | | | | +--| (70)
| | | | | | | | | | | | +--
HanXRQChr10_167884487_167885281

```
|         | | |    |         |         |         |         |  +--| (75)
|         | | |    |         |         |         |         |         |         +--
HanXRQChr16_149753884_149754258
|         | | |    |         |         |         |         |         |         +--| (78)
|         | | |    |         |         |         |         |         |         |  +--
HanXRQChr01_98528860_98530430
|         | | |    |         |         |         |         |         |  +--| (69)
|         | | |    |         |         |         |         |         | |  +--
HanXRQChr16_128066409_128067498
|         | | |    |         |         |         |         |         +--| (72)
|         | | |    |         |         |         |         |         |         +--
HanXRQChr02_20242486_20243525
|         | | |    |         |         |         |         |         |  +--| (100)
|         | | |    |         |         |         |         |         | |  +--
HanXRQChr11_18762949_18764264
|         | | |    |         |         |         |         |         +--| (100)
|         | | |    |         |         |         |         |         +--
HanXRQChr16_148694596_148701069
|         | | |    |         |         |         +---------| (100)
|         | | |    |         |         |                   |         +--
HanXRQChr15_23293519_23294345
|         | | |    |         |         |                   |         +--| (79)
|         | | |    |         |         |                   |         | |       +--
HanXRQChr06_29856663_29857319
|         | | |    |         |         |                   |         |  +-----| (100)
|         | | |    |         |         |                   |         |         +--------------
HanXRQChr17_60426850_60428287
|         | | |    |         |         |                   |  +--| (89)
|         | | |    |         |         |                   | | |
          +--HanXRQChr07_30137334_30143115
|         | | |    |         |         |                   | | |
          +--| (87)
|         | | |    |         |         |                   | | |
          |  +--HanXRQChr15_6435740_6441546
|         | | |    |         |         |                   | | |
          +--| (100)
|         | | |    |         |         |                   | | |
          |  +--HanXRQChr05_211734084_211734739
|         | | |    |         |         |                   | | |
          +--| (67)
|         | | |    |         |         |                   | | |
          |  +--HanXRQChr04_4816946_4817774
|         | | |    |         |         |                   | | |
          +--| (100)
```

```
|       | | |   |       |       |       |               | | |
        | +--HanXRQChr09_90613212_90613775
|       | | |   |       |       |       |               | | |
        +--| (97)
|       | | |   |       |       |       |               | | |
        | |     +--HanXRQChr14_164972043_164972420
|       | | |   |       |       |       |               | | |
        | |  +--| (100)
|       | | |   |       |       |       |               | | |
        | | | | +--HanXRQChr17_171676962_171677479
|       | | |   |       |       |       |               | | |
        | | |  +--| (100)
|       | | |   |       |       |       |               | | |
        | | |   | +--HanXRQChr04_11014761_11015279
|       | | |   |       |       |       |               | | |
        | | |   +--| (100)
|       | | |   |       |       |       |               | | |
        | | |   +--HanXRQChr04_11235868_11236386
|       | | |   |       |       |       |               | | |
        | +--| (100)
|       | | |   |       |       |       |               | | |
        |       +--HanXRQChr10_36325499_36326037
|       | | |   |       |       |       |               | | |
        +--| (69)
|       | | |   |       |       |       |               | | |
        | | +--HanXRQChr09_16583017_16583476
|       | | |   |       |       |       |               | | |
        | +--| (100)
|       | | |   |       |       |       |               | | |
        |       +--HanXRQChr02_53194187_53194755
|       | | |   |       |       |       |               | | |
        +--| (52)
|       | | |   |       |       |       |               | | |
        | +--HanXRQChr15_58262815_58263268
|       | | |   |       |       |       |               | | |
        +--| (37)
|       | | |   |       |       |       |               | | |
        | |             +--HanXRQChr03_53881858_53882406
|       | | |   |       |       |       |               | | |
        | |             +--| (100)
|       | | |   |       |       |       |               | | |
        | |             | +--HanXRQChr15_50520164_50520709
|       | | |   |       |       |       |               | | |
        | |     +--| (100)
```

```
|         | | |    |        |        |        |                 | | |
          | |      | +--HanXRQChr08_58106590_58107151
|         | | |    |        |        |        |                 | | |
          | |      +--| (68)
|         | | |    |        |        |        |                 | | |
          | |      | +--HanXRQChr04_117734534_117735067
|         | | |    |        |        |        |                 | | |
          | |      +--| (58)
|         | | |    |        |        |        |                 | | |
          | |      | +--HanXRQChr07_39909716_39910258
|         | | |    |        |        |        |                 | | |
          | |  +--| (53)
|         | | |    |        |        |        |                 | | |
          | | |  +--HanXRQChr11_114599910_114600468
|         | | |    |        |        |        |                 | | |
          | +--| (34)
|         | | |    |        |        |        |                 | | |
          |        |        +--HanXRQChr08_102456485_102456998
|         | | |    |        |        |        |                 | | |
          |        | +--| (95)
|         | | |    |        |        |        |                 | | |
          |        | | |  +--HanXRQChr03_6367591_6368162
|         | | |    |        |        |        |                 | | |
          |        | |  +--| (95)
|         | | |    |        |        |        |                 | | |
          |        | |      | +--HanXRQChr08_32332017_32332576
|         | | |    |        |        |        |                 | | |
          |        | |      +--| (100)
|         | | |    |        |        |        |                 | | |
          |        | |      +--HanXRQChr05_142623602_142624161
|         | | |    |        |        |        |                 | | |
          |        +--| (60)
|         | | |    |        |        |        |                 | | |
          |        |        +--HanXRQChr10_160816414_160816781
|         | | |    |        |        |        |                 | | |
          |        |        +--| (99)
|         | | |    |        |        |        |                 | | |
          |        |        | +--HanXRQChr09_205724496_205724897
|         | | |    |        |        |        |                 | | |
          |        | +--| (57)
|         | | |    |        |        |        |                 | | |
          |        | |  +--HanXRQChr05_5609714_5610098
|         | | |    |        |        |        |                 | | |
          |        +--| (100)
```

| | | | | | | | | | | |
| +--HanXRQChr16_19443969_19444339
| | | | | | | | | | | |
+--| (35)
| | | | | | | | | | | |
| | +--HanXRQChr01_65087231_65087766
| | | | | | | | | | | |
| | +--| (100)
| | | | | | | | | | | |
| | | +--HanXRQChr10_26225253_26225786
| | | | | | | | | | | |
| | +--| (100)
| | | | | | | | | | | |
| | | +--HanXRQChr09_6730323_6730857
| | | | | | | | | | | |
| | +--| (98)
| | | | | | | | | | | |
| | | +--HanXRQChr04_145024358_145024790
| | | | | | | | | | | |
| +--| (96)
| | | | | | | | | | | |
| +--HanXRQChr12_103007227_103007661
| | | | | | | | | | | | +--|
(47)
| | | | | | | | | | | | | |
+--HanXRQChr08_56688339_56688696
| | | | | | | | | | | | | |
+--| (96)
| | | | | | | | | | | | | |
| | +**HanXRQChr03_150064680_150065184
	+--
		+**HanXRQChr10_93521599_93522103
	+--	(61)
		+--HanXRQChr03_126121322_126121826
+--	(100)
+--HanXRQChr10_78375584_78376088
+--| (100)

```
|       | | |   |       |       |       |              | | |                                  | |
        |  +--HanXRQChr07_12736562_12737068
|       | | |   |       |       |       |              | | |                                  | |
        +--| (100)
|       | | |   |       |       |       |              | | |                                  | |
        |  +--HanXRQChr05_164461706_164462192
|       | | |   |       |       |       |              | | |                                  | |  +--|
(55)
|       | | |   |       |       |       |              | | |                                  | | | |
        +--HanXRQChr13_134302109_134302509
|       | | |   |       |       |       |              | | |                                  | | | |
        +--| (100)
|       | | |   |       |       |       |              | | |                                  | | | |
        | |  +--HanXRQChr10_95181243_95181772
|       | | |   |       |       |       |              | | |                                  | | | |
        |  +--| (100)
|       | | |   |       |       |       |              | | |                                  | | | |
        |       +--HanXRQChr17_160614824_160615353
|       | | |   |       |       |       |              | | |                                  | | | |
+--| (51)
|       | | |   |       |       |       |              | | |                                  | | | |
| |     +--HanXRQChr17_13361200_13361728
|       | | |   |       |       |       |              | | |                                  | | | |
| |     +--| (100)
|       | | |   |       |       |       |              | | |                                  | | | |
| |     | |     +--HanXRQChr12_75917343_75917873
|       | | |   |       |       |       |              | | |                                  | | | |
| |     | |  +--| (100)
|       | | |   |       |       |       |              | | |                                  | | | |
| |     | | |  +--HanXRQChr14_86341928_86342457
|       | | |   |       |       |       |              | | |                                  | | | |
| |     |  +--| (100)
|       | | |   |       |       |       |              | | |                                  | | | |
| |     |       +--HanXRQChr03_99988684_99989213
|       | | |   |       |       |       |              | | |                                  | | | |
| |  +--| (100)
|       | | |   |       |       |       |              | | |                                  | | | |
| | |  +--HanXRQChr17_99228227_99228757
|       | | |   |       |       |       |              | | |                                  | | | |
|  +--| (92)
|       | | |   |       |       |       |              | | |                                  | | | |
|       |  +--HanXRQChr16_15638745_15639274
|       | | |   |       |       |       |              | | |                                  | | | |
|       +--| (100)
```

| | | | | | | | | | | | | | | | |
| +--HanXRQChr05_144936503_144937032
| | | | | | | | | | | | | | |
+--| (100)
| | | | | | | | | | | | | | |
+--HanXRQChr02_114840517_114841046
| | | | | | | | | | | | | +--|
(56)
| | | | | | | | | | | | |
| +--HanXRQChr11_55711325_55711859
| | | | | | | | | | | | |
| +--| (73)
| | | | | | | | | | | | |
| | | +--HanXRQChr01_29157382_29157877
| | | | | | | | | | | | |
| | +--| (98)
| | | | | | | | | | | | |
| | +--HanXRQChr13_104941607_104942137
| | | | | | | | | | | | |
| +--| (66)
| | | | | | | | | | | | |
| | | +--HanXRQChr08_25571094_25571648
| | | | | | | | | | | | |
| | +--| (100)
| | | | | | | | | | | | |
| | +--HanXRQChr05_44360152_44360708
| | | | | | | | | | | | |
+--| (53)
| | | | | | | | | | | | |
+--HanXRQChr10_187118315_187118736
| | | | | | | | | | | | +--|
(42)
| | | | | | | | | | | | | |
+--HanXRQChr09_8264746_8265314
| | | | | | | | | | | | | | +--|
(100)
| | | | | | | | | | | | | | |
+--HanXRQChr16_71647538_71648109
| | | | | | | | | | | | | +--|
(77)
| | | | | | | | | | | | |
| +--HanXRQChr13_88688183_88688669
| | | | | | | | | | | | |
+--| (100)

| | | | | | | | | | | |
+--HanXRQChr17_31920748_31921235
| | | | | | | | | | | +--|
(57)
| | | | | | | | | | | | |
+--HanXRQChr17_84099735_84100241
| | | | | | | | | | | | | +--|
(99)
| | | | | | | | | | | | | |
+--HanXRQChr10_11267406_11267923
| | | | | | | | | | | | +--|
(84)
| | | | | | | | | | | |
| +--HanXRQChr02_131243878_131244246
| | | | | | | | | | | |
+--| (100)
| | | | | | | | | | | |
+--HanXRQChr10_168209872_168210240
| | | | | | | | | | | +--|
(90)
| | | | | | | | | | | | |
+--HanXRQChr08_113839738_113840105
| | | | | | | | | | | | | +--|
(96)
| | | | | | | | | | | | | |
+--HanXRQChr04_28158629_28159233
| | | | | | | | | | | | +--|
(100)
| | | | | | | | | | | |
+--HanXRQChr11_39830792_39831397
| | | | | | | | | | +--| (64)
| | | | | | | | | | | | +--
HanXRQChr11_133792609_133792984
| | | | | | | | | | | | +--|
(100)
| | | | | | | | | | | | | +--
HanXRQChr11_133775764_133776143
| | | | | | | | | | | | +--| (100)
| | | | | | | | | | | | | +--
HanXRQChr05_62558786_62559318
| | | | | | | | | | | +--| (100)
| | | | | | | | | | | | +--
HanXRQChr09_124910897_124911250

| | | | | | | | | | | | +--|
(100)
| | | | | | | | | | | | +--
HanXRQChr11_30817527_30817892
| | | | | | | | | | | +--| (80)
| | | | | | | | | | | | | +--
HanXRQChr17_68193643_68194142
| | | | | | | | | | | | +--| (100)
| | | | | | | | | | | | +--
HanXRQChr11_94539491_94539990
| | | | | | | | | | | +--| (39)
| | | | | | | | | | | | |
+--HanXRQChr01_40849696_40850225
| | | | | | | | | | | | | +--|
(90)
| | | | | | | | | | | | | | +--
HanXRQChr11_135844467_135845030
| | | | | | | | | | | | | +--|
(100)
| | | | | | | | | | | | | | | +--
HanXRQChr16_45835607_45836135
| | | | | | | | | | | | | | +--|
(100)
| | | | | | | | | | | | | |
+--HanXRQChr04_141436125_141436654
| | | | | | | | | | | | | +--|
(79)
| | | | | | | | | | | | | | +--
HanXRQChr14_73178118_73178607
| | | | | | | | | | | | | +--| (41)
| | | | | | | | | | | | | | | +--
HanXRQChr09_74883466_74883949
| | | | | | | | | | | | | | +--|
(100)
| | | | | | | | | | | | | | +--
HanXRQChr05_179907532_179908035
| | | | | | | | | | | | +--| (25)
| | | | | | | | | | | | | +--
HanXRQChr10_170724767_170725228
| | | | | | | | | | | | +--|
(95)
| | | | | | | | | | | | | +--
HanXRQChr11_114441513_114442047

| | | | | | | | | | | | +--|
(98)
| | | | | | | | | | | | +--
HanXRQChr08_22016304_22016849
| | | | | | | | | | | +--| (34)
| | | | | | | | | | | | | +--
HanXRQChr09_48002641_48003124
| | | | | | | | | | | | +--| (100)
| | | | | | | | | | | | +--
HanXRQChr09_48019973_48020456
| | | | | | | | | | | +--| (48)
| | | | | | | | | | | | |
+--HanXRQChr17_93185904_93186267
| | | | | | | | | | | | |
+--| (56)
| | | | | | | | | | | | |
| +--HanXRQChr17_93126497_93127118
| | | | | | | | | | | | |
+--| (100)
| | | | | | | | | | | | |
| +--HanXRQChr17_93164704_93165330
| | | | | | | | | | | | | +--|
(28)
| | | | | | | | | | | | | | |
+--HanXRQChr04_162198911_162199522
| | | | | | | | | | | | | | |
+--| (100)
| | | | | | | | | | | | | | |
| +--HanXRQChr17_31095021_31095488
| | | | | | | | | | | | | | |
+--| (89)
| | | | | | | | | | | | | | |
| | +--HanXRQChr16_139484437_139485058
| | | | | | | | | | | | | | |
| +--| (60)
| | | | | | | | | | | | | | |
| | +--HanXRQChr06_73271993_73272461
| | | | | | | | | | | | | | |
| | +--| (76)
| | | | | | | | | | | | | | |
| | | | +--HanXRQChr17_13434430_13434837
| | | | | | | | | | | | | | |
| | | +--| (100)

```
|         | | |    |         |         |         |                  | | |                  | |                | |
          |         | |       | +--HanXRQChr05_171432714_171433335
|         | | |    |         |         |         |                  | | |                  | |                | |
          |         | |       +--| (87)
|         | | |    |         |         |         |                  | | |                  | |                | |
          |         | |       +--HanXRQChr02_40423347_40423968
|         | | |    |         |         |         |                  | | |                  | |                | |
          |         +--| (93)
|         | | |    |         |         |         |                  | | |                  | |                | |
          |         +--HanXRQChr10_144765081_144765648
|         | | |    |         |         |         |                  | | |                  | |                | | +--|
(51)
|         | | |    |         |         |         |                  | | |                  | |                | | | |
          +--HanXRQChr13_193729788_193730410
|         | | |    |         |         |         |                  | | |                  | |                | | | |
          +--| (100)
|         | | |    |         |         |         |                  | | |                  | |                | | | |
          | +--HanXRQChr02_111025526_111026149
|         | | |    |         |         |         |                  | | |                  | |                | | | |
          +--| (93)
|         | | |    |         |         |         |                  | | |                  | |                | | | |
          | +--HanXRQChr01_83910798_83911405
|         | | |    |         |         |         |                  | | |                  | |                | | | |
          +--| (84)
|         | | |    |         |         |         |                  | | |                  | |                | | | |
          | | +--HanXRQChr14_28912143_28912767
|         | | |    |         |         |         |                  | | |                  | |                | | | |
          | +--| (48)
|         | | |    |         |         |         |                  | | |                  | |                | | | |
          |         +--HanXRQChr11_61241453_61242074
|         | | |    |         |         |         |                  | | |                  | |                | | | |
+--| (41)
|         | | |    |         |         |         |                  | | |                  | |                | | | |
| | +--HanXRQChr15_64901712_64902075
|         | | |    |         |         |         |                  | | |                  | |                | | | |
| +--| (100)
|         | | |    |         |         |         |                  | | |                  | |                | | | |
|         +--HanXRQChr04_57314850_57315475
|         | | |    |         |         |         |                  | | |                  | |                | | |
+--| (82)
|         | | |    |         |         |         |                  | | |                  | |                | | |
          +--HanXRQChr02_40199887_40200279
|         | | |    |         |         |         |                  | | |                  | |                | +--|
(35)
```

| | | | | | | | | | | | | |
+--HanXRQChr07_94174100_94174724
| | | | | | | | | | | | | +--|
(63)
| | | | | | | | | | | | | | +--
HanXRQChr17_139322691_139323166
| | | | | | | | | | | | | +--|
(100)
| | | | | | | | | | | | | | |
+--HanXRQChr10_145087013_145087418
| | | | | | | | | | | | | | |
+--| (100)
| | | | | | | | | | | | | | |
| +--HanXRQChr10_175382210_175382618
| | | | | | | | | | | | | | | +--|
(100)
| | | | | | | | | | | | | | | |
+--HanXRQChr06_56355680_56356069
| | | | | | | | | | | | | | +--|
(93)
| | | | | | | | | | | | | |
| +--HanXRQChr17_95938863_95939232
| | | | | | | | | | | | | |
+--| (99)
| | | | | | | | | | | | | |
+--HanXRQChr13_144225259_144225863
| | | | | | | | | | | | | +--|
(92)
| | | | | | | | | | | | | | |
+--HanXRQChr15_52286990_52287513
| | | | | | | | | | | | | | |
+--| (100)
| | | | | | | | | | | | | | |
| | +--HanXRQChr09_19029933_19030470
| | | | | | | | | | | | | | |
| +--| (100)
| | | | | | | | | | | | | | |
| +--HanXRQChr09_126494259_126494796
| | | | | | | | | | | | | | |
+--| (100)
| | | | | | | | | | | | | | |
| +--HanXRQChr06_41524728_41525252
| | | | | | | | | | | | | | | +--|
(86)

| | | | | | | | | | | | | | | | |
+--HanXRQChr03_26626677_26627224
| | | | | | | | | | | | | | | |
+--| (100)
| | | | | | | | | | | | | | | |
+--HanXRQChr12_100245888_100246277
| | | | | | | | | | | | | | +--|
(96)
| | | | | | | | | | | | | |
+--HanXRQChr14_47500657_47501050
| | | | | | | | | | | | | +--| (53)
| | | | | | | | | | | | | | |
+--HanXRQChr14_79508506_79508881
| | | | | | | | | | | | | | |
+--| (99)
| | | | | | | | | | | | | | |
| +--HanXRQChr15_145112060_145112588
| | | | | | | | | | | | | | |
+--| (100)
| | | | | | | | | | | | | | |
| +--HanXRQChr09_159439793_159440170
| | | | | | | | | | | | | | |
+--| (55)
| | | | | | | | | | | | | | |
| +--HanXRQChr14_45570798_45571311
| | | | | | | | | | | | | | |
+--| (100)
| | | | | | | | | | | | | | |
| | +--HanXRQChr16_170010412_170010812
| | | | | | | | | | | | | | |
| +--| (100)
| | | | | | | | | | | | | | |
| +--HanXRQChr01_83270623_83271135
| | | | | | | | | | | | | | | +--|
(77)
| | | | | | | | | | | | | | | | +--
HanXRQChr10_173822886_173823254
| | | | | | | | | | | | | | | +--|
(75)
| | | | | | | | | | | | | | | | +--
HanXRQChr04_147140553_147141079
| | | | | | | | | | | | | | | +--|
(46)

| | | | | | | | | | | | | | | | |
+--HanXRQChr09_59706805_59707181
| | | | | | | | | | | | | | | | | +--|
(94)
| | | | | | | | | | | | | | | | | |
+--HanXRQChr04_65301484_65302016
| | | | | | | | | | | | | | | | +--|
(85)
| | | | | | | | | | | | | | | |
+--HanXRQChr07_35619467_35619941
| | | | | | | | | | | | | | | +--| (59)
| | | | | | | | | | | | | | | | +--
HanXRQChr02_46053364_46053773
| | | | | | | | | | | | | | +--| (100)
| | | | | | | | | | | | | | +--
HanXRQChr10_100532038_100532438
| | | | | | | | | | | | | +--| (93)
| | | | | | | | | | | | | | | +--
HanXRQChr15_125481128_125481695
| | | | | | | | | | | | | | +--| (100)
| | | | | | | | | | | | | | +--
HanXRQChr10_140012131_140012699
| | | | | | | | | | | | | +--| (85)
| | | | | | | | | | | | | | | +--
HanXRQChr15_153093160_153093558
| | | | | | | | | | | | | | +--| (94)
| | | | | | | | | | | | | | +--
HanXRQChr11_77517539_77518062
| | | | | | | | | | | | | +--| (33)
| | | | | | | | | | | | | | | +--
HanXRQChr17_54907212_54907721
| | | | | | | | | | | | | | +--| (100)
| | | | | | | | | | | | | | +--
HanXRQChr09_50143993_50144471
| | | | | | | | | | | | +--| (40)
| | | | | | | | | | | | +--
HanXRQChr07_67769_68326
| | | | | | | | | | | +--| (53)
| | | | | | | | | | | | +--
HanXRQChr10_170627841_170628410
| | | | | | | | | | +--------------------------| (100)
| | | | | | | | | | +--
HanXRQChr13_127794694_127795300
| | | | | | | | +--| (89)

```
|        | | |    |        |        |        |                 +--HanXRQChr12_94877770_94878735
|        | | |    |        |        |  +--| (95)
|        | | |    |        |        | | |                              +--
HanXRQChr16_150636591_150640613
|        | | |    |        |        | | |                              +--| (100)
|        | | |    |        |        | | |                              | |       +--
HanXRQChr17_47637609_47642782
|        | | |    |        |        | | |                              | |       +--| (92)
|        | | |    |        |        | | |                              | |       |  +--
HanXRQChr09_42030904_42038738
|        | | |    |        |        | | |                              | |  +--| (100)
|        | | |    |        |        | | |                              | | |  +--
HanXRQChr08_88220498_88221890
|        | | |    |        |        | | |                              |  +--| (100)
|        | | |    |        |        | | |                              |          +--
HanXRQChr15_74394306_74402182
|        | | |    |        |        | | |                    +--| (98)
|        | | |    |        |        | | |                    |  +--
HanXRQChr05_177102059_177102727
|        | | |    |        |        | | |                    +--| (98)
|        | | |    |        |        | | |                    |  +--
HanXRQChr05_199634091_199642069
|        | | |    |        |        | | |                    +--| (99)
|        | | |    |        |        | | |                    |  +--HanXRQChr09_26818251_26820777
|        | | |    |        |        | | |                    +--| (70)
|        | | |    |        |        | | |                    |  +--HanXRQChr03_34702985_34703845
|        | | |    |        |        | | |           +--| (97)
|        | | |    |        |        | | |           | |       +--HanXRQChr17_164543226_164545213
|        | | |    |        |        | | |           | |  +--| (93)
|        | | |    |        |        | | |           | | | |  +--HanXRQChr11_5059413_5062302
|        | | |    |        |        | | |           | | |  +--| (100)
|        | | |    |        |        | | |           | | |       +--HanXRQChr16_159464039_159466946
|        | | |    |        |        | | |           |  +--| (100)
|        | | |    |        |        | | |           |        |         +--
HanXRQChr04_4585394_4586317
|        | | |    |        |        | | |           |        |  +--| (100)
|        | | |    |        |        | | |           |        | |  +--HanXRQChr12_8060455_8064354
|        | | |    |        |        | | |           |        +--| (100)
|        | | |    |        |        | | |           |        +--HanXRQChr04_4414273_4418183
|        | | |    |        |        | | |           +--| (100)
|        | | |    |        |        | | |           | |       +--HanXRQChr16_154231921_154234094
|        | | |    |        |        | | |           | |       +--| (100)
|        | | |    |        |        | | |           | |       | |  +--
HanXRQChr10_201208107_201211833
```

```
|       | | |   |       |       | | |           | |     | +--| (100)
|       | | |   |       |       | | |           | |     |       +--
HanXRQChr06_1335657_1337253
|       | | |   |       |       | | |           | | +--| (95)
|       | | |   |       |       | | |           | | | | +--HanXRQChr09_1135754_1136217
|       | | |   |       |       | | |           | | | | +--| (100)
|       | | |   |       |       | | |           | | | | | +--HanXRQChr09_1132214_1133064
|       | | |   |       |       | | |           | | | +--| (99)
|       | | |   |       |       | | |           | | |   | +--
HanXRQChr14_171048254_171055488
|       | | |   |       |       | | |           | | |   +--| (100)
|       | | |   |       |       | | |           | | |   +--HanXRQChr02_147305501_147307299
|       | | |   |       |       | | |           | +--| (96)
|       | | |   |       |       | | |           |     | +--HanXRQChr06_47763415_47764638
|       | | |   |       |       | | |           |     +--| (100)
|       | | |   |       |       | | |           |     +--HanXRQChr12_40589262_40592252
|       | | |   |       |       | | |           +--| (96)
|       | | |   |       |       | | |           | +--HanXRQChr05_52352508_52353847
|       | | |   |       |       | | |           +--| (100)
|       | | |   |       |       | | |           | +--HanXRQChr12_66452661_66454107
|       | | |   |       |       | | |   +--| (100)
|       | | |   |       |       | | |   | +--HanXRQChr03_153476779_153477741
|       | | |   |       |       | | |   +--| (99)
|       | | |   |       |       | | |   | +--HanXRQChr09_192317181_192318492
|       | | |   |       |       | | | +--| (86)
|       | | |   |       |       | | | | +--HanXRQChr09_173686390_173688466
|       | | |   |       |       | | +--| (79)
|       | | |   |       |       | |   |       +--HanXRQChr07_27412393_27412825
|       | | |   |       |       | |   +-------| (100)
|       | | |   |       |       | |           +--HanXRQChr07_27480885_27481317
|       | | |   |       |       +--| (100)
|       | | |   |       |       |       +--HanXRQChr10_167996501_167997306
|       | | |   |       |       | +--| (100)
|       | | |   |       |       | | | +--HanXRQChr07_94263537_94264736
|       | | |   |       |       | | +--| (100)
|       | | |   |       |       | |   |       +--HanXRQChr10_200122501_200126511
|       | | |   |       |       | |   |       +--| (100)
|       | | |   |       |       | |   |       | +--HanXRQChr03_159773788_159776353
|       | | |   |       |       | |   |       +--| (100)
|       | | |   |       |       | |   |       | +--HanXRQChr16_117885695_117887257
|       | | |   |       |       | |   | +--| (85)
|       | | |   |       |       | |   | | +--HanXRQChr16_139387141_139394695
|       | | |   |       |       | |   +--| (100)
|       | | |   |       |       | |   +--HanXRQChr13_70292420_70293133
```

```
|       | | |   |       |       +--| (100)
|       | | |   |       |       |               +--HanXRQChr12_74630615_74638291
|       | | |   |       |       |       +--| (100)
|       | | |   |       |       |       | | +--HanXRQChr05_32799874_32806012
|       | | |   |       |       |       | +--| (100)
|       | | |   |       |       |       |       +--HanXRQChr05_66536036_66536958
|       | | |   |       |       |       +--| (99)
|       | | |   |       |       |       | | +--HanXRQChr09_42108185_42108601
|       | | |   |       |       |       | +--| (100)
|       | | |   |       |       |       |       |       +--HanXRQChr14_70675600_70678361
|       | | |   |       |       |       |       | +--| (99)
|       | | |   |       |       |       |       | | | +--HanXRQChr14_79208902_79211665
|       | | |   |       |       |       |       | | +--| (91)
|       | | |   |       |       |       |       | |     +--HanXRQChr14_77501682_77504443
|       | | |   |       |       |       |       +--| (100)
|       | | |   |       |       |       |       +--HanXRQChr02_175774663_175777915
|       | | |   |       |       |       +--| (100)
|       | | |   |       |       |       | +--HanXRQChr08_40489307_40491198
|       | | |   |       |       |       +--| (81)
|       | | |   |       |       |       | | +--HanXRQChr09_184856661_184857251
|       | | |   |       |       |       | +--| (100)
|       | | |   |       |       |       |       +--HanXRQChr13_144823475_144824100
|       | | |   |       |       | +--| (57)
|       | | |   |       |       | | | +--HanXRQChr15_104285615_104286058
|       | | |   |       |       | | +--| (100)
|       | | |   |       |       | |     +--HanXRQChr05_209135841_209137273
|       | | |   |       |       +--| (63)
|       | | |   |       |       +--HanXRQChr16_128055830_128059471
|       | | |   | +--| (98)
|       | | |   | | +--HanXRQChr11_34832223_34833041
|       | | |   +--| (100)
|       | | |   +--HanXRQChr03_69429483_69433013
|       | +--| (100)
|       |       +--HanXRQChr04_162448231_162451209
| +--| (100)
| | | +--HanXRQChr13_159695079_159696066
| | +--| (100)
| |     +--HanXRQChr06_34593592_34601130
+--| (98)
  |             +--HanXRQChr07_27634064_27638140
  |             +--| (99)
  |             | +--HanXRQChr05_64416693_64422326
  |     +--| (85)
  |     | | +--HanXRQChr15_23210956_23213675
```

```
|       |  +--| (99)
|       |         +--HanXRQChr11_69846093_69853687
|       +--| (98)
|       |  |  +--HanXRQChr06_20451348_20451704
|       |  +--| (97)
|       |         +--HanXRQChr13_179591469_179595652
|       +--| (80)
|       |  |        +--HanXRQChr12_45905678_45909951
|       |  |        +--| (100)
|       |  |        |  +--HanXRQChr15_74224040_74228313
|       |  |  +--| (67)
|       |  |  |  +--HanXRQChr13_2986278_2987762
|       |  +--| (67)
|       |         +--HanXRQChr02_4724981_4726023
|  +--| (100)
|  |  +--HanXRQChr13_135223336_135224153
+--| (100)
       +--HanXRQChr13_196286900_196290049
```

Consensus tree in newick format:

(HanXRQChr11_78963679_78964101:0.0407041958,((((HanXRQChr01_34887426_34894282:
0.0043216127,(HanXRQChr12_24613340_24614343:0.0000022537,HanXRQChr08_22937900
_22939039:0.0000022537)95:0.0000022537)92:0.0000022537,HanXRQChr13_45702842_457
03840:0.0086610080)98:0.0043188648,HanXRQChr17_205330461_205331450:0.0047369591
)98:0.0000025735,HanXRQChr03_26205754_26213308:0.0130666556)100:0.0048626091,(((H
anXRQChr04_67540092_67541320:0.0215906904,((HanXRQChr06_27984911_27988051:0.0
130523062,(HanXRQChr04_12175865_12181403:0.0223508270,((((HanXRQChr17_21132778
8_211333859:0.0130875744,(HanXRQChr05_59284493_59290538:0.0000022537,HanXRQCh
r05_106376295_106382341:0.0000022537)100:0.0270169043)100:0.0148907682,((HanXRQC
hr09_48889131_48889670:0.0525394261,HanXRQChr15_55516101_55518531:0.0430714462
)99:0.0253405579,((((((HanXRQChr03_57480187_57481416:0.0472729932,(HanXRQChr05_1
66779247_166780763:0.0867630203,HanXRQChr12_94875864_94876484:0.1078338990)100
:0.0113747932)80:0.0000022785,(HanXRQChr04_92418435_92419412:0.0044568713,(HanX
RQChr06_53262309_53263292:0.0000022537,HanXRQChr05_9034545_9035531:0.00877214
61)100:0.0042802153)100:0.1907893785)99:0.0118954381,HanXRQChr11_113608046_11360
8769:0.0918252109)99:0.0436748954,((((HanXRQChr16_94477431_94477942:0.1505603506,
((((HanXRQChr17_67579875_67582613:0.0705704174,HanXRQChr11_142203384_14220425
6:0.1224230204)100:0.0970366901,HanXRQChr04_176555457_176557628:0.0486864534)88:
0.0000025328,HanXRQChr01_116026086_116027124:0.0273426427)96:0.0044701809,HanX
RQChr13_137859189_137859594:0.0259877896)100:0.1109987881)96:0.0095491150,HanXR
QChr10_152624866_152626709:0.0606197636)97:0.0221861088,(((HanXRQChr07_57474618
_57475394:0.0178254193,HanXRQChr07_57434299_57438258:0.0264921642)100:0.0085554

375,(HanXRQChr05_190757444_190758598:0.0133384192,(HanXRQChr13_94278579_94280
493:0.0207149214,HanXRQChr04_115355662_115358538:0.0307048521)100:0.0687027690)
100:0.0045744801)100:0.0236252646,(((HanXRQChr11_18867372_18868580:0.0000022537,
HanXRQChr02_20281946_20283260:0.0000022537)73:0.0000022537,HanXRQChr11_187499
00_18751215:0.0043147530)100:0.0367658568,HanXRQChr06_28223014_28227854:0.02547
38083)98:0.0136345033)98:0.0439648850)100:0.1938359137,((HanXRQChr12_88474680_88
475105:0.2931866118,((((HanXRQChr01_1207903_1208488:0.0260071966,(HanXRQChr08_1
51986814_151987353:0.0275982897,HanXRQChr10_94811711_94812246:0.0584141556)100
:0.0419304710)99:0.0182141824,(HanXRQChr05_168861330_168861873:0.0563680311,(Han
XRQChr05_193946694_193947239:0.0212249450,HanXRQChr05_193733456_193734002:0.0
141769226)100:0.0272066595)100:0.0660934620)99:0.0459246388,HanXRQChr05_18228386
0_182284319:0.0972767973)99:0.0764079730,(HanXRQChr17_105500849_105501312:0.061
7044719,((HanXRQChr04_16618909_16619279:0.0383384736,(HanXRQChr01_146764857_1
46765256:0.0085877417,((HanXRQChr03_20364521_20364920:0.0000022537,HanXRQChr16
_185568115_185568603:0.0000022622)43:0.0000022622,HanXRQChr05_14305976_1430637
5:0.0000022537)76:0.0000022622)100:0.4700976608)60:0.0299887631,HanXRQChr03_11331
5527_113315992:0.0979768543)62:0.0256505223)98:0.0825628859)100:0.4703979647)99:0.1
593908517,(((((HanXRQChr02_175754451_175758707:0.0522363276,HanXRQChr13_941617
63_94165162:0.0309730644)93:0.0476628793,(HanXRQChr11_38886892_38889248:0.05711
40913,(HanXRQChr05_164509744_164514326:0.0779891157,HanXRQChr03_34772558_347
73468:0.1549721412)82:0.0308452319)93:0.0472341380)68:0.0295422754,((((HanXRQChr08
_33398524_33406006:0.0270813022,HanXRQChr12_40586043_40589256:0.0260993930)100
:0.0257719003,(HanXRQChr13_77076783_77077636:0.0532649324,(((((HanXRQChr10_1159
66867_115967362:0.0275064060,HanXRQChr14_68245992_68247226:0.0370153066)34:0.00
00022537,HanXRQChr01_49628126_49635945:0.0091026298)47:0.0000022537,(HanXRQChr
09_125961449_125964480:0.0325582453,((HanXRQChr06_28014629_28015962:0.03266574
86,HanXRQChr11_23461540_23465141:0.0135691315)100:0.0046413399,HanXRQChr04_16
6379772_166382442:0.0562977037)76:0.0042229370)70:0.0045944846)45:0.0000022537,Ha
nXRQChr17_164520103_164521773:0.0327696860)76:0.0057405918,((HanXRQChr04_17458
2120_174583103:0.0491440783,HanXRQChr13_135224556_135226019:0.1027102172)99:0.0
229032476,(HanXRQChr07_11945240_11945839:0.0472900098,HanXRQChr05_23977914_2
3979210:0.0670046207)100:0.0177040799)78:0.0104855965)99:0.0119038213)100:0.0409555
744)85:0.0045324648,HanXRQChr03_50693386_50694309:0.0148690036)99:0.0340640102,
HanXRQChr08_40474325_40480066:0.0768331491)98:0.0253930455)91:0.0863217330,(((((((
(HanXRQChr02_24653810_24657437:0.0044902217,(HanXRQChr14_68289895_68297518:0.
0090479945,HanXRQChr14_68273516_68281146:0.0136927985)100:0.0091446896)100:0.00
92896446,((((((HanXRQChr02_147295547_147297741:0.0000022537,HanXRQChr09_142942
803_142944567:0.0000022537)100:0.0091718400,HanXRQChr13_96620660_96621156:0.013
6199356)100:0.0044535043,HanXRQChr04_132271481_132272204:0.0182922795)100:0.013
2211382,HanXRQChr14_153324342_153328229:0.0223024647)100:0.0061393572,HanXRQC
hr10_85192350_85197513:0.0178133881)100:0.0087840814,((HanXRQChr06_97006962_970
10930:0.0135595629,HanXRQChr03_159793635_159795071:0.0091602550)100:0.009189802
5,(((HanXRQChr07_88885423_88893193:0.0090532900,HanXRQChr08_109696851_1096975
93:0.0045106573)47:0.0000022537,HanXRQChr08_39439464_39440150:0.0000022537)100:0

.0275335066,(HanXRQChr01_140346486_140347133:0.0234167357,HanXRQChr16_1178835
70_117885696:0.0087504143)99:0.0046367343)95:0.0090987665)100:0.0714830892)91:0.023
4078138)90:0.0092273032,HanXRQChr10_225800164_225801136:0.0133089061)90:0.03016
65687,((HanXRQChr04_79257170_79260978:0.0319390567,(HanXRQChr09_141496442_141
499885:0.0139988872,HanXRQChr03_70542853_70547885:0.0231731381)85:0.0000022537)
100:0.0311617393,HanXRQChr07_94262118_94263107:0.0518137374)88:0.0065847007)82:0
.0143631309,((((HanXRQChr17_170737481_170744938:0.0113166107,HanXRQChr04_11533
9901_115344808:0.0147664612)88:0.0053907573,HanXRQChr05_177667685_177675472:0.0
504681683)100:0.0102767789,((((((((HanXRQChr13_179578065_179585597:0.0000022537,H
anXRQChr13_179805528_179808054:0.0000022537)100:0.0137137146,(HanXRQChr05_644
22906_64424659:0.0045478502,HanXRQChr13_7315803_7323380:0.0135493291)99:0.00441
86852)56:0.0000022537,HanXRQChr15_23213675_23214340:0.0228052294)61:0.004407056
3,(HanXRQChr11_127530325_127537865:0.0090263545,HanXRQChr12_23644236_2364529
7:0.0136724574)67:0.0000022537)46:0.0000022537,(HanXRQChr14_116526279_116533821:
0.0045564042,HanXRQChr17_205323932_205330231:0.0138261795)75:0.0044685647)92:0.0
225303524,(HanXRQChr17_3057459_3059571:0.0000022537,((HanXRQChr13_169241697_1
69244853:0.0090040820,HanXRQChr01_90452614_90460077:0.0136387140)68:0.000002253
7,(HanXRQChr13_45704069_45710378:0.0042971425,HanXRQChr08_22931465_22937801:0
.0006392099)100:0.0040069648)100:0.0044366062)88:0.0000022093)90:0.0270497576,HanX
RQChr13_196274607_196275703:0.0527428147)100:0.0173905128,((HanXRQChr13_484367
72_48438877:0.0090047934,(((HanXRQChr15_129312466_129317224:0.0044767103,HanXR
QChr02_147809574_147817424:0.0044639459)57:0.0000022537,(HanXRQChr14_171031165
_171032099:0.0041667349,HanXRQChr10_113962499_113970363:0.0149298002)100:0.0770
582218)89:0.0044669191,(HanXRQChr11_23755532_23757425:0.0090059764,HanXRQChr01
_4643702_4650088:0.0044678388)67:0.0000022537)81:0.0000022537)89:0.0089143537,Han
XRQChr08_88222000_88228288:0.0227268137)97:0.0100097111)94:0.0193299248)100:0.03
72671801,HanXRQChr15_150283897_150291604:0.0377314519)91:0.0192373827)99:0.0327
248123,((HanXRQChr03_46527653_46534444:0.0382958344,HanXRQChr06_51391635_5139
5795:0.0240074673)100:0.0027937963,HanXRQChr07_44435735_44436200:0.0286052654)1
00:0.0316110970)99:0.0445196146,HanXRQChr10_167884487_167885281:0.0661735505)70:
0.0240441886,(((HanXRQChr16_149753884_149754258:0.0815051804,HanXRQChr01_98528
860_98530430:0.0544668906)78:0.0131649248,HanXRQChr16_128066409_128067498:0.058
1159343)69:0.0161233282,((HanXRQChr02_20242486_20243525:0.0302468427,HanXRQChr
11_18762949_18764264:0.0157866212)100:0.0180052829,HanXRQChr16_148694596_14870
1069:0.0716893932)100:0.0214965049)72:0.0340902223)75:0.0497665583)85:0.0228841739,(
((HanXRQChr15_23293519_23294345:0.1229441863,(HanXRQChr06_29856663_29857319:0.
1932839320,HanXRQChr17_60426850_60428287:0.8422193739)100:0.3500909999)79:0.033
8930771,((((((((((((((((((((HanXRQChr07_30137334_30143115:0.0089208664,HanXRQChr15_6
435740_6441546:0.0000027400)87:0.0000021495,HanXRQChr05_211734084_211734739:0.0
044571527)100:0.0359942280,HanXRQChr04_4816946_4817774:0.0508628527)67:0.000002
0300,HanXRQChr09_90613212_90613775:0.0472948579)100:0.0708833527,((HanXRQChr14
_164972043_164972420:0.0175835319,(HanXRQChr17_171676962_171677479:0.009031882
4,(HanXRQChr04_11014761_11015279:0.0135654294,HanXRQChr04_11235868_11236386:0
.0135678529)100:0.0044128735)100:0.0049064262)100:0.0133940352,HanXRQChr10_36325

499_36326037:0.0142633882)100:0.0492108242)97:0.0111956150,(HanXRQChr09_16583017
_16583476:0.0103582110,HanXRQChr02_53194187_53194755:0.0174603185)100:0.1000819
988)69:0.0050945851,HanXRQChr15_58262815_58263268:0.1299931075)52:0.0185698062,((
((((HanXRQChr03_53881858_53882406:0.0089612650,HanXRQChr15_50520164_50520709:
0.0000022537)100:0.0042080467,HanXRQChr08_58106590_58107151:0.0138410050)100:0.0
673878372,HanXRQChr04_117734534_117735067:0.0810556833)68:0.0000022969,HanXRQ
Chr07_39909716_39910258:0.1079378518)58:0.0142608418,HanXRQChr11_114599910_114
600468:0.0993873825)53:0.0029748145,((HanXRQChr08_102456485_102456998:0.08130733
16,(HanXRQChr03_6367591_6368162:0.1359892544,(HanXRQChr08_32332017_32332576:0.
0000022537,HanXRQChr05_142623602_142624161:0.0000022537)100:0.0888961679)95:0.0
308956819)95:0.0228907912,(((HanXRQChr10_160816414_160816781:0.0132081621,HanXR
QChr09_205724496_205724897:0.0281169000)99:0.0400324403,HanXRQChr05_5609714_56
10098:0.2033338952)57:0.0000028730,HanXRQChr16_19443969_19444339:0.1007433170)1
00:0.1956087268)60:0.0072040374)34:0.0000022690)37:0.0066493205,((((HanXRQChr01_65
087231_65087766:0.0135357449,HanXRQChr10_26225253_26225786:0.0089658529)100:0.0
281466671,HanXRQChr09_6730323_6730857:0.0342596042)100:0.0485612260,HanXRQChr
04_145024358_145024790:0.0771889056)98:0.0251597714,HanXRQChr12_103007227_1030
07661:0.1259688050)96:0.0324699631)35:0.0030985292,(((((HanXRQChr08_56688339_5668
8696:0.0181768478,(((HanXRQChr03_150064680_150065184:0.0000000000,HanXRQChr10_
93521599_93522103:0.0000000000):0.0000022537,HanXRQChr03_126121322_126121826:0.
0000022537)61:0.0000022537,HanXRQChr10_78375584_78376088:0.0044870128)100:0.018
9623566)96:0.0046258632,HanXRQChr07_12736562_12737068:0.0000022537)100:0.039496
8497,HanXRQChr05_164461706_164462192:0.0534193495)100:0.0700702430,(((HanXRQCh
r13_134302109_134302509:0.0000022537,(HanXRQChr10_95181243_95181772:0.00000225
37,HanXRQChr17_160614824_160615353:0.0000022537)100:0.0133393228)100:0.02643630
52,(((HanXRQChr17_13361200_13361728:0.0178877985,((HanXRQChr12_75917343_759178
73:0.0044172404,HanXRQChr14_86341928_86342457:0.0000022537)100:0.0136216824,Han
XRQChr03_99988684_99989213:0.0320018857)100:0.0042671133)100:0.0044846487,HanXR
QChr17_99228227_99228757:0.0179502554)100:0.0080835099,(HanXRQChr16_15638745_1
5639274:0.0088693812,HanXRQChr05_144936503_144937032:0.0000022537)100:0.0052332
624)92:0.0044255580)51:0.0000022537,HanXRQChr02_114840517_114841046:0.027974514
4)100:0.0920021237)55:0.0097280869,(((HanXRQChr11_55711325_55711859:0.1035322840,
(HanXRQChr01_29157382_29157877:0.1268497522,HanXRQChr13_104941607_104942137:
0.1325712877)98:0.0273235857)73:0.0196876949,(HanXRQChr08_25571094_25571648:0.01
65529013,HanXRQChr05_44360152_44360708:0.0412843691)100:0.1094144289)66:0.01326
66048,HanXRQChr10_187118315_187118736:0.1576481834)53:0.0068269517)56:0.0229910
105)47:0.0049925789,((HanXRQChr09_8264746_8265314:0.0267404261,HanXRQChr16_716
47538_71648109:0.0155378164)100:0.0445479216,(HanXRQChr13_88688183_88688669:0.0
917222415,HanXRQChr17_31920748_31921235:0.0213933895)100:0.1322491446)77:0.0136
853704)42:0.0000025268,((HanXRQChr17_84099735_84100241:0.0654028399,HanXRQChr1
0_11267406_11267923:0.1009088358)99:0.0210591800,(HanXRQChr02_131243878_131244
246:0.0000022537,HanXRQChr10_168209872_168210240:0.0044404989)100:0.0792321392)
84:0.0190850683)57:0.0534881399,((HanXRQChr08_113839738_113840105:0.0182204896,H
anXRQChr04_28158629_28159233:0.0044831219)96:0.0043706095,HanXRQChr11_3983079

2_39831397:0.0046679970)100:0.1571211030)90:0.0595871853,(((HanXRQChr11_13379260
9_133792984:0.0000022537,HanXRQChr11_133775764_133776143:0.0000022537)100:0.035
6166811,HanXRQChr05_62558786_62559318:0.0255330495)100:0.0222587719,(HanXRQChr
09_124910897_124911250:0.0049652384,HanXRQChr11_30817527_30817892:0.017464261
6)100:0.1457239988)100:0.0400045323)64:0.0214460846,(HanXRQChr17_68193643_681941
42:0.0273253482,HanXRQChr11_94539491_94539990:0.0136412292)100:0.1206937594)80:0
.0246065435,(((((HanXRQChr01_40849696_40850225:0.0328551868,HanXRQChr11_135844
467_135845030:0.0130429050)90:0.0000022553,(HanXRQChr16_45835607_45836135:0.017
3236752,HanXRQChr04_141436125_141436654:0.0188198338)100:0.0176600961)100:0.087
3597603,HanXRQChr14_73178118_73178607:0.1877602841)79:0.0170346448,(HanXRQChr0
9_74883466_74883949:0.0208696681,HanXRQChr05_179907532_179908035:0.0342951744)
100:0.1154671547)41:0.0115531844,(HanXRQChr10_170724767_170725228:0.0770535952,(
HanXRQChr11_114441513_114442047:0.0455348797,HanXRQChr08_22016304_22016849:0
.0676965626)98:0.0181142225)95:0.0211556127)25:0.0000029206)39:0.0073887990,(HanXR
QChr09_48002641_48003124:0.0000022537,HanXRQChr09_48019973_48020456:0.0000022
537)100:0.1666970942)34:0.0044908782,((((((((((HanXRQChr17_93185904_93186267:0.0044
886246,HanXRQChr17_93126497_93127118:0.0090084712)56:0.0000022537,HanXRQChr17
_93164704_93165330:0.0182835863)100:0.0225515785,((((HanXRQChr04_162198911_1621
99522:0.0044781802,HanXRQChr17_31095021_31095488:0.0092017795)100:0.0182103604,(
HanXRQChr16_139484437_139485058:0.0135378053,((HanXRQChr06_73271993_73272461:
0.0136794096,(HanXRQChr17_13434430_13434837:0.0183424386,(HanXRQChr05_1714327
14_171433335:0.0089641474,HanXRQChr02_40423347_40423968:0.0136530612)87:0.00000
26456)100:0.0136903227)76:0.0000024927,HanXRQChr10_144765081_144765648:0.090942
9748)93:0.0000029767)60:0.0000025735)89:0.0048349209,(((((HanXRQChr13_193729788_19
3730410:0.0045119723,HanXRQChr02_111025526_111026149:0.0044726689)100:0.0089731
454,HanXRQChr01_83910798_83911405:0.0321259414)93:0.0000023240,(HanXRQChr14_28
912143_28912767:0.0376457070,HanXRQChr11_61241453_61242074:0.0468375832)48:0.00
00022537)84:0.0045373629,(HanXRQChr15_64901712_64902075:0.0000022537,HanXRQChr
04_57314850_57315475:0.0000022537)100:0.0180916675)41:0.0000022537,HanXRQChr02_
40199887_40200279:0.0229112466)82:0.0045383474)51:0.0000028899,HanXRQChr07_9417
4100_94174724:0.0227027255)35:0.0000028899)28:0.0000025735,HanXRQChr17_13932269
1_139323166:0.0416709301)63:0.0043749888,(((HanXRQChr10_145087013_145087418:0.01
84554315,HanXRQChr10_175382210_175382618:0.0137516378)100:0.0138772329,HanXRQ
Chr06_56355680_56356069:0.0473578439)100:0.0094668784,(HanXRQChr17_95938863_95
939232:0.0281952503,HanXRQChr13_144225259_144225863:0.0139789895)99:0.046330781
8)93:0.0044614283)100:0.1505179436,((((HanXRQChr15_52286990_52287513:0.1071387594
,(HanXRQChr09_19029933_19030470:0.0000022537,HanXRQChr09_126494259_126494796:
0.0045066905)100:0.0096266614)100:0.0033667372,HanXRQChr06_41524728_41525252:0.0
262370732)100:0.0748050329,(HanXRQChr03_26626677_26627224:0.0349702369,HanXRQ
Chr12_100245888_100246277:0.0165366065)100:0.0359624588)86:0.0012226583,HanXRQC
hr14_47500657_47501050:0.2172338890)96:0.0192836700)92:0.0288603653,(((((((((HanXRQ
Chr14_79508506_79508881:0.0000022537,HanXRQChr15_145112060_145112588:0.0090526
989)99:0.0090109593,HanXRQChr09_159439793_159440170:0.0277157434)100:0.00454099
37,HanXRQChr14_45570798_45571311:0.0236404762)55:0.0000022537,(HanXRQChr16_170

010412_170010812:0.0276482280,HanXRQChr01_83270623_83271135:0.0182907547)100:0.
0044253086)100:0.0096499194,HanXRQChr10_173822886_173823254:0.0691380201)77:0.0
040435443,HanXRQChr04_147140553_147141079:0.0091115300)75:0.0046470382,((HanXR
QChr09_59706805_59707181:0.0262301631,HanXRQChr04_65301484_65302016:0.0425977
899)94:0.0057049409,HanXRQChr07_35619467_35619941:0.0702092494)85:0.0049376210)4
6:0.0000028899,HanXRQChr02_46053364_46053773:0.0135815773)59:0.0014912951,HanXR
QChr10_100532038_100532438:0.0356718862)100:0.0742665771)53:0.0055089432,(HanXR
QChr15_125481128_125481695:0.0219564806,HanXRQChr10_140012131_140012699:0.005
6875489)100:0.1593110287)93:0.0316511075,(HanXRQChr15_153093160_153093558:0.1306
533000,HanXRQChr11_77517539_77518062:0.0919349496)94:0.0331140556)85:0.02039717
80,(HanXRQChr17_54907212_54907721:0.0286221578,HanXRQChr09_50143993_50144471:
0.0362435551)100:0.0991728469)33:0.0000024974,HanXRQChr07_67769_68326:0.09520835
80)40:0.0096717969)48:0.0077499639,HanXRQChr10_170627841_170628410:0.1044525463)
53:0.0691731378,HanXRQChr13_127794694_127795300:0.1289255920)100:1.5637117117)8
9:0.0374179065,HanXRQChr12_94877770_94878735:0.1677817554)89:0.0444780061)100:0.
5112641593)93:0.0860371938)94:0.0719050964)50:0.0113733593,((((((((((((HanXRQChr16_1
50636591_150640613:0.0223628486,(((HanXRQChr17_47637609_47642782:0.0128816587,H
anXRQChr09_42030904_42038738:0.0232025185)92:0.0000022537,HanXRQChr08_8822049
8_88221890:0.0042288772)100:0.0083642507,HanXRQChr15_74394306_74402182:0.017636
1072)100:0.0133201998)100:0.0234283600,HanXRQChr05_177102059_177102727:0.008181
6387)98:0.0156238353,HanXRQChr05_199634091_199642069:0.0042763879)98:0.00404748
73,HanXRQChr09_26818251_26820777:0.0304360786)99:0.0204372718,HanXRQChr03_347
02985_34703845:0.0400066685)70:0.0037010861,((HanXRQChr17_164543226_164545213:0.
0040630079,(HanXRQChr11_5059413_5062302:0.0000022537,HanXRQChr16_159464039_1
59466946:0.0000022537)100:0.0122053373)93:0.0000022537,((HanXRQChr04_4585394_458
6317:0.0130870475,HanXRQChr12_8060455_8064354:0.0000022537)100:0.0043151508,Han
XRQChr04_4414273_4418183:0.0086004036)100:0.0556171618)100:0.0205390342)97:0.015
4497777,(((HanXRQChr16_154231921_154234094:0.0438290566,(HanXRQChr10_20120810
7_201211833:0.0167186388,HanXRQChr06_1335657_1337253:0.0350255811)100:0.0057250
662)100:0.0267046621,((HanXRQChr09_1135754_1136217:0.0045353280,HanXRQChr09_11
32214_1133064:0.0084705586)100:0.1176414203,(HanXRQChr14_171048254_171055488:0.
0681054474,HanXRQChr02_147305501_147307299:0.0616391530)100:0.1097463175)99:0.0
761454683)95:0.0167380829,(HanXRQChr06_47763415_47764638:0.0362247599,HanXRQC
hr12_40589262_40592252:0.0415001997)100:0.0425572453)96:0.0113887655)100:0.0191233
544,HanXRQChr05_52352508_52353847:0.0507640161)96:0.0222411527,HanXRQChr12_66
452661_66454107:0.0643049151)100:0.0195248039,HanXRQChr03_153476779_153477741:
0.0638817300)100:0.0673121450,HanXRQChr09_192317181_192318492:0.0560190304)99:0.
0132681331,HanXRQChr09_173686390_173688466:0.1037881310)86:0.0072901181,(HanXR
QChr07_27412393_27412825:0.0085723519,HanXRQChr07_27480885_27481317:0.0000022
537)100:0.4689795141)79:0.0239257730)95:0.0336765725,((HanXRQChr10_167996501_167
997306:0.0374874565,(HanXRQChr07_94263537_94264736:0.0149143520,((((HanXRQChr10
_200122501_200126511:0.0082952381,HanXRQChr03_159773788_159776353:0.008419584
5)100:0.0089485771,HanXRQChr16_117885695_117887257:0.0102602554)100:0.020158223
0,HanXRQChr16_139387141_139394695:0.0079149475)85:0.0000028848,HanXRQChr13_70

292420_70293133:0.0229115940)100:0.0375175887)100:0.0683769939)100:0.0965836300,((((((HanXRQChr12_74630615_74638291:0.0301744600,(HanXRQChr05_32799874_32806012:0.0176222204,HanXRQChr05_66536036_66536958:0.0222732528)100:0.0061668589)100:0.0471234159,(HanXRQChr09_42108185_42108601:0.0219502331,((HanXRQChr14_70675600_70678361:0.0043275119,(HanXRQChr14_79208902_79211665:0.0130349843,HanXRQChr14_77501682_77504443:0.0086699495)91:0.0000022537)99:0.0000028315,HanXRQChr02_175774663_175777915:0.0173998122)100:0.0043522188)100:0.0057597173)99:0.0453470725,HanXRQChr08_40489307_40491198:0.0722352566)100:0.0755821603,(HanXRQChr09_184856661_184857251:0.0288868652,HanXRQChr13_144823475_144824100:0.0244184086)100:0.0924244817)81:0.0254552167,(HanXRQChr15_104285615_104286058:0.0515583482,HanXRQChr05_209135841_209137273:0.0759217992)100:0.0448487230)57:0.0123794674,HanXRQChr16_128055830_128059471:0.2006414027)63:0.0282876229)100:0.0958886501)100:0.0611756233)99:0.0516603092)93:0.0000022577,HanXRQChr11_34832223_34833041:0.0337481115)98:0.0020456245,HanXRQChr03_69429483_69433013:0.0155996365)100:0.0116783118)55:0.0000022577)100:0.0139214353,HanXRQChr04_162448231_162451209:0.0312972994)100:0.0322144654)100:0.0231282103,(HanXRQChr13_159695079_159696066:0.0176624069,HanXRQChr06_34593592_34601130:0.0085955876)100:0.0193114800)100:0.0351361834,((((((HanXRQChr07_27634064_27638140:0.0000022537,HanXRQChr05_64416693_64422326:0.0086639752)99:0.0000022537,(HanXRQChr15_23210956_23213675:0.0043518605,HanXRQChr11_69846093_69853687:0.0043551286)99:0.0087133407)85:0.0042855265,(HanXRQChr06_20451348_20451704:0.0000022537,HanXRQChr13_179591469_179595652:0.0087166205)97:0.0000022537)98:0.0086505941,(((HanXRQChr12_45905678_45909951:0.0000022537,HanXRQChr15_74224040_74228313:0.0000022537)100:0.0086658054,HanXRQChr13_2986278_2987762:0.0043211168)67:0.0000022537,HanXRQChr02_4724981_4726023:0.0000022537)67:0.0000022537)80:0.0000022537,HanXRQChr13_135223336_135224153:0.0358325713)100:0.0152305706,HanXRQChr13_196286900_196290049:0.0117574502)100:0.0305133011)98:0.0158379017);

TIME STAMP

Date and time: Wed Mar 11 09:47:29 2020
Total CPU time used: 1543.728984 seconds (0h:25m:43s)
Total wall-clock time used: 1550.515339 seconds (0h:25m:50s)
